# Supplementary material for: Dissemination of information in event-based surveillance, a case study of Avian Influenza
Source: PLoS One. 2023 Sep 5;18(9):e0285341. doi: 10.1371/journal.pone.0285341 (PMC10479896; doi:10.1371/journal.pone.0285341)
Supplement: S1 Table — (DOCX) [file pone.0285341.s001.docx]

**S1 Table.** Definitions to characterize the types of sources, specialization, and geographical focus of PADI-web and HealthMap.

| Specialization | Definition | Example |
| --- | --- | --- |
| Specialized | Focused on the animal health domain | *Avian Flu Diary* |
| General | Focused on all topics, including the animal health domain | *The Guardian* |
| Geographical focus | **Definition** | **Example** |
| Local | Sources that report news at a local or regional scale | *Odisha News Insight* |
| National | Sources that report news at a national scale | *Indian veterinary authority* |
| International | Sources that report news at an international scale | *OIE, Reuters* |
| Type | **Definition** | **Example** |
| International organization | International source for animal disease notification | *OIE, FAO, OMS* |
| Other official authority (local/national) | Sources that have the right to take control measures at the local or a national level | *Ministry, customs, police* |
| Veterinary authority (local/national) | Any sources with the right to take health measures at a local or a national level | *Veterinary authority of Taipei* |
| Online news | Online news media | *Gulf News* |
| Public organization/ association | Public organizations or associations working in the field of health and the environment | *Agricultural Information Network* |
| Laboratory | Laboratory | *Namibia Central Veterinary Laboratory* |
| Private company | Private companies, typically animal industry companies | *Meat production company* |
| Press agency | An agency that gathers news reports and distributes them to other news organizations, such as online news outlets | *Xhinua, Reuters* |
| Radio/television | Radio and television channels | *OdishaTV* |
| Social media | Blogs and social media | *Twitter* |
| EBS tool | EBS tool | *HealthMap, ProMED, PADI-web* |
| A person | A person cited as a source, which cannot be related to the other sources of this list | *A farmer, a local source* |
