# Supplementary material for: Dissemination of information in event-based surveillance, a case study of Avian Influenza
Source: PLoS One. 2023 Sep 5;18(9):e0285341. doi: 10.1371/journal.pone.0285341 (PMC10479896; doi:10.1371/journal.pone.0285341)
Supplement: S2 Table — (DOCX) [file pone.0285341.s002.docx]

**S2 Table.** Summary of the manual curation of the relevance of PADI-web and HealthMap reports.

|  | **PADI-web** | **HealthMap** |
| --- | --- | --- |
| **Relevant** | 337 | 115 |
| **Duplicates** | 28 | 4 |
| **Protective and control measures** | 20 | 21 |
| **Human cases** | 16 | 22 |
| **Global status** | 9 | 2 |
| **Economic consequences** | 5 | 0 |
| **Research** | 4 | 0 |
| **Event before period** | 3 | 3 |
| **Other diseases/irrelevant** | 6 | 0 |
| **Total** | 436 | 167 |
