# Supplementary material for: Dissemination of information in event-based surveillance, a case study of Avian Influenza
Source: PLoS One. 2023 Sep 5;18(9):e0285341. doi: 10.1371/journal.pone.0285341 (PMC10479896; doi:10.1371/journal.pone.0285341)
Supplement: S3 Table — (DOCX) [file pone.0285341.s003.docx]

|  | Type of event | Number of events per week | Number of events per region | | | |
| --- | --- | --- | --- | --- | --- | --- |
|  |  |  | Africa | Americas | Asia | Europe |
| EMPRES-i | Official | 6.9 (2-16) | 27 | 36 | 199 | 89 |
| HealthMap | Official | 3 (1-10) | 4 | 3 | 45 | 8 |
|  | Non-official | 1.5 (1-3) | 0 | 1 | 7 | 0 |
| PADI-web | Official | 13 (1-47) | 22 | 36 | 117 | 24 |
|  | Non-official | 3.7 (1-14) | 1 | 4 | 26 | 0 |

**S3 Table.** Number of reported events reported to the WOAH and detected by the two EBS tools, per week (mean, min and max) and per region.
