# Supplementary material for: Dissemination of information in event-based surveillance, a case study of Avian Influenza
Source: PLoS One. 2023 Sep 5;18(9):e0285341. doi: 10.1371/journal.pone.0285341 (PMC10479896; doi:10.1371/journal.pone.0285341)
Supplement: S8 Table — Proportion of the types of sources according to their role in the (a) PADI-web and (b) HealthMap late detection networks. (DOCX) [file pone.0285341.s008.docx]

| Type of source | Role | n | Proportion |
| --- | --- | --- | --- |
| National vet. auth. | primary | 227 | 0,69 |
| Local vet. auth. | primary | 65 | 0,20 |
| Online news | primary | 16 | 0,05 |
| Local auth. | primary | 8 | 0,02 |
| Laboratory | primary | 5 | 0,02 |
| Radio, TV | primary | 3 | 0,01 |
| National auth. | primary | 2 | 0,01 |
| Research org. | primary | 2 | 0,01 |
| Press agency | primary | 1 | 0 |
| International vet. auth. | primary | 0 | 0 |
| Social platform | primary | 0 | 0 |
| International vet. auth. | secondary | 134 | 0,42 |
| Laboratory | secondary | 0 | 0 |
| Local auth. | secondary | 1 | 0 |
| Local vet. auth. | secondary | 5 | 0,02 |
| National auth. | secondary | 4 | 0,01 |
| National vet. auth. | secondary | 4 | 0,01 |
| Online news | secondary | 99 | 0,31 |
| Press agency | secondary | 56 | 0,17 |
| Radio, TV | secondary | 12 | 0,04 |
| Research org. | secondary | 1 | 0 |
| Social platform | secondary | 2 | 0,01 |

**S8 Table a).** Proportion of the types of sources according to their role in PADI-web late detection network.

**S8 Table b).** Proportion of the types of sources according to their role in HealthMap late detection network.

| Type of source | Role | n | Proportion |
| --- | --- | --- | --- |
| National vet. auth. | primary | 38 | 0,63 |
| Online news | primary | 7 | 0,12 |
| National auth. | primary | 4 | 0,07 |
| Laboratory | primary | 3 | 0,05 |
| Local vet. auth. | primary | 3 | 0,05 |
| Press agency | primary | 3 | 0,05 |
| Radio, TV | primary | 1 | 0,02 |
| Social platform | primary | 1 | 0,02 |
| International vet. auth. | primary | 0 | 0 |
| Local auth. | primary | 0 | 0 |
| Research org. | primary | 0 | 0 |
| International vet. auth. | secondary | 17 | 0,33 |
| Laboratory | secondary | 0 | 0 |
| Local auth. | secondary | 0 | 0 |
| Local vet. auth. | secondary | 0 | 0 |
| National auth. | secondary | 0 | 0 |
| National vet. auth. | secondary | 2 | 0,04 |
| Online news | secondary | 9 | 0,18 |
| Press agency | secondary | 16 | 0,31 |
| Radio, TV | secondary | 1 | 0,02 |
| Research org. | secondary | 1 | 0,02 |
| Social platform | secondary | 5 | 0,10 |
