# Supplementary material for: Dissemination of information in event-based surveillance, a case study of Avian Influenza
Source: PLoS One. 2023 Sep 5;18(9):e0285341. doi: 10.1371/journal.pone.0285341 (PMC10479896; doi:10.1371/journal.pone.0285341)
Supplement: S9 Table — Proportion of the types of sources according to their role in the (a) PADI-web and (b) HealthMap early detection networks. (DOCX) [file pone.0285341.s009.docx]

| Type of source | Role | n | Proportion (%) |
| --- | --- | --- | --- |
| Local vet. auth. | primary | 51 | 0,44 |
| National vet. auth. | primary | 31 | 0,27 |
| Local auth. | primary | 19 | 0,17 |
| Online news | primary | 5 | 0,04 |
| Radio, TV | primary | 3 | 0,03 |
| Private company | primary | 2 | 0,02 |
| Laboratory | primary | 1 | 0,01 |
| National auth. | primary | 1 | 0,01 |
| Press agency | primary | 0 | 0 |
| Social platform | primary | 0 | 0 |
| Laboratory | secondary | 1 | 0,01 |
| Local auth. | secondary | 2 | 0,02 |
| Local vet. auth. | secondary | 3 | 0,03 |
| National auth. | secondary | 0 | 0 |
| National vet. auth. | secondary | 0 | 0 |
| Online news | secondary | 74 | 0,69 |
| Press agency | secondary | 15 | 0,14 |
| Private company | secondary | 0 | 0 |
| Radio, TV | secondary | 8 | 0,07 |
| Social platform | secondary | 3 | 0,03 |

**S9 Table a).** Proportion of the types of sources according to their role in PADI-web early detection network.

| Type of source | Role | n | Proportion (%) |
| --- | --- | --- | --- |
| National vet. auth. | primary | 18 | 0,42 |
| Local vet. auth. | primary | 8 | 0,19 |
| Laboratory | primary | 5 | 0,12 |
| Local auth. | primary | 3 | 0,07 |
| Online news | primary | 3 | 0,07 |
| Press agency | primary | 3 | 0,07 |
| Social platform | primary | 2 | 0,05 |
| National auth. | primary | 1 | 0,02 |
| Private company | primary | 0 | 0 |
| Radio, TV | primary | 0 | 0 |
| Laboratory | secondary | 0 | 0 |
| Local auth. | secondary | 0 | 0 |
| Local vet. auth. | secondary | 0 | 0 |
| National auth. | secondary | 0 | 0 |
| National vet. auth. | secondary | 3 | 0,08 |
| Online news | secondary | 22 | 0,58 |
| Press agency | secondary | 7 | 0,18 |
| Private company | secondary | 0 | 0,00 |
| Radio, TV | secondary | 1 | 0,03 |
| Social platform | secondary | 5 | 0,13 |

**S5 Table b).** Proportion of the types of sources according to their role in HealthMap early detection network.
