# Supplementary material for: Dissemination of information in event-based surveillance, a case study of Avian Influenza
Source: PLoS One. 2023 Sep 5;18(9):e0285341. doi: 10.1371/journal.pone.0285341 (PMC10479896; doi:10.1371/journal.pone.0285341)
Supplement: S10 Table — (DOCX) [file pone.0285341.s010.docx]

|  | **PADI-web** | | **HealthMap** | |
| --- | --- | --- | --- | --- |
|  | Primary | Secondary | Primary | Secondary |
| **Local vet auth.** | 14 | 1 | 2 | 0 |
| **Research org.** | 10 | 2 | 1 | 0 |
| **Online news source** | 7 | 28 | 0 | 4 |
| **Local off. Auth.** | 3 | 0 | 1 | 0 |
| **National off. Auth.** | 3 | 0 | 1 | 0 |
| **Local person** | 2 | 0 | 0 | 0 |
| **Laboratory** | 1 | 0 | 2 | 0 |
| **National vet auth.** | 1 | 0 | 0 | 0 |
| **Private company** | 1 | 0 | 0 | 0 |
| **Radio, TV** | 1 | 2 | 0 | 0 |
| **Press agency** | 0 | 4 | 1 | 3 |

**S10 Table.** Type of primary and secondary sources involved in the detection and transmission of non-official events.
