# Supplementary material for: Dissemination of information in event-based surveillance, a case study of Avian Influenza
Source: PLoS One. 2023 Sep 5;18(9):e0285341. doi: 10.1371/journal.pone.0285341 (PMC10479896; doi:10.1371/journal.pone.0285341)
Supplement: S1 File — (PDF) [file pone.0285341.s011.pdf]

S1 File. Statistical comparison of the sensitivity of PADI-web and HealthMap networks.

```
contingency_table <-
matrix(c(33, 166, 27, 125),
       nrow = 2,
       dimnames = list("HealthMap" = c("Detected", "Not detected"),
                       "PADI-web" = c("Detected", "Not detected")))
print(contingency_table)
```

```
##                 PADI-web
## HealthMap      Detected Not detected
##   Detected           33           27
##   Not detected      166          125
```

```
mcnemar.test(contingency_table, correct=FALSE)
```

```
##
##  McNemar's Chi-squared test
##
## data:  contingency_table
## McNemar's chi-squared = 100.11, df = 1, p-value < 2.2e-16
```
