# Supplementary figures and images for: Dissemination of information in event-based surveillance, a case study of Avian Influenza
Source: PLoS One. 2023 Sep 5;18(9):e0285341. doi: 10.1371/journal.pone.0285341 (PMC10479896; doi:10.1371/journal.pone.0285341)

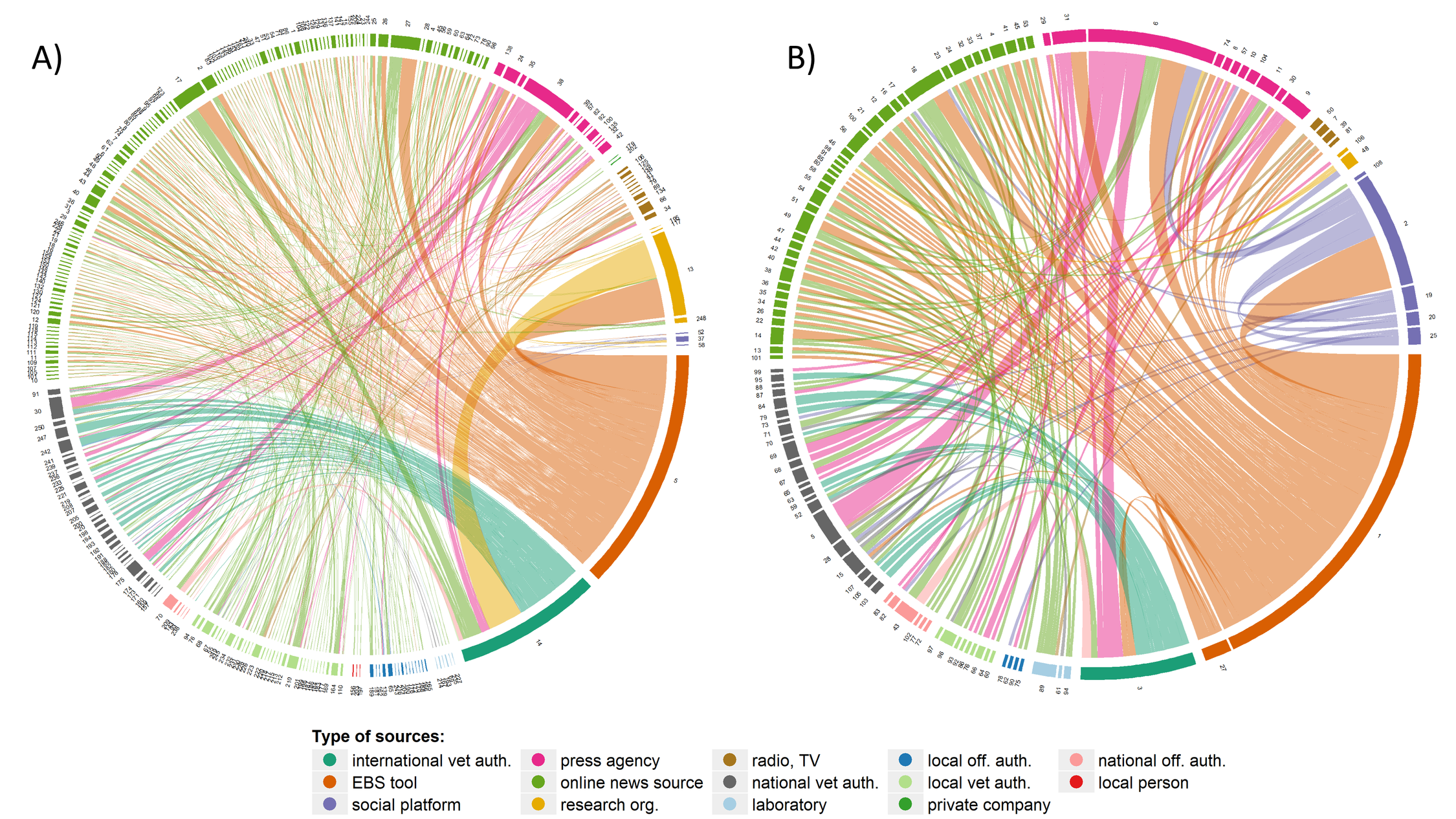

Supplement: S1 Fig — PADI-web (A) and HealthMap (B) networks. Sources were grouped by type. The edge colour corresponds to the colour of the incoming source type, thus enabling the visualisation of the direction of information dissemination, that is, orange edges represent incoming edges to an EBS tool. (TIF) [file pone.0285341.s012.tif]

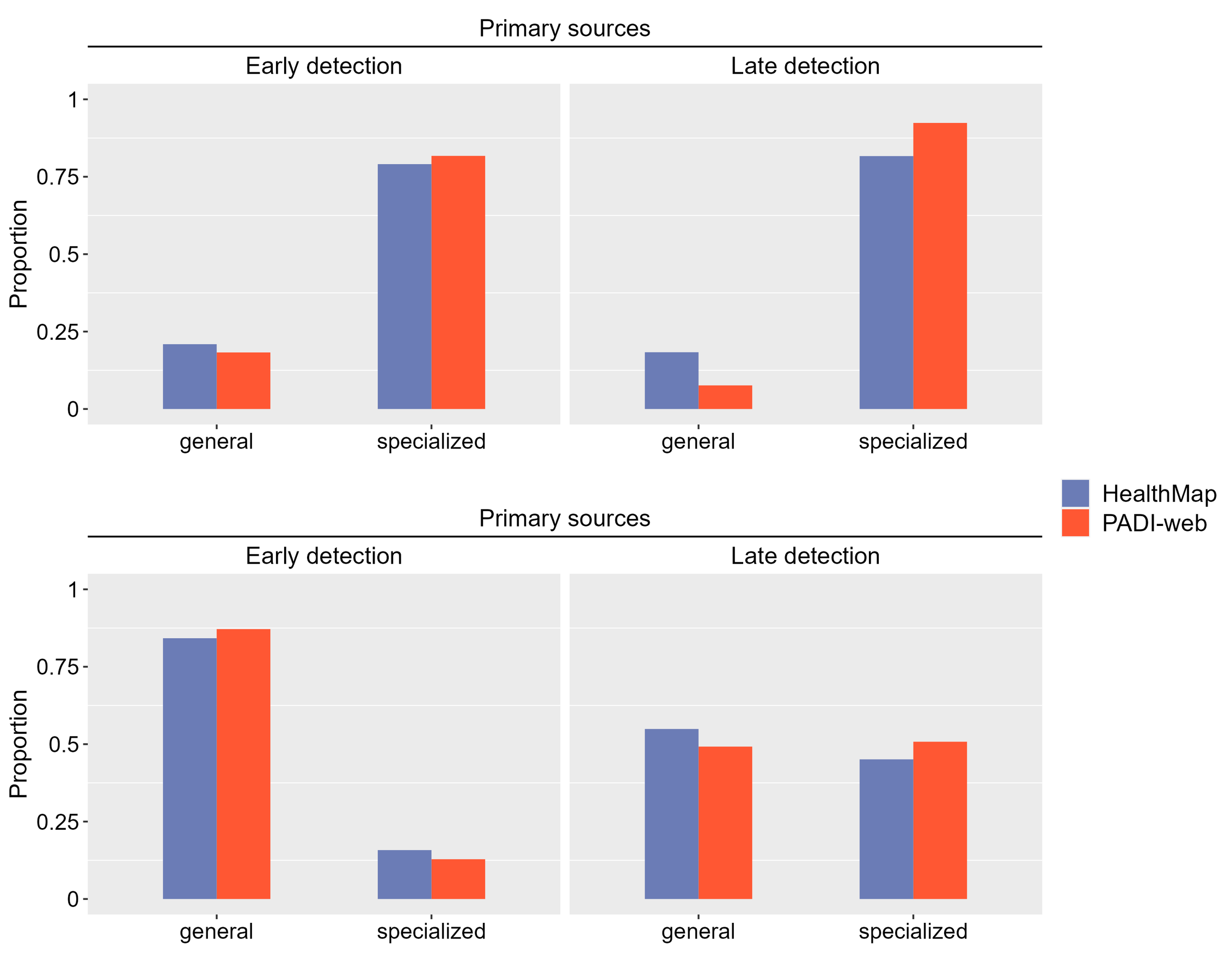

Supplement: S2 Fig — (TIF) [file pone.0285341.s013.tif]
