# Supplementary material for: Dissemination of information in event-based surveillance, a case study of Avian Influenza
Source: PLoS One. 2023 Sep 5;18(9):e0285341. doi: 10.1371/journal.pone.0285341 (PMC10479896; doi:10.1371/journal.pone.0285341)
Supplement: Attachment — Submitted filename: Response to reviewers.pdf [file pone.0285341.s014.pdf]

November 16, 2022

Subject: Response to the review of manuscript number PONE-D-22-24102

Dear PlosOne Chief Editor and Reviewers,

We acknowledge your comments on our manuscript "Dissemination of information in event-based surveillance, a case study of Avian Influenza". We addressed your constructive reviews by modifying our manuscript (using track changes) and answering the reviewers' questions here-below.

Best regards,
The authors

General comments from the editor

If applicable, we recommend that you deposit your laboratory protocols in protocols.io to enhance the reproducibility of your results. Protocols.io assigns your protocol its own identifier (DOI) so that it can be cited independently in the future. For instructions see: `https://journals.plos.org/plosone/s/submission-guidelines#loc-laboratory-protocols`.

1. Please ensure that your manuscript meets PLOS ONE's style requirements, including those for file naming. The PLOS ONE style templates can be found at :

- `https://journals.plos.org/plosone/s/file?id=wjVg/PLOSOne_formatting_sample_main_body.pdf`, and
- `https://journals.plos.org/plosone/s/file?id=ba62/PLOSOne_formatting_sample_title_authors_affiliations.pdf`

- Author affiliations formatting. We have added the appropriate pilcrow symbol for the equal contributors of the work. We have set the appropriate format for the corresponding author. We have fixed the affiliations, by removing postcodes and removing abbreviations of Departments and listing all institutions in full. Please check page 1 of the manuscript.

- Manuscript body formatting. We have adjusted level 1 heading for all major sections. File formats for figures were corrected, now they are in .tiff format and passed via the PACE tool suggested by PlosOne.

2. We note that the grant information you provided in the 'Funding Information' and 'Financial Disclosure' sections do not match. When you resubmit, please ensure that you provide the correct grant numbers for the awards you received for your study in the 'Funding Information' section.

- Done. Funding from Acknowledgments section has been removed and moved into the 'Funding Infor-

mation' and 'Financial Disclosure' sections. Please see the new Acknowledgments section in line 546.

3. Thank you for stating the following in the Acknowledgments Section of your manuscript: "This work has been funded by the "Monitoring outbreak events for disease surveillance in a data science context" (MOOD) project from the European Union's Horizon 2020 research and innovation program under grant agreement No. 874850 (`https://mood-h2020.eu/`) and is catalogued as MOOD 049."

We note that you have provided funding information that is not currently declared in your Funding Statement. However, funding information should not appear in the Acknowledgments section or other areas of your manuscript. We will only publish funding information present in the Funding Statement section of the online submission form.

Please remove any funding-related text from the manuscript and let us know how you would like to update your Funding Statement. Currently, your Funding Statement reads as follows: "The funders had no role in study design, data collection and analysis, decision to publish, or preparation of the manuscript." Please include your amended statements within your cover letter; we will change the online submission form on your behalf.

- Done. Funding from Acknowledgments section has been removed and moved into the 'Funding Information' and 'Financial Disclosure' sections.

- Please continue to use the current Funding Statement: "The funders had no role in study design, data collection and analysis, decision to publish, or preparation of the manuscript."

4. In your Data Availability statement, you have not specified where the minimal data set underlying the results described in your manuscript can be found. PLOS defines a study's minimal data set as the underlying data used to reach the conclusions drawn in the manuscript and any additional data required to replicate the reported study findings in their entirety. All PLOS journals require that the minimal data set be made fully available. For more information about our data policy, please see `http://journals.plos.org/plosone/s/data-availability`. Upon re-submitting your revised manuscript, please upload your study's minimal underlying data set as either Supporting Information files or to a stable, public repository and include the relevant URLs, DOIs, or accession numbers within your revised cover letter. For a list of acceptable repositories, please see `http://journals.plos.org/plosone/s/data-availability#loc-recommended-repositories`. Any potentially identifying patient information must be fully anonymized.

- We created a Zenodo repository (`https://doi.org/10.5281/zenodo.7324144`) containing the entire dataset to reproduce the results. We provided the link in the manuscript, section Data reporting, line 549.

- We also shared the script for our results presented in the manuscript in a public GitHub repository (`https://github.com/SarahVal/EBS-network`). We provided the link in the manuscript, section Statistical reporting, line 552.

- Our dataset does not contain patient information.

Important: If there are ethical or legal restrictions to sharing your data publicly, please explain these restrictions in detail. Please see our guidelines for more information on what we consider unacceptable restrictions to publicly sharing data: `http://journals.plos.org/plosone/s/data-availability#loc-unacceptable-data-access-restrictions`. Note that it is not acceptable for the authors to be the sole named individuals responsible for ensuring data access. We will update your Data Availability statement to reflect the information you provide in your cover letter.

- There are no legal and ethical restrictions for sharing our dataset publicly. Please check the description of our dataset at: `https://doi.org/10.5281/zenodo.6908000`

5. Please upload a new copy of Figure 3 as the detail is not clear. Please follow the link for more information: `https://blogs.plos.org/plos/2019/06/looking-good-tips-for-creating-your-plos-figures-graphics/`

- All figures have passed though the PACE web-based imaging review tool. We provide you with new figure publication graphics in a .tiff format, uploaded separately. For clarity, we have moved Figure 3 into Supp material.

Comments from reviewer 1

Line 35: Please write what WOAH means.

- Done, we defined World Organisation for Animal Health (WOAH, founded as OIE), line 159. We further checked for all other acronyms and their first mention full description.

Line 165: there's a N staring the sentence (also in lines 276 and 278 that are starting with numbers). Please check

- Removed in line 165, it was a typing error. However, we did not find typos for numbers for lines 276 & 278.

Within the results section, what do authors mean by unique events in Table 1?

- A unique event, non-overlapping event, as initially defined in our manuscript, was an event detected by either of the event-based surveillance (EBS) tools, PADI-web or HealthMap. More precisely, a unique event was an event event detected by PADI-web (or by HealthMap, respectively) and not detected by HealthMap (or by PADI-web, respectively). To avoid confusion, we replace the term "unique" by "non-overlapping". Non-overlapping events enable us to analyse the overlap (and, thus, the complementary) between HealthMap and PADI-web. We provide an improved description of the term "unique event" in the manuscript in the section Material and methods, section Event detection line 166 and in the Results, section Event detection lines 266-271.

Figure 3 is impossible to read. Could the authors improve the image quality?

- All figures have passed though the PACE web-based imaging review tool. We provide you with new figure publication graphics in a .tiff format, uploaded separately. For clarity, we have moved Figure 3 into Supp material.

Comments from reviewer 2

Introduction

First paragraph: The manuscript refers to communication in health surveillance and how it can be expanded in the case of avian influenza. Which bibliographic reference of the world health organization that guides or suggests the use of the dissemination of information on health-related events?

- We added references to the Epidemic Intelligence paradigm, which promotes the use of non-official sources to follow the dissemination of information on health-related events and complement indicator-based surveillance. We have in detail reworked the introduction, please check pages 3 and 4.

What context do these Padi-web and HealthMap applications work in? The first paragraphs do not mention health surveillance and its emergencies where these programs/applications can be useful.

- PADI-web and HealthMap facilitate the collection, analysis and dissemination of event-based surveillance data on infectious diseases and associated health issues, in the context of epidemic intelligence. Several studies have assessed their use and performances in different epidemiological contexts including new and enzootic, epizootic and zoonotic infectious diseases. We provide example and new references in the manuscript. We have in detail reworked the introduction, please check pages 3 and 4.

Second paragraph: it is not clear and explanatory all the advantages of using healthy maps descriptors. It must be in simple and clear computational language, after all, the target audience is not only the scientific community, but health workers.

We specified the audience and simplified the description of both tools in the manuscript. We have in detail reworked the introduction, please check pages 3 and 4.

-Seventh paragraph, last line: What is your source of comparison in relation to the healthy map data? what is the assumption or hypothesis that it can be more useful ?

- In the seventh paragraph, we refer to a former study that evaluated the role of the sources detected by HealthMap regarding the detection of outbreaks, at a national scale (Nepal). The gold standard database with which the authors compared HealthMap was the official country outbreak notifications. We motivate our study as an extension of this work, by providing two significant enhancements: (1) we enlarge this work on a global scale and (2) we do not solely rely on the sources directly detected by the EBS tools, but we trace back the origin of the outbreak information. We have in detail reworked the introduction, please check pages 3 and 4.

Regarding the questions of this work

1. What are the sources involved in the reporting of outbreak-related information on the web?- This would not be a question but a methodology to evaluate.

- Every EBS media monitoring tool in use today has its own methodology for detection of sources on the web, collection, filtering of news and extraction of relevant information from the unstructured text from the news. The sources detected by an EBS tool result from (1) the choice of targeting a specific source (e.g. HealthMap collect Pro-MED alerts) and (2) its methodological choices (e.g. keywords to capture the news, languages for the keywords, Google news regions to monitor, etc.). In the last case, the specific online news that will be captured cannot be know *a priori*. In our work, we do not solely evaluate the sources directly detected by the EBS tools, but, we also trace back and characterise the initial sources first emitting the disease outbreak information (referred to as primary sources in our manuscript) and the intermediate ones, based on the manual evaluation of all sources cited in each news, which was a fastidious work of data collection and curation for the co-authors. We provide a clarification on this objective in the introduction.

3. How complementary are the different EBS tools in terms of monitored sources and reported outbreak-related information?—Is it compared to which data?

We address this question in two steps. First, we calculate the proportion of overlapping events (events that were detected by both PADI-web and HealthMap), We show that almost half of the detected events were non-overlapping events. Second, we show that the two tools do not monitor the same sources (i.e. PADI-web retrieved a largest number of online news sources, while HealthMap retrieved content from more social platforms than PADI-web). Please check, the Event detection section in Methods, lines 151-167 and in Results, lines 251-271.

Methodology

Event detection

First paragraph: We chose a one-year 131 study period (July 2018 - June 2019) to capture the space-time epidemiological characteristics of the AI outbreaks around the world.–¿ From which agencies?What sources?

The official data source is described further in our manuscript (Empres-i). Here, we meant that we wanted to embrace a time period enabling us to capture different epizootic events worldwide, to be able to compare the EBS tools and evaluate the network of sources based on a large number of AI outbreaks. Please check lines 151-165.

- We provide a new sentence in the Methods section: "We chose a one-year study period (July 2018 - June 2019) to capture larger scale AI outbreak patterns around the world." Please check lines 128-135.

Define about Empres-i - How it collects health data from official sources?

- We provide a more clear description of the EMPRES-i database, its purpose and its sources. Please

check the Event detection of the Materials and methods section, lines 151-165..

Second paragraph line 145, define what this acronym WOAH means. From this description you can mention only the acronym but not have defined yourself previously

- Done, we provide the full name of the World Organisation for Animal Health (WOAH, ex-OIE). Please check line 159.

Network construction

First paragraph "We assumed that an information pathway could be deducted from the sources cited in a news content. In an information pathway, the first node is called the primary source (i.e. the earliest emitter source), the last node is called the final source (i.e. the final aggregator, PADI-web or HealthMap) and the remaining nodes, if any, are called secondary sources." Comment: It is necessary to modify this definition because primary data in public health and epidemiology are those obtained directly in the territory to be sampled regarding a certain disease data. A secondary data are obtained through the country's information systems.

Epidemic intelligence (EI) encompasses all activities related to early identification of potential health hazards, their verification, assessment and investigation in order to recommend public health control measures. EI integrates both an indicator-based and an event-based component. 'Indicator-based component' refers to structured data collected through routine surveillance systems, corresponding to the definitions provided by the reviewer. 'Event-based component', the context of our study, refers to unstructured data gathered from sources of intelligence of any nature (e.g. media, laboratory, channels of communications, etc.,see `https://www.eurosurveillance.org/content/10.2807/esm.11.12.00665-en`). As noted by the reviewer, the primary sources in terms of diagnosis is usually a laboratory, even in EBS, especially when studying a well-known disease subject to notification as avian influenza. However, this is not true when the detected disease is not yet diagnosed and when solely information about unusual symptoms are communicated. This component of EBS, which is closed to the syndromic surveillance, is an essential component of early detection. In this study, we defined primary sources in EBS paradigm as the earliest cited source of each path, which is not necessarily the primary source in terms of diagnosis, but rather in terms of communication. Thus, it can include official sources typically involved in IBS (laboratory, country's official authorities), as well as informal sources (a person, an company, etc.). We have reworked the introduction, please check pages 3 and 4.

No reference to the global surveillance system by a specific WHO program was cited or used (https://www.who.int/initiatives/global-influenza-surveillance-and-response-system and https://www.who.int/health-topics/influenza-avian-and-other-zoonotic) Why?

Our study lies in the context of event-based surveillance in the animal health domain. We did not described World Health Organization surveillance programs as they mainly focus on zoonotic events from a public health perspective, in the indicator-based paradigm. Besides, our objective was to describe the EBS systems.

Official sources on animal and human surveillance should not be test sources for the network as they are the gold standard for comparing sources of risk communication. In this study, official sources on animal and human surveillance are not tested by themselves. They appeared in the network because they were cited by non-official sources monitored bu the EBS tools. For instance, if an online news sources stated "According to the WHOA, an outbreak of avian influenza was detected yesterday in country X", WHOA was the emitter (primary) source of our network.

Qualitative nodes analysis: Reformulate or change the terms referring to primary and secondary data that cannot refer to the EBS tools technique because they are intrinsically used terms. The terms used must be from epidemiology.

To our knowledge, this work is the first attempt to describe the dissemination of information between sources cited in online news in the context of health surveillance, and no specific terms where proposed to refer to such sources in the epidemiological context. Thus, we proposed the terms primary and secondary as they are explicit for the reader and reflect the temporal diffusion of the events.

How sensitive/specific is the PADI web and Health Map data compared to the gold standard of data? Where are the statistical analyzes showing this fact?

-We calculated the sensitivity of HealthMap and PADI-web, following the definition provided in section Methods. The specificity of event-based surveillance tools cannot be calculated, as it is impossible to assess the status of non-official events they detect; there may be false positive events, as well as true positive events not reported to the gold standard databases (WOAH and EMPRES-i). We did not provide any further statistical tests as the purpose of our study is not to evaluate the influence of factors in the sensitivity of the tools. Please check the apprach and the results in lines 168-181 and 276-278.

As for the geographic scope, it was not clear in the text to the national scope that the data refer. The data should cover the following variables: total number and frequencies of avian influenza events; mean, maximum and minimum value of the number of events monitored per epidemiological week; source and means of event notification; frequency of events monitored by region of occurrence and spatial distribution of events according to reference municipality; opportunity to notification; Closing opportunity (time interval between the date from the notification to the National Surveillance until the end of its monitoring) classification of the group of events according to means of transmission and risk classification after evaluation of the events

For the data from EBS tools, we did not chose any national scope a priori: our data selection was solely based on the studied disease (avian influenza) and host (animals) worldwide. To clarify, we added a table summarizing the total number and frequencies of avian influenza events; mean, maximum and minimum value of the number of events monitored per week; and the source of the event notification as Supplementary material.
