# Supplementary material for: Dissemination of information in event-based surveillance, a case study of Avian Influenza
Source: PLoS One. 2023 Sep 5;18(9):e0285341. doi: 10.1371/journal.pone.0285341 (PMC10479896; doi:10.1371/journal.pone.0285341)
Supplement: Attachment — Submitted filename: PONE-D-22-24102_R1.pdf [file pone.0285341.s015.pdf]

PLOS ONE

Dissemination of information in event-based surveillance, a case study of Avian Influenza

--Manuscript Draft--

**Manuscript Number:**	PONE-D-22-24102R1
**Article Type:**	Research Article
**Full Title:**	Dissemination of information in event-based surveillance, a case study of Avian Influenza
**Short Title:**	Dissemination of information in event-based surveillance
**Corresponding Author:**	Elena Arsevska<br>CIRAD<br>FRANCE
**Keywords:**	event-based surveillance; digital disease detection; network analysis; Avian influenza
**Abstract:**	Event-Based Surveillance (EBS) tools, such as HealthMap and PADI-web, monitor online news reports and other unofficial sources, with the primary aim to provide timely information to users from health agencies on disease outbreaks occurring worldwide. In this work, we describe how outbreak-related information disseminates from a primary source, via a secondary source, to a definitive aggregator, an EBS tool, during the 2018/19 avian influenza season. We analysed 337 news items from the PADI-web and 115 news articles from HealthMap EBS tools reporting avian influenza outbreaks in birds worldwide between July 2018 and June 2019. We used the sources cited in the news to trace the path of each outbreak. We built a directed network with nodes representing the sources (characterised by type, specialisation, and geographical focus) and edges representing the flow of information. We calculated the degree as a centrality measure to determine the importance of the nodes in information dissemination. We analysed the role of the sources in early detection (detection of an event before its official notification) to the World Organisation for Animal Health (WOAH) and late detection.<br>A total of 23% and 43% of the avian influenza outbreaks detected by the PADI-web and HealthMap, respectively, were shared on time before their notification. For both tools, national and local veterinary authorities were the primary sources of early detection. The early detection component mainly relied on the dissemination of nationally acknowledged events by online news and press agencies, bypassing international reporting to the WAOH. WOAH was the major secondary source for late detection, occupying a central position between national authorities and disseminator sources, such as online news. PADI-web and HealthMap were highly complementary in terms of detected sources, explaining why 90% of the events were detected by only one of the tools.<br>We show that current EBS tools can provide timely outbreak-related information and priority news sources to improve digital disease surveillance.
**Order of Authors:**	Sarah Valentin
	Bahdja Boudoua
	Kara Sewalk
	Nejat Arinik
	Mathieu Roche
	Renaud Lancelot
	Elena Arsevska
**Response to Reviewers:**	November 16, 2022<br>Subject: Response to the review of manuscript number PONE-D-22-24102<br>Dear PlosOne Chief Editor and Reviewers,<br>We acknowledge your comments on our manuscript ”Dissemination of information in event-based surveillance,<br>a case study of Avian Influenza”. We addressed your constructive reviews by modifying

our
manuscript (using track changes) and answering the reviewers' questions here-below.
Best regards,
The authors
General comments from the editor
If applicable, we recommend that you deposit your laboratory protocols in protocols.io to enhance the
reproducibility of your results. Protocols.io assigns your protocol its own identifier (DOI) so that it can
be cited independently in the future. For instructions see:
https://journals.plos.org/plosone/s/
submission-guidelines#loc-laboratory-protocols.
1. Please ensure that your manuscript meets PLOS ONE's style requirements, including those for file
naming. The PLOS ONE style templates can be found at :
-
https://journals.plos.org/plosone/s/file?id=wjVg/PLOSOne_formatting_sample_main_body.
pdf, and
-
https://journals.plos.org/plosone/s/file?id=ba62/PLOSOne_formatting_sample_title_authors_
affiliations.pdf
- Author affiliations formatting. We have added the appropriate pilcrow symbol for the equal contributors
of the work. We have set the appropriate format for the corresponding author. We have fixed the
affiliations, by removing postcodes and removing abbreviations of Departments and listing all institutions
in full. Please check page 1 of the manuscript.
- Manuscript body formatting. We have adjusted level 1 heading for all major sections. File formats for
figures were corrected, now they are in .tiff format and passed via the PACE tool suggested by PlosOne.
2. We note that the grant information you provided in the 'Funding Information' and 'Financial Disclosure'
sections do not match. When you resubmit, please ensure that you provide the correct grant
numbers for the awards you received for your study in the 'Funding Information' section.
- Done. Funding from Acknowledgments section has been removed and moved into the 'Funding Information'
and 'Financial Disclosure' sections. Please see the new Acknowledgments section in line 546.
3. Thank you for stating the following in the Acknowledgments Section of your manuscript: "This work
has been funded by the "Monitoring outbreak events for disease surveillance in a data science context"
(MOOD) project from the European Union's Horizon 2020 research and innovation program under grant
agreement No. 874850 (https://mood-h2020.eu/) and is catalogued as MOOD 049."
We note that you have provided funding information that is not currently declared in your Funding
Statement. However, funding information should not appear in the Acknowledgments section or other
areas of your manuscript. We will only publish funding information present in the Funding Statement
section of the online submission form.
Please remove any funding-related text from the manuscript and let us know how you would like to
update your Funding Statement. Currently, your Funding Statement reads as follows: "The funders
had no role in study design, data collection and analysis, decision to publish, or preparation of the

manuscript.” Please include your amended statements within your cover letter; we will change the online
submission form on your behalf.
- Done. Funding from Acknowledgments section has been removed and moved into the ‘Funding Information’
and ‘Financial Disclosure’ sections.
- Please continue to use the current Funding Statement: ”The funders had no role in study design, data
collection and analysis, decision to publish, or preparation of the manuscript.”
4. In your Data Availability statement, you have not specified where the minimal data set underlying
the results described in your manuscript can be found. PLOS defines a study’s minimal data set as
the underlying data used to reach the conclusions drawn in the manuscript and any additional data
required to replicate the reported study findings in their entirety. All PLOS journals require that the
minimal data set be made fully available. For more information about our data policy, please see http:
//journals.plos.org/plosone/s/data-availability. Upon re-submitting your revised manuscript,
please upload your study’s minimal underlying data set as either Supporting Information files or to a
stable, public repository and include the relevant URLs, DOIs, or accession numbers within your revised
cover letter. For a list of acceptable repositories, please see http://journals.plos.org/plosone/s/
data-availability#loc-recommended-repositories. Any potentially identifying patient information
must be fully anonymized.
- We created a Zenodo repository (https://doi.org/10.5281/zenodo.7324144) containing the entire
dataset to reproduce the results. We provided the link in the manuscript, section Data reporting, line
549.
- We also shared the script for our results presented in the manuscript in a public GitHub repository
(https://github.com/SarahVal/EBS-network). We provided the link in the manuscript, section Statistical
reporting, line 552.

- Our dataset does not contain patient information.
Important: If there are ethical or legal restrictions to sharing your data publicly, please explain these
restrictions in detail. Please see our guidelines for more information on what we consider unacceptable restrictions
to publicly sharing data: http://journals.plos.org/plosone/s/data-availability#locunacceptable-
data-access-restrictions. Note that it is not acceptable for the authors to be the sole named individuals responsible for ensuring data access. We will update your Data Availability statement
to reflect the information you provide in your cover letter.
- There are no legal and ethical restrictions for sharing our dataset publicly. Please check the description
of our dataset at: https://doi.org/10.5281/zenodo.6908000
5. Please upload a new copy of Figure 3 as the detail is not clear. Please follow the link for more information:
https://blogs.plos.org/plos/2019/06/looking-good-tips-for-creating-your-plos-figuresgraphics/
- All figures have passed though the PACE web-based imaging review tool. We provide you with new
figure publication graphics in a .tiff format, uploaded separately. For clarity, we have moved Figure 3
into Supp material.

Powered by Editorial Manager® and ProduXion Manager® from Aries Systems Corporation

	Comments from reviewer 1<br>Line 35: Please write what WOAH means.<br>- Done, we defined World Organisation for Animal Health (WOAH, founded as OIE), line 159. We<br>further checked for all other acronyms and their first mention full description.<br>Line 165: there's a N staring the sentence (also in lines 276 and 278 that are starting with numbers).<br>Please check<br>- Removed in line 165, it was a typing error. However, we did not find typos for numbers for lines 276 &<br>278.<br>Within the results section, what do authors mean by unique events in Table 1?<br>- A unique event, non-overlapping event, as initially defined in our manuscript, was an event detected by<br>either of the event-based surveillance (EBS) tools, PADI-web or HealthMap. More precisely, a unique<br>event was an event event detected by PADI-web (or by HealthMap, respectively) and not detected by<br>HealthMap (or by PADI-web, respectively). To avoid confusion, we replace the term ”unique” by ”nonoverlapping”.<br>Non-overlapping events enable us to analyse the overlap (and, thus, the complementary)<br>between HealthMap and PADI-web. We provide an improved description of the term ”unique event” in<br>the manuscript in the section Material and methods, section Event detection line 166 and in the Results,<br>section Event detection lines 266-271.<br>Figure 3 is impossible to read. Could the authors improve the image quality?<br><br>- All figures have passed though the PACE web-based imaging review tool. We provide you with new<br>figure publication graphics in a .tiff format, uploaded separately. For clarity, we have moved Figure 3<br>into Supp material.<br>Comments from reviewer 2<br>Introduction<br>First paragraph: The manuscript refers to communication in health surveillance and how it can be expanded<br>in the case of avian influenza. Which bibliographic reference of the world health organization that<br>guides or suggests the use of the dissemination of information on health-related events?<br>- We added references to the Epidemic Intelligence paradigm, which promotes the use of non-official<br>sources to follow the dissemination of information on health-related events and complement indicatorbased<br>surveillance. We have in detail reworked the introduction, please check pages 3 and 4.<br>What context do these Padi-web and HealthMap applications work in? The first paragraphs do not<br>mention health surveillance and its emergencies where these programs/applications can be useful.<br>- PADI-web and HealthMap facilitate the collection, analysis and dissemination of event-based surveillance<br>data on infectious diseases and associated health issues, in the context of epidemic intelligence.<br>Several studies have assessed their use and performances in different epidemiological contexts including<br>new and enzootic, epizootic and zoonotic infectious diseases. We provide example and new references in<br>the manuscript. We have in detail reworked the introduction, please check pages 3 and 4.<br>Second paragraph: it is not clear and explanatory all the advantages of using healthy maps descriptors. It<br>must be in simple and clear computational language, after all, the target audience is

Powered by Editorial Manager® and ProduXion Manager® from Aries Systems Corporation

not only the scientific
community, but health workers.
We specified the audience and simplified the description of both tools in the manuscript. We have in
detail reworked the introduction, please check pages 3 and 4.
-Seventh paragraph, last line: What is your source of comparison in relation to the healthy map data?
what is the assumption or hypothesis that it can be more useful ?
- In the seventh paragraph, we refer to a former study that evaluated the role of the sources detected
by HealthMap regarding the detection of outbreaks, at a national scale (Nepal). The gold standard
database with which the authors compared HealthMap was the official country outbreak notifications.
We motivate our study as an extension of this work, by providing two significant enhancements: (1) we
enlarge this work on a global scale and (2) we do not solely rely on the sources directly detected by the
EBS tools, but we trace back the origin of the outbreak information. We have in detail reworked the
introduction, please check pages 3 and 4.
Regarding the questions of this work

1. What are the sources involved in the reporting of outbreak-related information on the web?- This would
not be a question but a methodology to evaluate.
- Every EBS media monitoring tool in use today has its own methodology for detection of sources on the
web, collection, filtering of news and extraction of relevant information from the unstructured text from
the news. The sources detected by an EBS tool result from (1) the choice of targeting a specific source
(e.g. HealthMap collect Pro-MED alerts) and (2) its methodological choices (e.g. keywords to capture
the news, languages for the keywords, Google news regions to monitor, etc.). In the last case, the specific
online news that will be captured cannot be know a priori. In our work, we do not solely evaluate the
sources directly detected by the EBS tools, but, we also trace back and characterise the initial sources
first emitting the disease outbreak information (referred to as primary sources in our manuscript) and
the intermediate ones, based on the manual evaluation of all sources cited in each news, which was a
fastidious work of data collection and curation for the co-authors. We provide a clarification on this
objective in the introduction.
3. How complementary are the different EBS tools in terms of monitored sources and reported outbreakrelated
information?—Is it compared to which data?
We address this question in two steps. First, we calculate the proportion of overlapping events (events
that were detected by both PADI-web and HealthMap), We show that almost half of the detected events
were non-overlapping events. Second, we show that the two tools do not monitor the same sources (i.e.
PADI-web retrieved a largest number of online news sources, while HealthMap retrieved content from
more social platforms than PADI-web). Please check, the Event detection section in Methods, lines
151-167 and in Results, lines 251-271.
Methodology
Event detection
First paragraph: We chose a one-year 131 study period (July 2018 - June 2019) to

Powered by Editorial Manager® and ProduXion Manager® from Aries Systems Corporation

capture the spacetime
epidemiological characteristics of the AI outbreaks around the world.–¿ From which agencies?What
sources?
The official data source is described further in our manuscript (Empres-i). Here, we meant that we
wanted to embrace a time period enabling us to capture different epizootic events worldwide, to be able
to compare the EBS tools and evaluate the network of sources based on a large number of AI outbreaks.
Please check lines 151-165.
- We provide a new sentence in the Methods section: ”We chose a one-year study period (July 2018 -
June 2019) to capture larger scale AI outbreak patterns around the world.” Please check lines 128-135.
Define about Empres-i - How it collects health data from official sources?
- We provide a more clear description of the EMPRES-i database, its purpose and its sources. Please

check the Event detection of the Materials and methods section, lines 151-165..
Second paragraph line 145, define what this acronym WOAH means. From this description you can
mention only the acronym but not have defined yourself previously
- Done, we provide the full name of the World Organisation for Animal Health (WOAH, ex-OIE). Please
check line 159.
Network construction
First paragraph “We assumed that an information pathway could be deducted from the sources cited
in a news content. In an information pathway, the first node is called the primary source (i.e. the
earliest emitter source), the last node is called the final source (i.e. the final aggregator, PADI-web or
HealthMap) and the remaining nodes, if any, are called secondary sources.” Comment: It is necessary to
modify this definition because primary data in public health and epidemiology are those obtained directly
in the territory to be sampled regarding a certain disease data. A secondary data are obtained through
the country’s information systems.
Epidemic intelligence (EI) encompasses all activities related to early identification of potential health
hazards, their verification, assessment and investigation in order to recommend public health control measures.
EI integrates both an indicator-based and an event-based component. ‘Indicator-based component’
refers to structured data collected through routine surveillance systems, corresponding to the definitions
provided by the reviewer. ‘Event-based component’, the context of our study, refers to unstructured data
gathered from sources of intelligence of any nature (e.g. media, laboratory, channels of communications,
etc.,see https://www.eurosurveillance.org/content/10.2807/esm.11.12.00665-en). As noted by
the reviewer, the primary sources in terms of diagnosis is usually a laboratory, even in EBS, especially
when studying a well-known disease subject to notification as avian influenza. However, this is not true
when the detected disease is not yet diagnosed and when solely information about unusual symptoms are
communicated. This component of EBS, which is closed to the syndromic surveillance, is an essential
component of early detection. In this study, we defined primary sources in EBS paradigm as the earliest

cited source of each path, which is not necessarily the primary source in terms of diagnosis, but rather
in terms of communication. Thus, it can include official sources typically involved in IBS (laboratory,
country's official authorities), as well as informal sources (a person, an company, etc.). We have reworked
the introduction, please check pages 3 and 4.
No reference to the global surveillance system by a specific WHO program was cited or used (https: //
www. who. int/ initiatives/ global-influenza-surveillance-and-response-system and https:
// www. who. int/ health-topics/ influenza-avian-and-other-zoonotic) Why?
Our study lies in the context of event-based surveillance in the animal health domain. We did not
described World Health Organization surveillance programs as they mainly focus on zoonotic events
from a public health perspective, in the indicator-based paradigm. Besides, our objective was to describe
the EBS systems.

Official sources on animal and human surveillance should not be test sources for the network as they are
the gold standard for comparing sources of risk communication. In this study, official sources on animal
and human surveillance are not tested by themselves. They appeared in the network because they were
cited by non-official sources monitored bu the EBS tools. For instance, if an online news sources stated
"According to the WHOA, an outbreak of avian influenza was detected yesterday in country X", WHOA
was the emitter (primary) source of our network.
Qualitative nodes analysis: Reformulate or change the terms referring to primary and secondary data
that cannot refer to the EBS tools technique because they are intrinsically used terms. The terms used
must be from epidemiology.
To our knowledge, this work is the first attempt to describe the dissemination of information between
sources cited in online news in the context of health surveillance, and no specific terms where proposed to
refer to such sources in the epidemiological context. Thus, we proposed the terms primary and secondary
as they are explicit for the reader and reflect the temporal diffusion of the events.
How sensitive/specific is the PADI web and Health Map data compared to the gold standard of data?
Where are the statistical analyzes showing this fact?
-We calculated the sensitivity of HealthMap and PADI-web, following the definition provided in section
Methods. The specificity of event-based surveillance tools cannot be calculated, as it is impossible to
assess the status of non-official events they detect; there may be false positive events, as well as true
positive events not reported to the gold standard databases (WOAH and EMPRES-i). We did not
provide any further statistical tests as the purpose of our study is not to evaluate the influence of factors
in the sensitivity of the tools. Please check the apprach and the results in lines 168-181 and 276-278.
As for the geographic scope, it was not clear in the text to the national scope that the data refer. The
data should cover the following variables: total number and frequencies of avian influenza events; mean,
maximum and minimum value of the number of events monitored per epidemiological week; source and

	means of event notification; frequency of events monitored by region of occurrence and spatial distribution<br>of events according to reference municipality; opportunity to notification; Closing opportunity (time<br>interval between the date from the notification to the National Surveillance until the end of its monitoring)<br>classification of the group of events according to means of transmission and risk classification after<br>evaluation of the events<br>For the data from EBS tools, we did not chose any national scope a priori: our data selection was solely<br>based on the studied disease (avian influenza) and host (animals) worldwide. To clarify, we added a<br>table summarizing the total number and frequencies of avian influenza events; mean, maximum and<br>minimum value of the number of events monitored per week; and the source of the event notification as<br>Supplementary material.
**Additional Information:**	
**Question**	**Response**
**Financial Disclosure**<br><br>Enter a financial disclosure statement that describes the sources of funding for the work included in this submission. Review the submission guidelines for detailed requirements. View published research articles from *PLOS ONE* for specific examples.<br><br>This statement is required for submission and **will appear in the published article** if the submission is accepted. Please make sure it is accurate.<br><br>**Unfunded studies**<br>Enter: *The author(s) received no specific funding for this work.*<br><br>**Funded studies**<br>Enter a statement with the following details:<br>• Initials of the authors who received each award<br>• Grant numbers awarded to each author<br>• The full name of each funder<br>• URL of each funder website<br>• Did the sponsors or funders play any role in the study design, data collection and analysis, decision to publish, or preparation of the manuscript?<br>• **NO** - Include this sentence at the end of your statement: *The funders had no role in study design, data collection and analysis, decision to publish, or preparation of the manuscript.*<br>• **YES** - Specify the role(s) played.	The funders had no role in study design, data collection and analysis, decision to publish, or preparation of the manuscript.

* typeset	
**Competing Interests**<br><br>Use the instructions below to enter a competing interest statement for this submission. On behalf of all authors, disclose any competing interests that could be perceived to bias this work—acknowledging all financial support and any other relevant financial or non-financial competing interests.<br><br>This statement is required for submission and **will appear in the published article** if the submission is accepted. Please make sure it is accurate and that any funding sources listed in your Funding Information later in the submission form are also declared in your Financial Disclosure statement.<br><br>View published research articles from *PLOS ONE* for specific examples.<br><br>**NO authors have competing interests**<br><br>Enter: *The authors have declared that no competing interests exist.*<br><br>**Authors with competing interests**<br><br>Enter competing interest details beginning with this statement:<br><br>*I have read the journal's policy and the authors of this manuscript have the following competing interests: [insert competing interests here]*<br><br>* typeset	The authors have declared that no competing interests exist.
**Ethics Statement**<br><br>Enter an ethics statement for this submission. This statement is required if the study involved:	N/A

- Human participants
- Human specimens or tissue
- Vertebrate animals or cephalopods
- Vertebrate embryos or tissues
- Field research

Write "N/A" if the submission does not require an ethics statement.

General guidance is provided below. Consult the submission guidelines for detailed instructions. **Make sure that all information entered here is included in the Methods section of the manuscript.**

Powered by Editorial Manager® and ProduXion Manager® from Aries Systems Corporation

**Format for specific study types**<br><br>**Human Subject Research (involving human participants and/or tissue)**<br>• Give the name of the institutional review board or ethics committee that approved the study<br>• Include the approval number and/or a statement indicating approval of this research<br>• Indicate the form of consent obtained (written/oral) or the reason that consent was not obtained (e.g. the data were analyzed anonymously)<br><br>**Animal Research (involving vertebrate animals, embryos or tissues)**<br>• Provide the name of the Institutional Animal Care and Use Committee (IACUC) or other relevant ethics board that reviewed the study protocol, and indicate whether they approved this research or granted a formal waiver of ethical approval<br>• Include an approval number if one was obtained<br>• If the study involved *non-human primates*, add *additional details* about animal welfare and steps taken to ameliorate suffering<br>• If anesthesia, euthanasia, or any kind of animal sacrifice is part of the study, include briefly which substances and/or methods were applied<br><br>**Field Research**<br><br>Include the following details if this study involves the collection of plant, animal, or other materials from a natural setting:<br>• Field permit number<br>• Name of the institution or relevant body that granted permission	
**Data Availability**<br><br>Authors are required to make all data underlying the findings described fully available, without restriction, and from the time of publication. PLOS allows rare exceptions to address legal and ethical concerns. See the PLOS Data Policy and FAQ for detailed information.	Yes - all data are fully available without restriction

A Data Availability Statement describing where the data can be found is required at submission. Your answers to this question constitute the Data Availability Statement and **will be published in the article**, if accepted.<br><br>**Important:** Stating 'data available on request from the author' is not sufficient. If your data are only available upon request, select 'No' for the first question and explain your exceptional situation in the text box.<br><br>Do the authors confirm that all data underlying the findings described in their manuscript are fully available without restriction?	
**Describe where the data may be found in full sentences. If you are copying our sample text, replace any instances of XXX with the appropriate details.**<br><br>• If the data are **held or will be held in a public repository**, include URLs, accession numbers or DOIs. If this information will only be available after acceptance, indicate this by ticking the box below. For example: *All XXX files are available from the XXX database (accession number(s) XXX, XXX.)*.<br>• If the data are all contained **within the manuscript and/or Supporting Information files**, enter the following: *All relevant data are within the manuscript and its Supporting Information files.*<br>• If neither of these applies but you are able to provide **details of access elsewhere**, with or without limitations, please do so. For example:<br><br>*Data cannot be shared publicly because of [XXX]. Data are available from the XXX Institutional Data Access / Ethics Committee (contact via XXX) for researchers who meet the criteria for access to confidential data.*<br><br>*The data underlying the results presented in the study are available from (include the name of the third party*	***NOTE TO PA @ ACCEPT: Please confirm DAS with AU***<br>Data used in this paper are available from the Zenado database at doi (https://doi.org/10.5281/zenodo.6908000). The script for our results presented in the paper are available in a public GitHub repository (https://github.com/SarahVal/EBS-network).

and contact information or URL).<br>• This text is appropriate if the data are owned by a third party and authors do not have permission to share the data.<br><br>* typeset	
Additional data availability information:	Tick here if the URLs/accession numbers/DOIs will be available only after acceptance of the manuscript for publication so that we can ensure their inclusion before publication.

Powered by Editorial Manager® and ProduXion Manager® from Aries Systems Corporation

Revised manuscript
Click here to access/download;Manuscript;Revised manuscript.docx

Dissemination of information in event-based surveillance, a case study of Avian Influenza

Sarah Valentin[1,2,3,4¶], Bahdja Boudoua[1,2¶], Kara Sewalk[5], Nejat Arınık[2], Mathieu Roche[1,2,3], Renaud Lancelot[1,3], Elena Arsevska[1,3*]

[1] Joint Research Unit Animal, Health, Territories, Risks, Ecosystems (UMR ASTRE), French Agricultural Research Centre for International Development (CIRAD), National Research Institute for Agriculture, Food and Environment (INRAE), Montpellier, France

[2] Joint Research Unit Land, Environment, Remote Sensing and Spatial Information (UMR TETIS), Université de Montpellier, AgroParisTech, French Agricultural Research Centre for International Development (CIRAD), French National Centre for Scientific Research (CNRS), National Research Institute for Agriculture, Food and Environment (INRAE), Montpellier, France

[3] French Agricultural Research Centre for International Development (CIRAD), Montpellier, France

[4] Département de biologie, Université de Sherbrooke, Sherbrooke, Canada

[5] Computational Epidemiology Group, Boston Children's Hospital, Boston, United States

[¶] These authors contributed equally to this work

*Corresponding author
E-mail: elena.arsevska@cirad.fr (EA)

ORCID affiliations

Sarah Valentin https://orcid.org/0000-0002-9028-681X

Bahdja Boudoua https://orcid.org/0000-0001-6174-3252

Kara Sewalk https://orcid.org/0000-0002-2917-0869

Nejat Arınık https://orcid.org/0000-0001-5080-4320

Mathieu Roche https://orcid.org/0000-0003-3272-8568

Renaud Lancelot https://orcid.org/0000-0002-5826-5242

Elena Arsevska https://orcid.org/0000-0002-6693-2316

// Abstract

Event-Based Surveillance (EBS) tools, such as HealthMap and PADI-web, monitor online news reports and other unofficial sources, with the primary aim to provide timely information to users from health agencies on disease outbreaks occurring worldwide.

In this work, we describe how outbreak-related information disseminates from a primary source, via a secondary source, to a definitive aggregator, an EBS tool, during the 2018/19 avian influenza season. We analysed 337 news items from the PADI-web and 115 news articles from HealthMap EBS tools reporting avian influenza outbreaks in birds worldwide between July 2018 and June 2019. We used the sources cited in the news to trace the path of each outbreak. We built a directed network with nodes representing the sources (characterised by type, specialisation, and geographical focus) and edges representing the flow of information. We calculated the degree as a centrality measure to determine the importance of the nodes in information dissemination. We analysed the role of the sources in early detection (detection of an event before its official notification) to the World Organisation for Animal Health (WOAH) and late detection.

A total of 23% and 43% of the avian influenza outbreaks detected by the PADI-web and HealthMap, respectively, were shared on time before their notification. For both tools, national and local veterinary authorities were the primary sources of early detection. The early detection component mainly relied on the dissemination of nationally acknowledged events by online news and press agencies, bypassing international reporting to the WAOH. WOAH was the major secondary source for late detection, occupying a central position between national authorities and disseminator sources, such as online news. PADI-web and HealthMap were highly complementary in terms of detected sources, explaining why 90% of the events were detected by only one of the tools.

We show that current EBS tools can provide timely outbreak-related information and priority news sources to improve digital disease surveillance.

Keywords: event-based surveillance, digital disease detection, network analysis, avian influenza

Introduction

Recent developments in internet and digital technologies have contributed to the establishment of the Epidemic Intelligence (EI) framework, aiming at the early identification of potential health threats from sources of intelligence of any nature, their verification, and assessment for timely prevention and control by public and animal health (PH/AH) agencies. Event-based surveillance (EBS), as part of the EI, gathers unstructured data on potential and non-verified disease outbreaks mainly by monitoring the web, such as online media, social networks, and blogs. The EBS is complementary to traditional, indicator-based surveillance (IBS), also part of the EI, which collects structured data on verified disease outbreaks through routine national surveillance systems (1–3).

Since the early 2000s, several automatised EBS tools with open-access have been created, such as HealthMap, operating since 2006 and monitoring web sources for the public, animal, and plant health threats (4), and PADI-web, operating since 2016 and monitoring web sources for mainly animal health threats (5). The two open-access tools are used for the detection and monitoring of potential outbreaks reported in non-official sources on the web, including known diseases, such as avian influenza or Ebola (6,7), or clinical signs of unknown origin, such as acute respiratory syndrome (8). The main users of the two tools are EI staff at national and supranational PH/AH agencies and organizations, among others such as the French Platform for epidemiological surveillance in animal health (Platform ESA) (7) and the European Centre for Disease Control (ECDC) (9).

Both HealthMap and PADI-web implement algorithms to capture news on potential disease outbreaks from a broad range of data sources on the web in multiple languages and geographical regions (4,5). For example, HealthMap gathers data from Baidu, SoSo, Google News aggregators, and ProMED-mail in nine languages. PADI-web collects data from the Google News aggregator in 16 languages. Both tools further implement classification and information extraction algorithms to filter and extract the relevant outbreak information in a structured format from the free text, such as the place, date, and host of a described outbreak. Finally, HealthMap provides users with a world map interface to visualise the reports and information sources that report outbreaks. PADI-web provides users with a list of information sources and news content that reports outbreaks.

Previous evaluations of the EBS tools in use today, including HealthMap and PADI-web, focused mainly on the assessment of their extrinsic performance, such as timeliness, positive predictive value, or sensitivity (Se) in detecting outbreaks from the sources they monitor,

compared to official disease outbreaks (6,7). From an end-user perspective, Barboza et al. (10,11) assessed metrics such as the usefulness, simplicity, and flexibility of an EBS tool.

The understanding of the role of the inputs (i.e. the monitored sources) on the performance of EBS tools is less explored. Barboza et al., 2014 (10) found that the type of moderation, sources, languages, regions of occurrence, and types of cases influence EBS tool performance. Schwind et al. (2017) (12) identified that domestic and national news sources were more likely to report outbreaks than international news portals.

This study aimed to fill the existing gap in the role of sources monitored by EBS tools. We consider EBS tools as aggregators which collect disease outbreak information at the end of a transmission chain, referred to as a network. More precisely, we aimed to characterise the sources of outbreak information detected by an EBS tool and assess how the sanitary information circulates through the monitored sources before being detected by an EBS tool.

We assessed the flow of outbreak information from primary sources, providers of the information, until the end sources, EBS tools, and final aggregators of the information. We represent this information flow through a network structure. Moreover, we provide an in-depth analysis of the extracted networks and the characteristics of the sources involved in outbreak reporting using two EBS tools, HealthMap and PADI-web. In this study, we address three main questions:

1. What are the sources involved in the reporting of outbreak-related information on the web?

2. What are the roles of the different sources regarding the dissemination of outbreak-related information on the web, and what are their characteristics in terms of type, specialisation, and geographical scope?

3. How complementary are the different EBS tools in terms of monitored sources and reported outbreak-related information?

In this study, we further propose a new representation of the sources and their networks involved in digital disease surveillance to improve the detection and analysis of signals of disease emergence from online media. This representation and associated analysis address these questions.

The remainder of this paper is organised as follows. First, we summarise the objectives and methods of assessing information dissemination across data (news) sources. Next, we detail our methodology to collect and assess the dissemination of outbreak-related information via

PADI-web and HealthMap. We present and discuss our results in Section 3, before summarising the main conclusions of our work.

Materials and methods

Data collection

To conduct this study, we chose to analyse news reports of Avian Influenza (AI) detected by two EBS tools, PADI-web and HealthMap. AI viruses can spread over long distances via trade in poultry and wild-caught birds, as well as via the movement of wild birds (13). AI outbreaks are responsible for significant economic losses resulting from trade restrictions, loss of disease-free status for affected countries, or culling measures in infected flocks. Moreover, AI has great zoonotic potential, as some subtypes can infect different avian and mammalian animal hosts, including humans (14). Thus, early detection of AI outbreaks is essential for implementing protection and control measures and helping contain their spread.

For our study, we extracted all English news reports from PADI-web and HealthMap EBS tools, which described one or several AI outbreaks and were published between 1 July 2018 and 30 June 2019 (i.e. 337 news reports from PADI-web and 115 news reports from HealthMap). We chose a one-year study period (July 2018 to June 2019) to capture the spatiotemporal epidemiological characteristics of AI outbreaks worldwide. The detection of the virus at a specific date and time is hereafter referred to as an event (most events are outbreaks, but some describe the detection of the virus in the environment). Two epidemiologists (BB, SV, authors of this work) manually assessed the relevance of each news item (a report was considered relevant if it contained at least one event) and discarded irrelevant news. Importantly, the events can be either reported as confirmed or suspected, as one of the keystones of EI is the detection of potential outbreaks before official confirmation.

Event detection

Two epidemiologists (BB and SV, authors of this work) read the relevant news and identified all reported events. Each event described in the detected news was classified as official or non-official.

Official events corresponded to outbreaks officially notified by AH authorities. For this purpose, we used the Emergency Prevention System for Priority Animal and Plant Pests and Diseases (EMPRES-i), a global animal health information system (15,16) developed by the Food and Agriculture Organization (FAO) of the United Nations. EMPRES-i allows free access

to and sharing of disease outbreak data to support data analysis and notification to national AH authorities by monitoring and summarising the global status of priority animal diseases and zoonoses, including AI. One of the main sources of information for the EMPRES-i is the verified disease outbreak data provided by national AH authorities, mainly through traditional disease surveillance by the World Organisation for Animal Health (WOAH). The EMPRES-i has tracked AI outbreaks since 2003.

When an event could not be linked to an official event from the EMPRES-i, we labelled it as non-official and recorded the epidemiological information provided in the report (i.e. subtype, reported date of the event, the country and location of the event, the host affected, and the number of cases). This enabled us to identify when the same non-official event was reported [illegible] different news articles.

We evaluated the Se? What is this?There is no no acronym that explains it

For both official and non-official events, we calculated the number of non-overlapping events between the two EBS tools, that is, the events that were detected by one tool out of two.

For the official events, we evaluated the Se and timeliness of each tool. Timeliness is the lag in days between the date of official notification to the WOAH (day 0), as recorded in the EMPRES-i database, and the date when the same event was first detected by the PADI-web and HealthMap. A negative lag means that the EBS tool detects an event in a timely manner, that is, before the date of notification. A positive lag indicated that the EBS tool was untimely for detecting an outbreak, that is, the same day or after the official notification date. Se is defined as the ability of the EBS tool to report an event present in the EMPRES-i database, corresponding to the proportion of true positive events (TP) among the sum of true positive and false-negative (FN) events (Se=TP/(TP+FN)). A TP event was defined as all AI outbreaks in the EMPRES-i database during the study period. An FN event was defined as an event present in the EMPRES-i database that was not detected by an EBS tool. The specificity of event-based surveillance tools cannot be calculated, as it is impossible to assess the status of non-official events detected (11); there may be false positive events as well as TP events not reported to the gold standard databases (WOAH and EMPRES-i).

Network construction

To trace back the primary sources, we manually traced the information pathways of all events mentioned in the PADI-web and HealthMap news. We assumed that an information pathway could be deduced from the sources cited in the news content. In the information pathway, the first node is called the primary source (i.e. the earliest emitter source), the last node is

called the final source (i.e. the final aggregator, PADI-web, or HealthMap), and the remaining nodes, if any, are called secondary sources. The combination of all information pathways from news events gives a network structure, referred to as a network of information pathways.

Let *G* = (*V, E, A*) be a directed unweighted attributed graph representing a network of information pathways, where V, E, and A are the set of network nodes, network edges, and attributes associated with the nodes, respectively (17). The network nodes represent the sources and final aggregators (PADI-web and HealthMap). Each node has three attributes, as defined in S1 Table: type (e.g. online news source, national veterinary authority, etc.), geographical focus (local, national, or international), and specialisation in animal health news coverage (general or specialised). The edges represent the dissemination of event information between two nodes (an emitter source, S_E that sends the event, and a receptor source, S_R that receives the event). The graph is directed as the information is transmitted from the S_E to the S_R. A directed graph is formally defined as a graph G for which each edge in *E* has an ordering to its vertices (i.e. such that $e_1 = (u,v)$ is distinct from $e_2 = (v,u)$, for $e_1, e_2 \in E$). In our approach, the edges are not weighed because we create an edge between an S_E and S_R if S_R cites S_E at least once.

It is worth noting that an event can be transmitted through several paths and that a path can transmit several events. The first case occurs when the same event is reported by different sources (e.g. two online news articles). The second occurs when a single news article reports several events. Based on this fact, we separated the global graph into three subgraphs depending on the type of events detected and their timeliness: a graph containing the paths associated with the early detection of official events (timeliness < 0), a graph containing the paths associated with the late detection of official events (timeliness ≥ 0), and a graph containing the paths associated with the detection of non-official events.

Network analysis

Network description

We first describe the network of information pathways extracted from the PADI-web and HealthMap news, PADI-web, and HealthMap networks hereafter, in terms of the number of edges, nodes, and paths. We visualised the networks using a chord diagram and classified the nodes according to their source types.

Path analysis

To evaluate the network performance regarding the dissemination of health events, we calculated the path length and reactivity of the networks. The path length is the number of

edges in the path. The path length corresponds to the number of secondary sources between the primary and final aggregators (PADI-web or HealthMap); for example, a path composed of three edges contain two secondary sources. We hypothesised that the fewer the number of sources in a path, the faster the transmission of information.

Path reactivity is the sum of the time lags between all the nodes composing the path. Path reactivity measures the number of days between the primary source's communication and detection by the final aggregator. Path reactivity is highly relevant for EI because it reflects the ability of the system to quickly disseminate events to the aggregator.

Node analysis

We assessed the importance of the nodes, i.e., the sources, in the PADI-web and HealthMap networks using qualitative and quantitative attributes.

We first evaluated the global ability of the sources to receive and transmit event information by merging PADI-web and HealthMap networks. We calculated the in-degree, out-degree, and all-degree centrality measures of nodes (18) and analysed their distribution according to the type of source. In-degree is the number of incoming edges to a node; thus, sources with a high in-degree collect information from a large range of other sources. Out-degree is the number of outcoming edges from a node. Sources with a high out-degree are often cited; thus, they can communicate outbreak-related information with high visibility. The all-degree is the sum of the in-degree and out-degree. Sources with a high all-degree, also referred to as "hubs", combine the capacity to receive and share outbreak-related information (19).

We further analysed the role of the sources in the different subgraphs (early, late, and non-official), separating the PADI web and HealthMap networks. We classified the sources according to their location in the network (primary versus secondary) and calculated the frequency of each type of source (e.g. online news). We further calculated the proportion of primary and secondary sources according to their geographical focus and specialisation.

Software

The database was constructed using MS Office Access (version 2019). The analysis was performed using the *igraph* package available in R version 3.6 (20).

Results

Event detection

Between 1 July 2018 and 30 June 2019 national animal health authorities reported 351 AI outbreaks in the WOAH. Among these, 81% (284/351) were from domestic birds, 10%

(34/351) were from wild birds, 6% (24/351) were from environmental samples, and 3% (12/351) were unspecified.

The PADI-web detected 408 unique AI outbreak-related news reports, 337 (83%) of which were considered relevant after manual curation (see details in S2 Table). HealthMap detected 163 unique AI outbreak-related news reports, 115 (71%) of which were relevant after manual curation. Among the relevant reports, 37 were detected using both the EBS systems.

Both the PADI-web and HealthMap had a median of one event per news report (min=1, max=14). In the PADI-web relevant news reports, 230 events were described, including 193 events that were not detected by HealthMap (Table 1). Among the detected events, 87% (199/230) were official events; that is, they matched a notified AI outbreak to the WOAH. The remaining 31 events (13%) were unofficial, that is, they could not be verified. The majority (82%) of PADI-web events described AI outbreaks in domestic birds (185/226), while AI outbreaks in wild birds represented 13% (29/226) of the events.

HealthMap relevant reports described 68 events, among which 31 did not overlap with PADI-web detected events (Table 1). Among these events, 88% (60/68) were official and 12% (8/68) were non-official. Similar to the PADI-web, 78% (53/68) of the HealthMap events were in domestic birds, whereas 16% (11/68) were in wild birds.

The non-overlapping events represented 45% (222/489) of all events detected by PADI-web and HealthMap.

Table 1. Number of official and non-official events of AI detected by PADI-web and HealthMap between July 2018 and June 2019. The number of non-overlapping events is shown between parentheses.

	PADI-web		HealthMap	
Type of host	Official	Non-official	Official	Non-official
Domestic birds	174 (147)	15 (13)	48 (23)	5 (3)
Wild birds	16 (10)	13 (12)	9 (3)	2 (1)
Mammals	-	2 (1)	-	1 (0)
Environmental	8 (8)	-	2 (0)	-
Unspecified	1 (1)	1 (1)	1 (1)	-
Total	199 (166)	31 (27)	60 (27)	8 (4)

The Se of HealthMap and PADI-web were 17% (60/351) and 57% (199/351), respectively. The number of events reported to the WOAH and the events detected by the two EBS tools per week and region are provided in the S3 Table.

The timeliness of PADI-web varied from 112 days before to 39 days after notification of an outbreak to the WOAH; 24% (47/199) of the events detected by PADI-web were detected before their official notification, representing 13% of the official events (Fig 1). The PADI-web was timelier in detecting AI events in wild birds than in domestic birds. More precisely, 21% (36/174) of the AI outbreaks in domestic birds in the PADI-web were detected before their official notification, while 56% of the events (9/16) were detected early in wild birds, with a maximum of 112 days before official notification in wild birds.

The timeliness of HealthMap varied from 46 days before to 66 days after an official reporting of an event to the WOAH; 43% (26/60) of the events detected by the tool were reported before the official notification, representing 7% of the official events (Fig 1). In the HealthMap network, 42% (20/48) and 56% (5/9) of AI outbreaks in domestic and wild birds, respectively, were detected before their official notification, with a maximum of 43 days before official notification in wild birds.

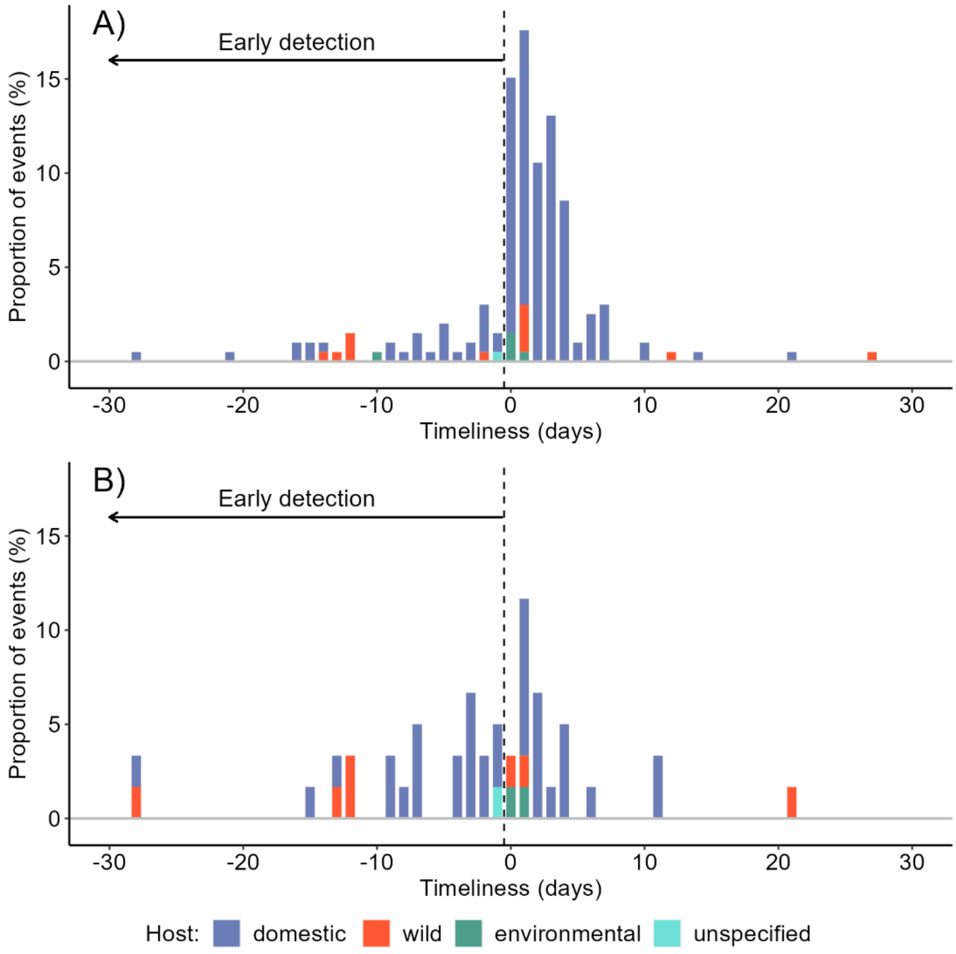

Fig 1. Timeliness in the detection of AI outbreaks according to the type of host for A) PADI-web and B) HealthMap. For visibility, extreme values i.e., less than 30 days and higher than 30 days are not shown.

Network analysis

Network description

*1*During the study period, the PADI-web network disseminated AI outbreak-related information from 250 different nodes (sources), 446 unique edges (links), and 455 paths. The 2HealthMap network comprised 108 nodes, 150 unique edges, and 107 paths. A graphical representation of both networks, as well as details of the edges and nodes, are provided in S4-7 Tables and S1 Fig.

Table 2. Types of sources (i.e., nodes) in PADI-web and HealthMap networks disseminating outbreak-related news on Avian influenza between 1 July 2018 and 30 June 2019

Type of source	PADI-web	HealthMap
online news source	47.6% (n=119)	36.1% (n=39)
national vet authority	14% (n=35)	20.4 % (n=22)
local veterinary authority	13.2% (n=33)	8.3 % (n=9)
local official authority	6% (n=15)	3.7% (n=4)
press agency	4.8% (n=12)	10.2% (n=11)
radio, TV	4.4% (n=11)	3.7% (n=4)
laboratory	2.4% (n=6)	2.8% (n=3)
national official authority	2% (n=5)	5.6% (n=6)
research organisation	1.6% (n=4)	1.9% (n=2)
local person	1.2% (n=3)	0
social platform	1.2% (n=3)	4.6% (n=5)
private company	0.8% (n=2)	0
EBS tool	0.4% (n=1)	1.9% (n=2)
international veterinary authority	0.4% (n=1)	0.9% (n=1)
Total	250	108

Online news was the most represented source (47.6% of the sources in the PADI-web network and 36% in the HealthMap network (Table 2). Local veterinary authorities were more frequent in the PADI web network than in the HealthMap network. Conversely, press agencies represented 10.2% of the HealthMap network sources, compared to 4.8% in the PADI-web network.

Path analysis

Most of the PADI-web paths are composed of two (232/455; 51%) and three (182/455; 40%) edges, 4% (18/455) of the paths are composed of a single edge (they do not cite any source), and 5% (21/455) of the paths are made up of four edges and more. Similarly, most HealthMap paths are composed of two (53/107; 50%) and three (32/107; 30%) edges, 14% (15/107) of the paths are composed of one edge and 5% (7/107) are composed of five edges.

In the PADI-web, 83% (376/455) of the paths propagated events in one day (n=41) or less than one day (n=335). Similar results were observed in HealthMap, with 94% (87/107) of the paths propagating events in one day (n=3) or less than one day (n=84).

Quantitative node analysis

Only 24% (69/287) of the sources in the global network of the PADI-web and HealthMap were characterised by an in-degree greater than 1, indicating that most of the sources received information from a single source. The EBS tools, PADI-web and HealthMap, international veterinary authority, social platforms, press agencies, and research organisations had the highest median in-degrees (Fig 2).

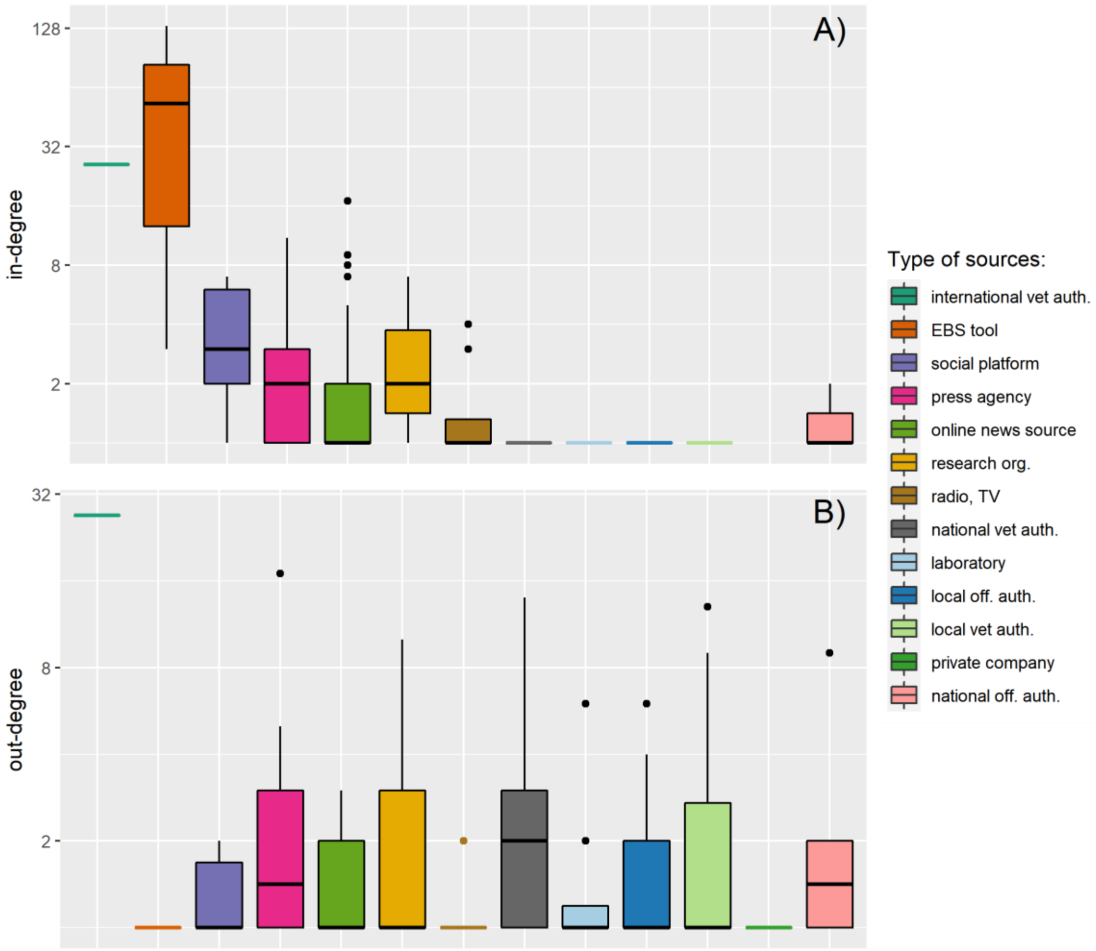

Fig 2. Performance of sources in terms of A) in-degree and B) out-degree, aggregated by type. The y-axis has been log-scaled. Distributions of in-degree and out-degree are represented with box plots based on a 95% confidence interval (outliers are represented with dots).

These groups contain sources which have access to a large amount of information, that is, different sources. The EBS tools had the highest median in-degree because they included

PADI-web and HealthMap, the two aggregators in our study. Except for these two EBS tools, the WOAH stood out with a maximal in-degree equal to 26. Online news sources were characterised by a median in-degree of one, but twelve outliers had an in-degree higher than 5, among which “Times of India”, and two sources specialised in poultry production, “PoultrySite” and “WATTAgNet” (Table 2). Similarly, the social platforms, press agencies, and research organisations were characterised by a high intra-group variance, containing highly connected sources (e.g. Reuters, Xinhua).

The median out-degree of nine out of the 13 types of sources was one, explained by the fact that 64% (183/297) of the sources in the networks were cited only once. Local and national veterinary authorities had higher out-degree values than in-degree values, highlighting their role as sources of information. Individually, the WOAH stands out with the maximal out-degree (27), followed by Reuters, one national authority, and one local veterinary authority (Table 2). As for in-degree, the out-degree variance was high in most groups, owing to the presence of outliers being significantly better transmitters than the other sources of their group.

WOAH was the best-performing source in terms of all degrees, confirming its central position. It was followed by two press agencies, Reuters and Xinhua, the veterinary authority of Bulgaria, and Indian online news, Time of India (Table 2).

Table 2. Top-5 sources in terms of in-degree, out-degree and all-degree. The EBS tools PADI-web and HealthMap were excluded as they were chosen as the aggregators in our study.

	Source	Value	Type
**In-degree**	WOAH	25	International vet auth.
	Times of India	17	Online news
	Xinhua	11	Press agency
	The Poultry Site	9	Online news
	WATTAgNet	8	Online news
**Out-degree**	WOAH	26	International vet auth.
	Reuters	17	Press agency
	Bulgaria Vet Auth	14	National vet auth.
	Minnesota Vet Authorities	13	Local vet auth.
	USA National Oceanic and Atmospheric Administration	10	Research org.
**All-degree**	WOAH	51	International vet auth.
	Reuters	24	Press agency
	Times of India	20	Online news
	Bulgaria Vet Auth	15	National vet auth.
	Xinhua	14	Press agency

Qualitative nodes analysis

National veterinary authorities were the most frequent primary source of events in the late detection of events in both HealthMap and PADI-web (69% and 63% of the primary sources, respectively) and the early detection of HealthMap events (42% of the secondary sources) (Figs 3 and 4; detailed numbers in S8-9 Tables). Local veterinary authorities were the most frequent primary source involved in the early detection of events by the PADI-web (44% of the primary sources) and the second most frequent in HealthMap. The transmission of events in the late detection context was mainly driven by WOAH, press agencies, and online news for both the EBS tools. The transmission of events in the early detection context was mainly driven by online news sources (69% and 58% of the secondary sources in PADI-web and HealthMap, respectively), and press agencies were less frequent than in the early detection networks.

Social platforms represented 13% of the secondary sources involved in the early detection by HealthMap, whereas this type of source was barely used by the PADI-web.

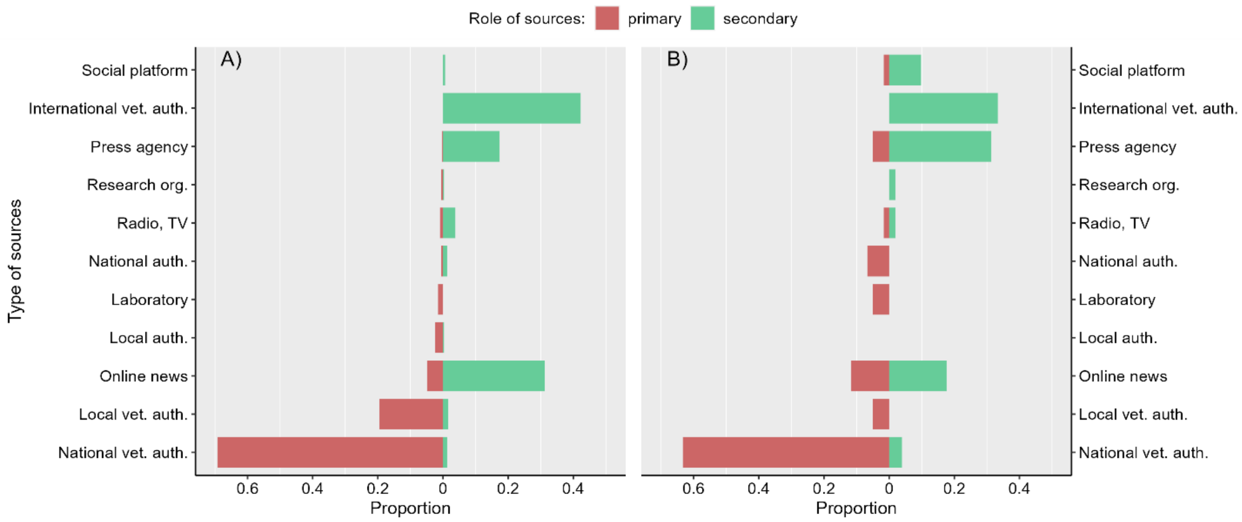

Fig 3. Proportion of the types of primary and secondary sources according to their role in the (a) PADI-web and (b) HealthMap late detection networks. Primary sources are sources that are the first to emit an event, secondary sources are sources which receive and emit an event to another source.

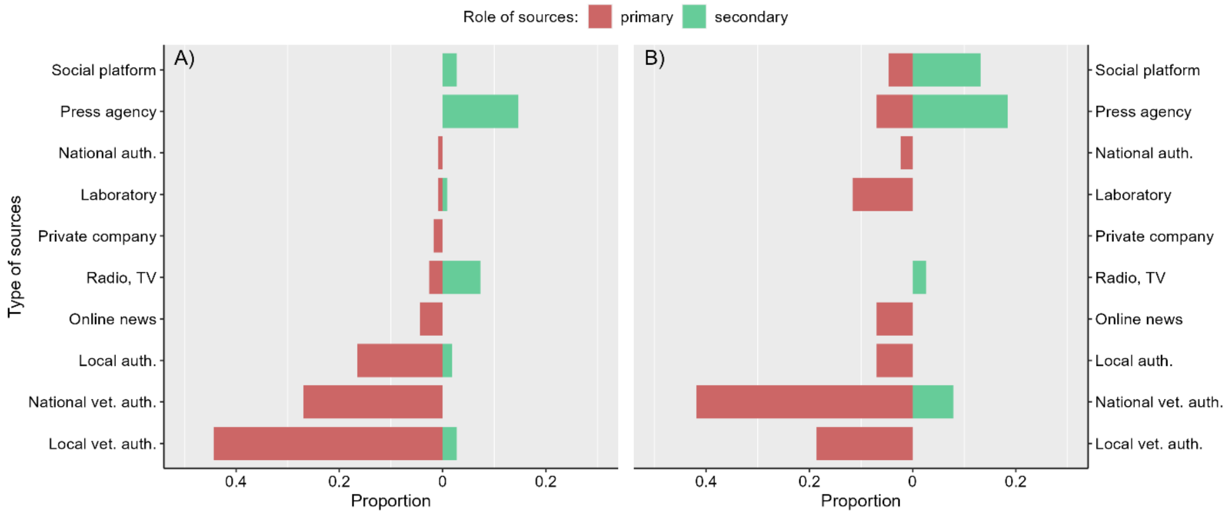

Fig 4. Proportion of the types of primary and secondary sources according to their role in the (a) PADI-web and (b) HealthMap early detection network. Primary sources are sources that are the first to emit an event, secondary sources are sources which receive and emit an event to another source

Nearly 75% of the primary sources in the early detection network of the PADI-web had a local geographical scope, in contrast to 26% in HealthMap (Fig 5). This result was consistent with our previous results, highlighting the role of local sources in the early warning of disease outbreaks. The late detection networks mainly relied on sources with a national scope for both EBS tools, corresponding to the role of the national veterinary authorities.

Early detection networks relied on both national and international sources as intermediates, while late detection was mostly driven by international sources, as explained by the role of the WOAH in the official communication of events in the news.

Specialisation showed the same pattern between late and early detection and between the EBS tools, with at least 75% of the primary sources being specialised (S1 Fig).

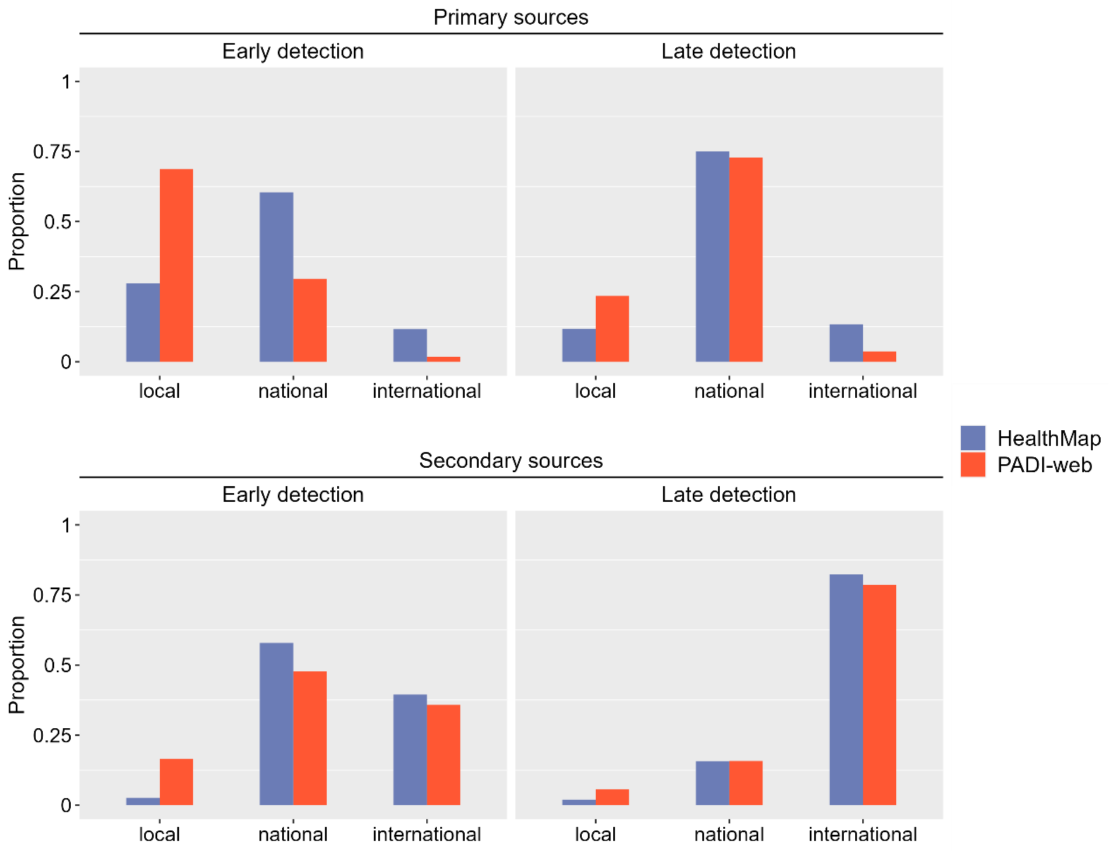

Fig 5. Proportion of the geographic scope of primary and secondary sources in the PADI-web and HealthMap early and late detection networks.

Discussion

In this work, we described how outbreak-related information circulates in news sources captured by two EBS tools, PADI-web and HealthMap. We assessed the EBS tools network, including primary and secondary sources, and their characteristics in terms of type, geographical scope, specialisation, and importance in the dissemination of information using network centrality metrics. In addition, we assessed the timeliness of sharing officialy notified AI outbreak information.

Global performances of PADI-web and HealthMap networks

PADI-web and HealthMap, to varying extents, capture false positive news reports (with respective report precisions of 83% and 71%, respectively). Even if considered irrelevant for this study, most discarded news reports were related to AI events and contained contextual epidemiological information useful for risk assessment purposes, such as protective and control measures or global overviews of AI in a specific region. Both tools are prone to classifying human-related reports as animal-related events. When correctly identified, the detection of zoonotic events in humans is highly relevant from a health perspective. The automatic fine-grained topic classification of news reports still needs improvement to enable discrimination of outbreak declarations from other topics, thus avoiding false alerts and facilitating the triage of sanitary information (21).

The PADI-web was more sensitive than HealthMap. However, the proportion of early detected events compared to the total number of detected events was higher for HealthMap (43% vs. 23%). These differences in captured events may reflect the different web scraping and filtering methods for online news monitoring of the PADI-web and HealthMap. PADI-web is an entirely automatised tool; thus, it captures and filters outbreak-related information without any human intervention. HealthMap is a semi-automatised tool with human moderators that filter news reports that will be shared with users. This may suggest that HealthMap moderators filter and keep only emerging exceptional AI events (such as primary cases), rather than all possible AI events (primary and secondary cases).

Our study highlights the complementarity of these two EBS tools. This complementarity reflects the different sources accessed through the EBS pipelines. Our results showed, for instance, that PADI-web captured more local sources than HealthMap, while the latter relied more heavily on social platforms such as Twitter. Barboza et al. (10) showed that the EBS tool characteristics such as the type of moderation, sources accessed, diseases, languages, and regions covered significantly influence disease detection performance, and that the system's outbreak detection is synergic (complementary). While the proportion of early detected events in our study may seem modest, it is a significant added value to the EBS regarding the reporting of outbreaks of pathogens with zoonotic and pandemic potential. In addition, both networks were highly reactive, mostly propagating information from primary sources to the aggregator in less than one day. Early detection of public health hazards constitutes a fundamental component of efficient outbreak management (22). It may be the main determinant in selecting the appropriate response, thus minimising morbidity and mortality

caused by an infectious disease (23). Event-based surveillance should not be considered a replacement for traditional indicator-based surveillance, but rather, complementary to routinely collected public health surveillance data.

While the reporting of AI events by the EBS tools was highly effective, timely, and reactive, a bottleneck may arise at the step of manual analysis of the detected events. The strength of EBS relies heavily on adequate human resources to feed decision-making chains based on detected events. Therefore, in our future work, we will explore how the detected events can be useful for risk assessment and risk mapping.

Role of the sources

Our results highlight three groups of sources regarding their role in the dissemination of outbreak-related information. EBS tools are aggregators. It is important to note that our results did not reflect ProMED-mail intrinsic performance as an EBS tool, that is, expert network sharing outbreak-related information, but as an intermediate source of HealthMap. Local and national authorities and veterinarians were emitters and were the most important primary sources of events. They produce information that is acknowledged at the local/national level, mostly verified by laboratory tests, and is susceptible to being reported in the media. WOAH, online news, press agencies, social media, and several research organisations combined both abilities by collecting information from a wide range of sources and being highly visible by collector sources in the network (online news, EBS tools). Network performance was driven by the presence of a small number of sources with high individual all-degrees, such as WOAH, Reuters, Xinhua, and several social network platforms. These sources played the role of hubs, not only filtering and disseminating information but also ensuring a connection between different groups in the network (19). The presence of hubs was not the only feature of network performance, as early detection mostly relied on online news sources with individual low all-degrees. Thus, the early components of EBS networks also relied on their ability to monitor a large number of individually low-performant sources.

National online news plays a major role in early detection by disseminating announcements from local and national veterinary authorities, thus making them detectable by EBS tools. Zhang et al. found out that national newspapers (referred to as “local” newspapers in their methods) provided more specific information about the local Zika virus emergence in Brazil than did international newspapers; similar findings were made for outbreak detection in Nepal (12). In a recent study, local sources were more likely to identify a unique event than international sources, indicating that international sources were more likely to be redundant

by publishing multiple reports about the same event (18). This emphasises the need to target local and national sources available on the web, going beyond sources published in English. The monitoring of multi-lingual sources, integrated into the two EBS tools in our work, is a prerequisite for maximising access to national and local media. The retrieval and analysis of non-English texts have been enhanced and facilitated by the improvement of methods for multi-lingual text processing, such as textual classification (25,26) and deep-learning-based translation (27). We believe that efforts to integrate multi-lingual sources will benefit both the Se and timeliness of EBS tools.

Social platforms, mostly used by HealthMap, include generic platforms such as Twitter, but also specialised blogs such as FluTrackers and AvianFluDiary. Specialised blogs are relevant sources for integration into EBS, as they rely on the collection of information from numerous sources, as highlighted by their high median in-degree, previously filtered by domain-specialised moderators. Health blogs were found to cite less sources than online news in a study evaluating H1N1/Swine Flu coverage in the media (28), which is not in line with the highest in-degree found in our study. However, the difference in the number and nature of sources evaluated (eight online news (28)) makes the study hardly comparable. They also translated news from national languages into English, facilitating access to local field information. In addition, owing to their non-official status, online blogs are more prone to communicate events before official notifications. While the classical method of web monitoring is traditionally keyword-oriented (e.g., systematic monitoring of combinations of keywords), source-based monitoring (i.e., systematic monitoring of a specific source) is a costless and easy way to improve existing EBS tools. For instance, retrieving news directly from official government health websites would enhance the geographic representativeness of news aggregators such as Google News (29,30).

It is important to note that our results were specific to the model disease and study period. For example, the Bulgarian veterinary authority appeared to be an important source because 22 outbreaks were observed in Bulgaria during the study period, including a new incursion of the Highly Pathogenic Avian Influenza (HPAI) H5N8 subtype (31) widely reported by Bulgarian media.

Re-thinking the role of event-based surveillance in epidemic intelligence

EBS is sometimes opposed to indicator-based surveillance, as it is based on the use of so-called nonofficial sources. In our study, official veterinary authorities (national or local) represented

80% of primary sources, including those involved in early detection. Thus, the monitoring of the PADI-web and HealthMap was mainly characterised by the detection of national or local official events. This detection includes both the dissemination of WOAH-notified outbreaks (late detection) and the dissemination of official events that have not yet been notified (early detection). In the latter case, EBS tools bypass the international notification procedure and its inherent delays. These findings are consistent with the latest and broader definitions of EBS, stating that media sources collected in the context of EBS can be either official (e.g. a Ministry of Health website) or non-official (e.g. newspaper) (32).

Although the extraction of epidemiological information from collected reports has been widely studied, the automatic extraction of cited sources of events from online sources has not yet received attention. However, based on the findings of our study, we believe that this feature would enhance informal surveillance by enabling the characterisation of an event as official at the international, national, or local level, depending on whether the cited source is the WOAH, a national/local veterinary authority, or non-official, if the type of source does not belong to any of the latest categories. Recent advances in named entity extraction, involving deep learning, combined with a step of normalisation (dictionary or ontology-based), would enable easy identification of the mentioned cited sources. Alerts could be triggered when WOAH is not mentioned. By providing our corpus and databases with open access, we offer the possibility of evaluating and comparing approaches with a high-quality validation dataset.

Both the EBS tools detected several events that could not be found in the EMPRES-i database (S10 Table). These events may have been local AI events that were not communicated at the international level; thus, they did not appear in the EMPRES-i database. They may also correspond to a suspected event that was negated after a negative laboratory test result for the AI virus or to a false alert, as mentioned in a previous study (33). Thus, our study shows that EBS tools can be a source of relevant outbreak information but should be considered complementary to official sources and interpreted with caution. The identification and characterisation of the sources linked in an EBS are important for prioritising the ones regarding truthfulness and reliability. It may be a way of dealing with fake news, for example, by targeting specialised sources. Our study sets the first list of these sources. By extending our approach to emerging zoonotic infectious diseases, the corpora of reliable news sources may be enriched.

Conclusion

Current EBS tools use a diverse, but not identical, network of sources; thus, they can be used in parallel by EI practitioners. In addition, both EBS tools should prioritise specialised media sources and access, when existing, to local and national veterinary authorities' webpages, as they released part of the official event before the international notification to the WOAH. Outbreak-related news travels from a primary source to a final aggregator in one day or less, which is important for early warnings and EI. Both PADI-web and HealthMap shared timely outbreak information on AI in domestic and wild birds, thus contributing to the early detection of EI and as complementary sources to traditional surveillance.

A potential future work could be the integration of the results highlighted in this study to improve EBS systems (for instance, by weighting type of sources in EBS platforms). As mentioned in this paper, we can cite multi-lingual aspects to consider for improving the proposed analysis as well as EBS systems. We could evoke the same type of analysis to conduct with other platforms as well, such as ProMED-mail.

Acknowledgements

We thank the HealthMap project (https://healthmap.org/), which kindly provided us with their data. We acknowledge the reviewer for their constructive comments.

Data reporting

The data used for this study is available at:

https://doi.org/10.5281/zenodo.7324144

Statistical reporting

The code used for the analysis and figures is available at:
https://github.com/SarahVal/EBS-network.

Author Contributions

Sarah Valentin: Conceptualisation, Methodology, Data Curation, Formal Analysis, Validation, Writing – Original Draft Preparation, Writing – Review & Editing
Bahdja Boudoua: Data Curation, Formal Analysis, Writing – Original Draft Preparation, Writing – Review & Editing
Kara Sewalk: Data Curation, Writing – Review & Editing
Nejat Arinik: Visualization, Writing – Review & Editing
Mathieu Roche: Conceptualization, Supervision, Resources, Writing – Review & Editing
Renaud Lancelot: Conceptualization, Supervision, Resources, Writing – Review & Editing

Elena Arsevska: Conceptualization, Methodology, Data Curation, Writing – Original Draft Preparation, Writing – Review & Editing

Supporting information

S1 Table. Definitions used to characterize the types of sources, specialization and geographical focus in PADI-web and HealthMap networks.

S2 Table. Summary of the manual curation of the relevance of PADI-web and HealthMap reports.

S3 Table. The number of events reported to the WOAH and detected by the two EBS tools per week (mean, min, and max) and per region.

S1 Fig. PADI-web (A) and Healthmap (B) networks. Sources were grouped by type. The edge colour corresponds to the colour of the incoming source type, thus enabling the visualisation of the direction of information dissemination, that is, orange edges represent incoming edges to an EBS tool.

S4 Table. Legend of the node's names in the PADI-web network.

S5 Table. Legend of the node's names in the HealthMap network.

S6 Table. PADI-web network composition.

S7 Table. HealthMap network composition.

S8 Table. Proportion of the types of sources according to their role in the (a) PADI-web and (b) HealthMap late detection networks.

S9 Table. Proportion of the types of sources according to their role in the (a) PADI-web and (b) HealthMap early detection networks.

S2 Fig. Type of specialization of primary and secondary sources for the detection of early and late events in PADI-web and HealthMap networks

S10 Table. Type of primary and secondary sources involved in the detection and transmission of non-official events in PADI-web and HealthMap networks.

References

1. Paquet C, Coulombier D, Kaiser R, Ciotti M. Epidemic intelligence: a new framework for strengthening disease surveillance in Europe. Eurosurveillance. 1 déc 2006;11(12):5‑6.

2. Wilburn J, O’Connor C, Walsh AL, Morgan D. Identifying potential emerging threats through epidemic intelligence activities—looking for the needle in the haystack? International Journal of Infectious Diseases. 1 déc 2019;89:146‑53.

3. Kaiser R, Coulombier D, Baldari M, Morgan D, Paquet C. What is epidemic intelligence, and how is it being improved in Europe? Weekly releases (1997–2007). 2 févr 2006;11(5):2892.

4. Freifeld CC, Mandl KD, Reis BY, Brownstein JS. HealthMap: Global Infectious Disease Monitoring through Automated Classification and Visualization of Internet Media Reports. Journal of the American Medical Informatics Association. 1 mars 2008;15(2):150‑7.

5. Valentin S, Arsevska E, Falala S, de Goër J, Lancelot R, Mercier A, et al. PADI-web: A multilingual event-based surveillance system for monitoring animal infectious diseases. Computers and Electronics in Agriculture. 1 févr 2020;169:105163.

6. Bhatia S, Lassmann B, Cohn E, Desai AN, Carrion M, Kraemer MUG, et al. Using digital surveillance tools for near real-time mapping of the risk of infectious disease spread. npj Digital Medicine. 16 avr 2021;4(1):1‑10.

7. Arsevska E, Valentin S, Rabatel J, Hervé J de G de, Falala S, Lancelot R, et al. Web monitoring of emerging animal infectious diseases integrated in the French Animal Health Epidemic Intelligence System. PLOS ONE. août 2018;13(8):e0199960.

8. Valentin S, Mercier A, Lancelot R, Roche M, Arsevska E. Monitoring online media reports for early detection of unknown diseases: insight from a retrospective study of COVID-19 emergence. Transboundary and Emerging Diseases [Internet]. 19 juill 2020 [cité 28 juill 2020]; Disponible sur: https://onlinelibrary.wiley.com/doi/abs/10.1111/tbed.13738

9. Plateforme ESA [Internet]. [cité 1 nov 2022]. Disponible sur: https://plateforme-esa.fr/fr

10. Barboza P, Vaillant L, Le Strat Y, Hartley DM, Nelson NP, Mawudeku A, et al. Factors influencing performance of internet-based biosurveillance systems used in epidemic intelligence for early detection of infectious diseases outbreaks. PLoS ONE. 5 mars 2014;9(3):e90536.

11. Barboza P, Vaillant L, Mawudeku A, Nelson NP, Hartley DM, Madoff LC, et al. Evaluation of epidemic intelligence systems integrated in the Early Alerting and Reporting project for the detection of A/H5N1 influenza events. Nishiura H, éditeur. PLoS ONE. 5 mars 2013;8(3):e57252.

12. Schwind JS, Norman SA, Karmacharya D, Wolking DJ, Dixit SM, Rajbhandari RM, et al. Online surveillance of media health event reporting in Nepal: digital disease detection from a One Health perspective. BMC International Health and Human Rights. 21 sept 2017;17(1):26.

13. Xu Y, Gong P, Wielstra B, Si Y. Southward autumn migration of waterfowl facilitates cross-continental transmission of the highly pathogenic avian influenza H5N1 virus. Sci Rep. 10 août 2016;6(1):30262.

14. Mostafa A, Abdelwhab EM, Mettenleiter TC, Pleschka S. Zoonotic Potential of Influenza A Viruses: A Comprehensive Overview. Viruses. 13 sept 2018;10(9):497.

15. Welte VR, Terán MV. Emergency Prevention System (EMPRES) for Transboundary Animal and Plant Pests and Diseases. The EMPRES-Livestock: An FAO Initiative. Annals of the New York Academy of Sciences. 2004;1026(1):19‑31.

16. Farnsworth ML, Hamilton-West C, Fitchett S, Newman SH, de La Rocque S, De Simone L, et al. Comparing national and global data collection systems for reporting, outbreaks of H5N1 HPAI. Preventive Veterinary Medicine. 1 juill 2010;95(3):175‑85.

17. Kolaczyk ED. Statistical Analysis of Network Data: Methods and Models. 1st éd. Springer Publishing Company, Incorporated; 2009.

18. Kolaczyk ED. Descriptive Analysis of Network Graph Characteristics. In: Kolaczyk ED, éditeur. Statistical Analysis of Network Data: Methods and Models. New York, NY: Springer; 2009. p. 1‑44. (Springer Series in Statistics).

19. Weber MS, Monge P. The Flow of Digital News in a Network of Sources, Authorities, and Hubs. Journal of Communication. déc 2011;61(6):1062‑81.

20. Csardi G, Nepusz T. The igraph software package for complex network research. InterJournal. 2006;Complex Systems:1695.

21. Valentin S, Arsevska E, Vilain A, De Waele V, Lancelot R, Roche M. Elaboration of a new framework for fine-grained epidemiological annotation. Scientific Data. 26 oct 2022;9(1):655.

22. Adini B, Singer SR, Ringel R, Dickmann P. Earlier detection of public health risks – Health policy lessons for better compliance with the International Health Regulations (IHR 2005): Insights from low-, mid- and high-income countries. Health Policy. oct 2019;123(10):941‑6.

23. Yan SJ, Chughtai AA, Macintyre CR. Utility and potential of rapid epidemic intelligence from internet-based sources. International Journal of Infectious Diseases. 1 oct 2017;63:77‑87.

24. Yoon S, Odlum M, Broadwell P, Davis N, Cho H, Deng N, et al. Application of Social Network Analysis of COVID-19 Related Tweets Mentioning Cannabis and Opioids to Gain Insights for Drug Abuse Research. Stud Health Technol Inform. 26 juin 2020;272:5‑8.

25. Mutuvi S, Boros E, Doucet A, Jatowt A, Lejeune G, Odeo M. Multilingual Epidemiological Text Classification: A Comparative Study. In: Proceedings of the 28th International Conference on Computational Linguistics [Internet]. Barcelona, Spain (Online): International Committee on Computational Linguistics; 2020. p. 6172‑83. Disponible sur: https://aclanthology.org/2020.coling-main.543

26. Névéol A, Dalianis H, Velupillai S, Savova G, Zweigenbaum P. Clinical Natural Language Processing in languages other than English: opportunities and challenges. Journal of Biomedical Semantics. 30 mars 2018;9(1):12.

27. Singh SP, Kumar A, Darbari H, Singh L, Rastogi A, Jain S. Machine translation using deep learning: An overview. In: 2017 International Conference on Computer, Communications and Electronics (Comptelix). 2017. p. 162‑7.

28. Gao F, Zhang M, Sadri S. Newspapers Use More Sources Compared to Health Blogs in H1N1/Swine Flu Coverage. Newspaper Research Journal. 1 mars 2011;32(2):89‑96.

29. Feldman J, Thomas-Bachli A, Forsyth J, Patel ZH, Khan K. Development of a global infectious disease activity database using natural language processing, machine learning, and human expertise. J Am Med Inform Assoc. 30 juill 2019;26(11):1355‑9.

30. Zhang Y, Ibaraki M, Schwartz FW. Disease surveillance using online news: Dengue and zika in tropical countries. J Biomed Inform. févr 2020;102:103374.

31. Zecchin B, Goujgoulova G, Monne I, Salviato A, Schivo A, Slavcheva I, et al. Evolutionary Dynamics of H5 Highly Pathogenic Avian Influenza Viruses (Clade 2.3.4.4B) Circulating in Bulgaria in 2019–2021. Viruses. oct 2021;13(10):2086.

32. Tu W, Jin L, Ni D. Early Warning Practice Using Internet-Based Data. Early Warning for Infectious Disease Outbreak. 2017;231‑41.

33. Barboza P. Evaluation of epidemiological intelligence systems applied to the early detection of infectious diseases worldwide. 2015;122.

Click here to access/download;Figure;Fig1.tiff

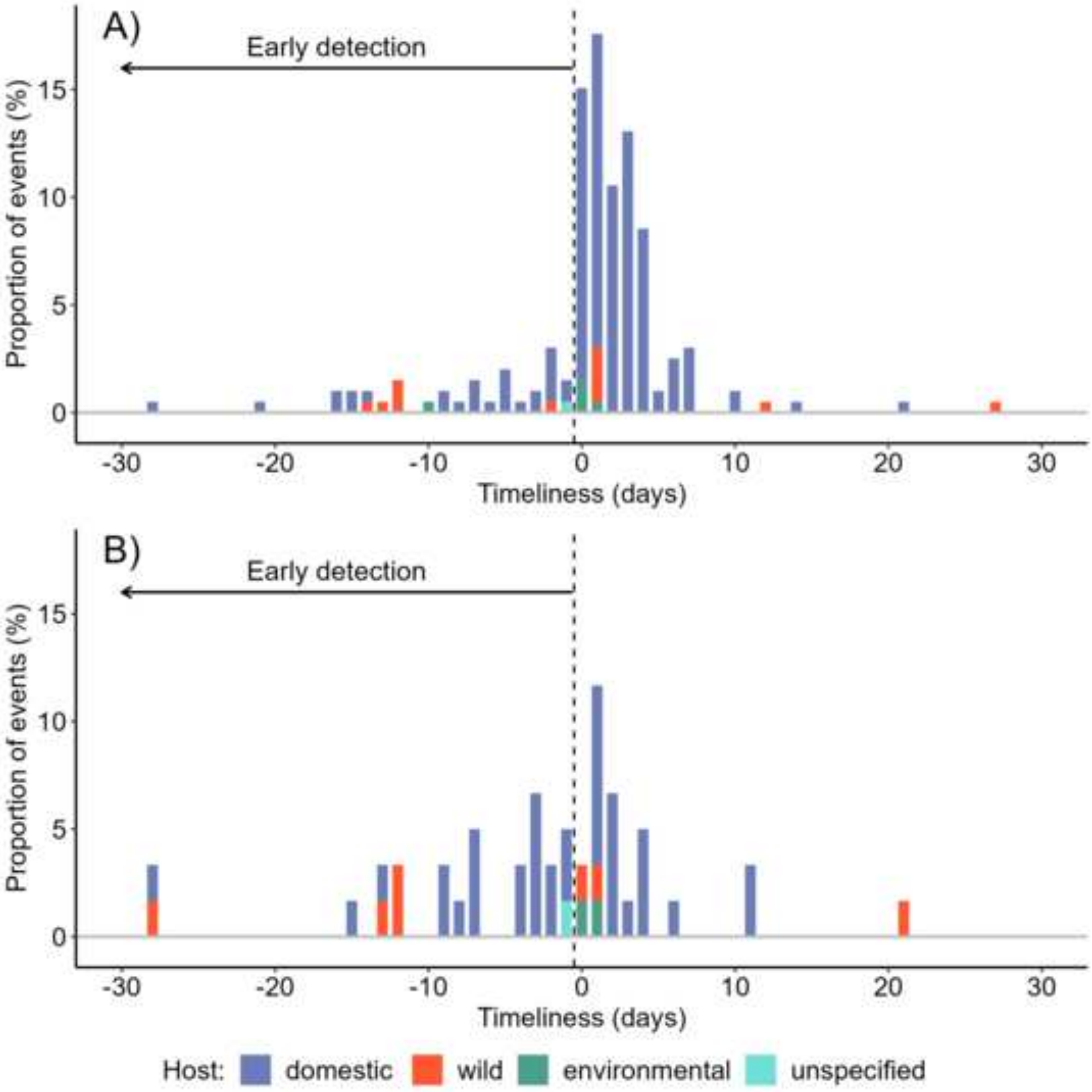

Fig2

Click here to access/download;Figure;Fig2.tiff

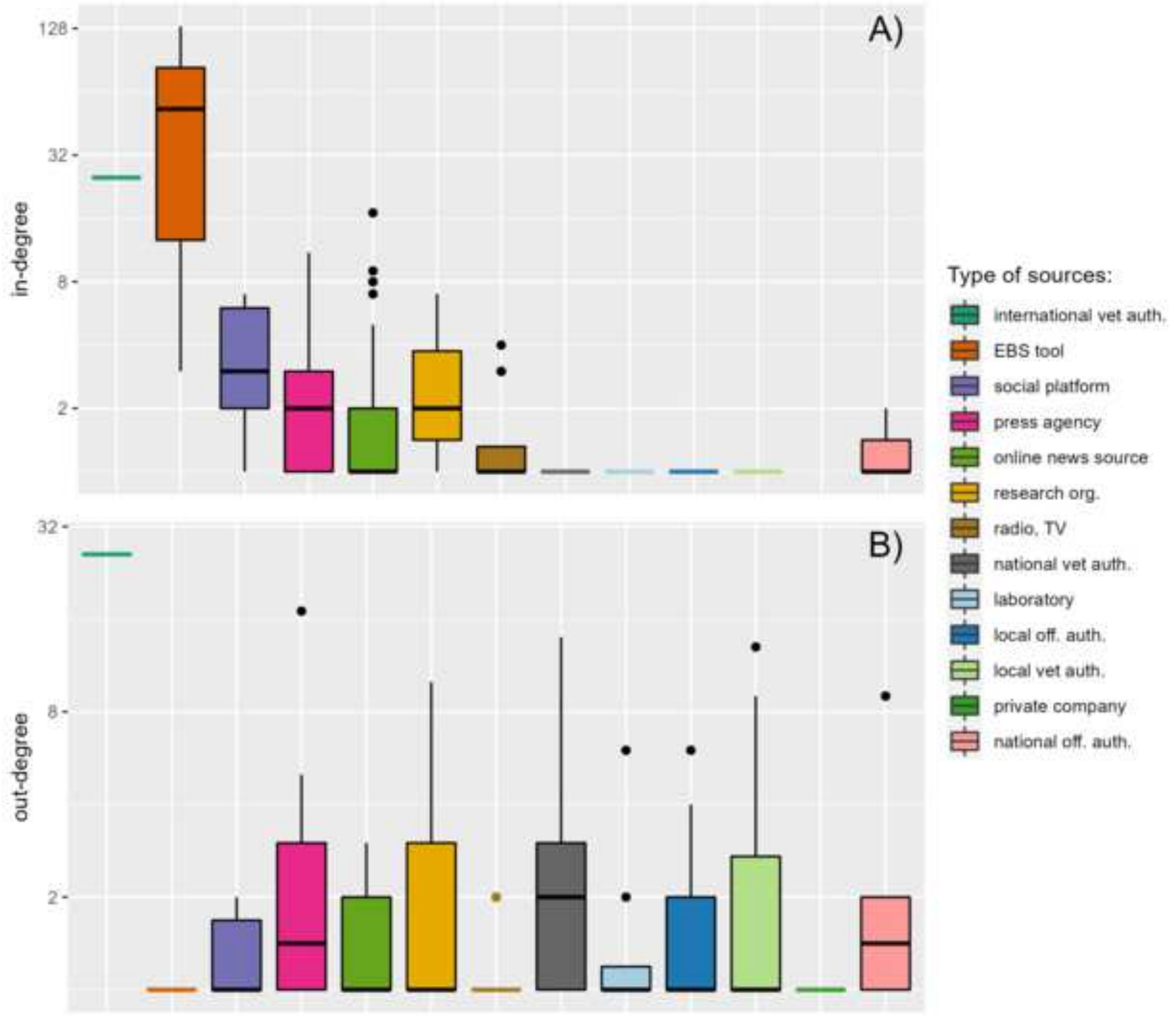

Fig3

Click here to access/download;Figure;Fig3.tiff

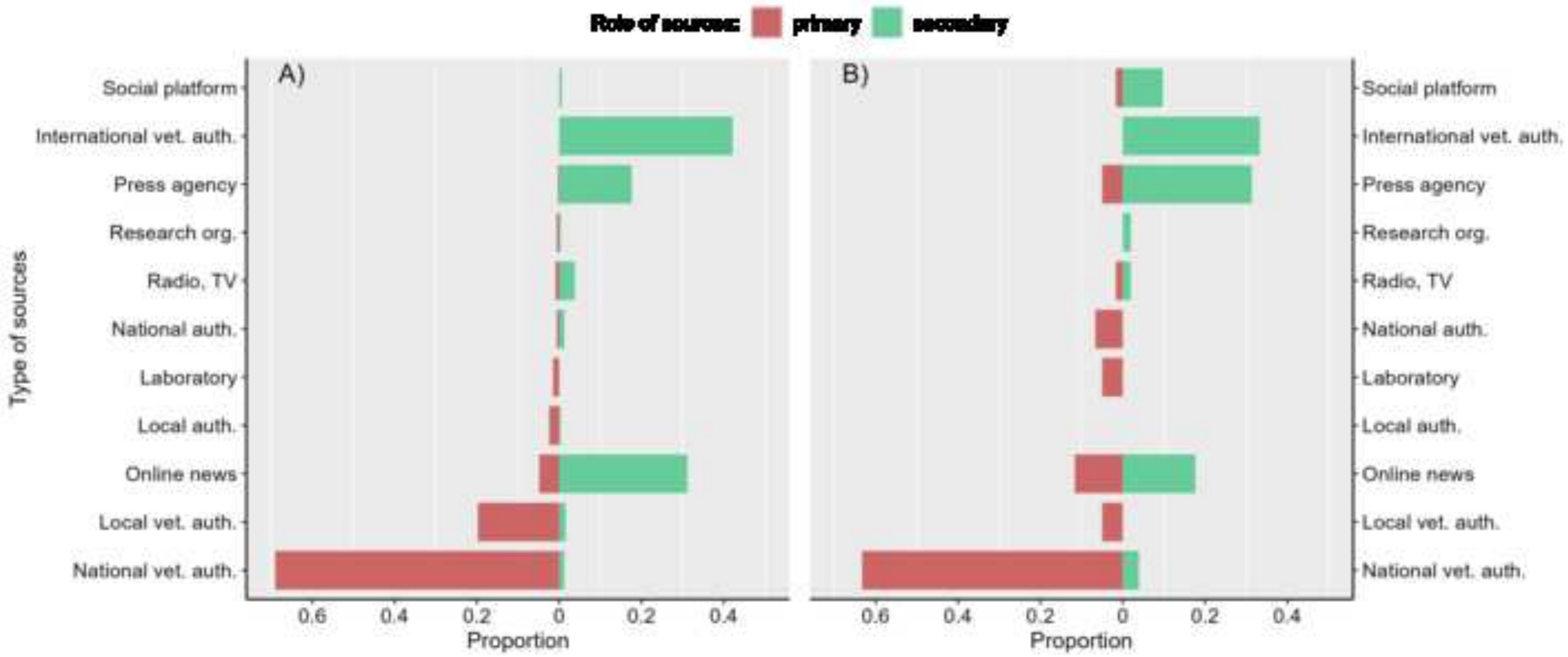

Fig4

Click here to access/download;Figure;Fig4.tiff

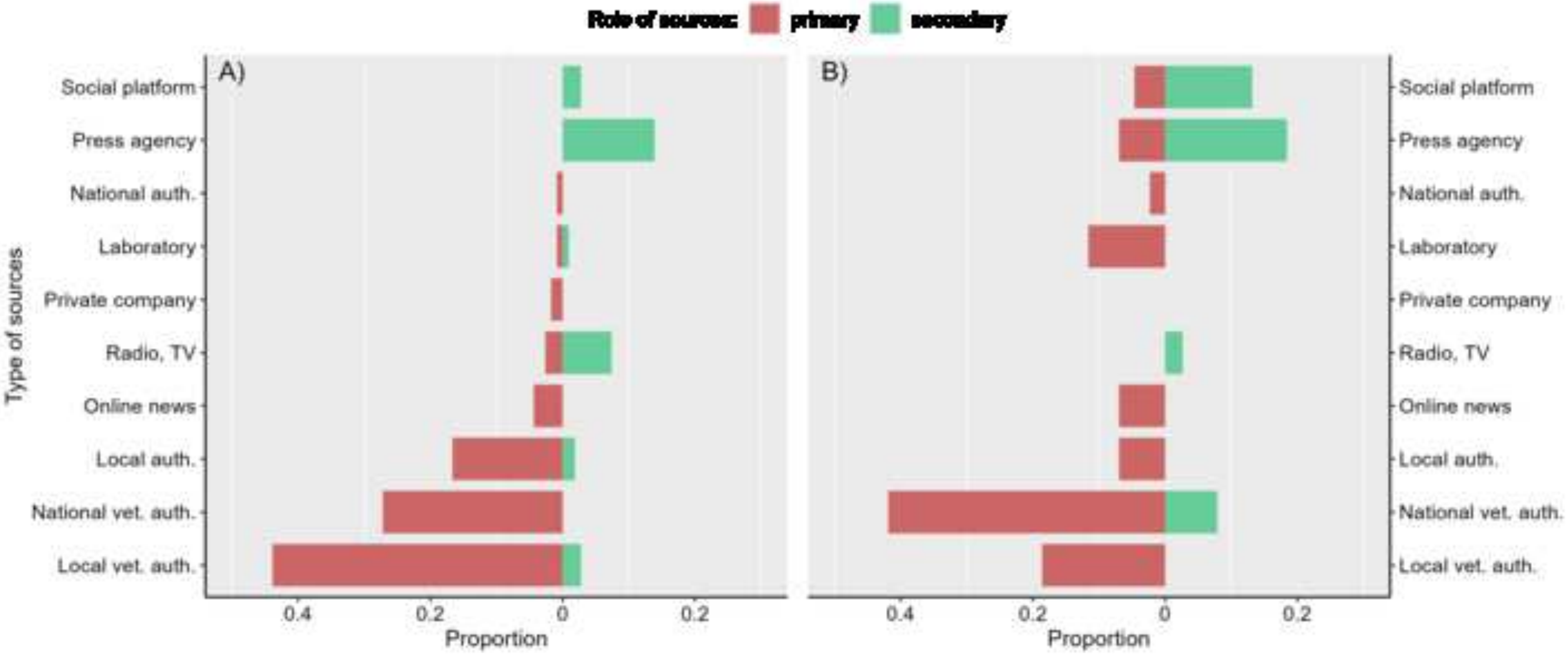

Fig5

Click here to access/download;Figure;Fig5.tiff

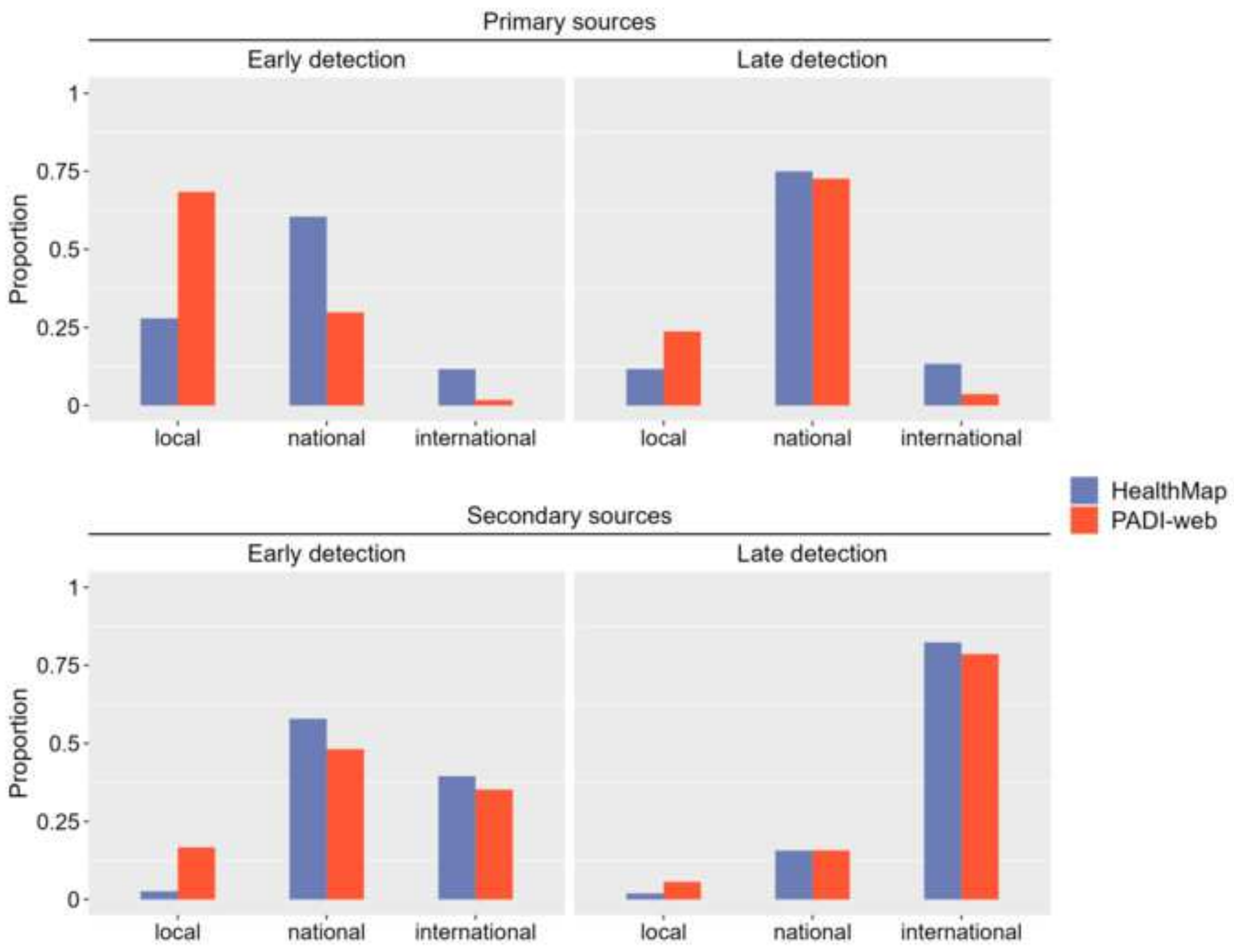

Supplementary material

Click here to access/download
Supporting Information - Compressed/ZIP File Archive
Supplementary material.zip

Dissemination of information in event-based surveillance, a case study of Avian Influenza

Sarah Valentin[1,2,3,4¶], Bahdja Boudoua[1,2¶], Kara Sewalk[5], Nejat Arınık[2], Mathieu Roche[1,2,3], Renaud Lancelot[1,3], Elena Arsevska[1,3*]

[1] Joint Research Unit Animal, Health, Territories, Risks, Ecosystems (UMR ASTRE), French Agricultural Research Centre for International Development (CIRAD), National Research Institute for Agriculture, Food and Environment (INRAE), Montpellier, France

[2] Joint Research Unit Land, Environment, Remote Sensing and Spatial Information (UMR TETIS), Université de Montpellier, AgroParisTech, French Agricultural Research Centre for International Development (CIRAD), French National Centre for Scientific Research (CNRS), National Research Institute for Agriculture, Food and Environment (INRAE), Montpellier, France

[3] French Agricultural Research Centre for International Development (CIRAD), Montpellier, France

Commented [EA1]: Full name provided for all affiliations as according to PlosOne guidelines

[4] Département de biologie, Université de Sherbrooke, Sherbrooke, Canada

[5] Computational Epidemiology Group, Boston Children's Hospital, Boston, United States

[¶] These authors contributed equally to this work

*Corresponding author
E-mail: elena.arsevska@cirad.fr (EA)

ORCID affiliations

Sarah Valentin https://orcid.org/0000-0002-9028-681X

Bahdja Boudoua https://orcid.org/0000-0001-6174-3252

Kara Sewalk https://orcid.org/0000-0002-2917-0869

Nejat Arınık https://orcid.org/0000-0001-5080-4320

Mathieu Roche https://orcid.org/0000-0003-3272-8568

Renaud Lancelot https://orcid.org/0000-0002-5826-5242

Elena Arsevska https://orcid.org/0000-0002-6693-2316

Abstract

Event-Based Surveillance (EBS) tools, such as HealthMap and PADI-web, monitor online news reports and other unofficial sources, with the primary aim to provide timely information to users from health agencies on disease outbreaks occurring worldwide.

In this work, we describe how outbreak-related information disseminates from a primary source, via a secondary source, to~~until~~ a definitive aggregator, an EBS tool, during the 2018/19 avian influenza season. We analysed 337 news items from the PADI-web and 115 news articles from HealthMap EBS tools~~,~~ reporting avian influenza outbreaks in birds worldwide between July 2018 and June 2019. We used the sources cited in the news to trace the path of each outbreak. We ~~have~~ built a directed network~~,~~ with nodes representing the sources (characteris~~z~~ed by type, specialis~~z~~ation, and geographical focus) and edges representing the flow of information. We calculated the degree as a centrality measure to determine the importance of the nodes in information dissemination. We analysed the role of the sources in early detection (detection of an event before its official notification) to the World Organisation for Animal Health (WOAH) and late detection.

A total of 23% and 43% of the avian influenza outbreaks detected by the PADI-web and HealthMap, respectively, were shared ~~in a timely manner~~on time~~,~~ before their notification. For~~In~~ both tools, national and local veterinary authorities were the ~~major~~ primary sources of early detection. The early detection component mainly relied on the dissemination of nationally ~~-~~acknowledged events by online news and press agencies, by~~-~~passing international reporting to the WAOH. ~~The~~ WOAH was the major secondary source for late detection, occupying a central position between national authorities and disseminator sources, such as online news. PADI-web and HealthMap were highly complementary in terms of detected sources, explaining why~~that~~ 90% of the events were detected by only one of ~~bo~~the tools.

We show that current EBS tools can ~~timely~~ provide timely ~~complete~~ outbreak-related information and ~~we provide~~ priority news sources to improve digital disease surveillance.

Keywords: event-based surveillance, digital disease detection, network analysis, avian influenza

Introduction

Commented [EA2]: The introduction has been reworked to account for reviewers comments and suggestions, including the use of EBS tools for health agencies, examples of users, and a more simple description of the EBS process in order to be understood by a larger audience of readers

Recent developments in i~~I~~nternet and digital technologies have contributed to the establishment~~set-up~~ of the Epidemic Intelligence (EI) framework, aiming at the early identification of potential health threats from sources of intelligence of any nature, their verification, and assessment for timely prevention and control by public and animal health (PH/AH) agencies. Event-based surveillance (EBS), as part of the EI, gathers unstructured data on potential and non-verified disease outbreaks mainly by monitoring the web, such as online media, social networks, and blogs. The EBS is complementary to ~~the~~ traditional, indicator-based surveillance (IBS), also part of the EI, which collects structured data on verified disease outbreaks through routine national surveillance systems (1–3).

Since the early 2000s, several automatis~~z~~ed EBS tools with open-access have been created, such as HealthMap, operating since 2006 and monitoring web sources for the public, animal, and plant health threats (4),~~;~~ and PADI-web, operating since 2016 and monitoring web sources for mainly animal health threats (5). The two open-access tools are used for the detection and monitoring of potential outbreaks reported in non-official sources on the web, including known diseases, such as avian influenza or Ebola (6,7), or clinical signs of unknown origin, such as acute respiratory syndrome (8). The main users of the two tools are EI staff at national and supranational PH/AH agencies and organizations, among others such as the French Platform for epidemiological surveillance in animal health (Platform ESA) (7) and the European Centre for Disease Control (ECDC) (9).

Both HealthMap and PADI-web implement algorithms to capture news on potential disease outbreaks from a broad range of data sources on the web~~,~~ in multiple languages and geographical regions (4,5). For example, HealthMap gathers data from Baidu, SoSo, Google News aggregators, and ProMED-mail in nine languages. PADI-web collects data from the Google News aggregator in 16~~sixteen~~ languages. Both tools~~,~~ further implement classification and information extraction algorithms to filter and extract the relevant outbreak information in a structured format ~~relevant outbreak information~~ from the free text, such as the place, date, and host of a described outbreak. Finally, HealthMap provides users with a world map interface to visualis~~z~~e the reports and information sources that report outbreaks. PADI-web ~~rather~~ provides users with a list of ~~the~~ information sources and ~~the~~ news content that reports~~ing~~ outbreaks.

Previous evaluations of the EBS tools in use today, including HealthMap and PADI-web, focused mainly on the assessment of their extrinsic performance, such as timeliness, positive

predictive value, or sensitivity (Se) in detecting outbreaks from the sources they monitor, compared to ~~the~~ official disease outbreaks (6,7). From an end-user perspective~~point of view~~, Barboza et al. (10,11) assessed metrics such as the usefulness, simplicity, and flexibility of an EBS tool.

The understanding of the role of the inputs (i.e. the monitored sources)~~, i.e., the monitored sources,~~ on the performance~~s~~ of ~~the~~ EBS tools is less explored.~~-~~ Barboza et al., 2014 (10) found that the type of moderation, sources, languages, regions of occurrence, and types of cases influence ~~an~~ EBS tool performance. Schwind et al. (2017)~~, 2017~~ (12) ~~found~~ identified that ~~the~~ domestic and~~,~~ national news sources were more likely to report outbreaks than international news portals~~portal sources~~.

This study aimed~~s~~ to fill~~at filling~~ the existing gap in the role of ~~the~~ sources monitored by ~~the~~ EBS tools. We consider ~~the~~ EBS tools as aggregators which collect disease outbreak information at the end of a transmission chain, referred to as a network. More precisely, we aimed to ~~assessat assessing where~~characterise ~~does~~ the sources of outbreak information detected by ~~the~~ an EBS tool ~~comes from,~~ and assess how the sanitary information ~~it~~ circulates through the monitored sources before being detected by an EBS tool.

We assessed the flow of outbreak information from primary sources, providers of the information, until the end ~~-~~sources, ~~the~~ EBS tools, and final aggregators of the information. We represent this~~these~~ information flow~~s~~ through a network structure. Moreover, we provide an in-depth analysis of the extracted networks and the characteristics of the sources involved in outbreak reporting using~~by~~ two EBS tools, HealthMap and PADI-web. In~~More precisely, in~~ this study,~~paper~~ we address three main questions:

1. What are the sources involved in the reporting of outbreak-related information on the web?

2. What are~~is~~ the roles of the different sources regarding the dissemination of outbreak-related information on the web, and what are their characteristics in terms of type, specialisation, and geographical scope?

3. How complementary are the different EBS tools in terms of monitored sources and reported outbreak-related information?

In this study, we further propose a new representation of the sources and their networks involved in digital disease surveillance~~,~~ to improve the detection and analysis of signals of disease emergence from online media. This representation and associated analysis ~~enable to~~ addres~~s~~ses these~~the abovementioned~~ questions.

The remainder of this~~This~~ paper is organised as follows. First, we summarisz~~z~~e the objectives and methods of assessing~~to assess the~~ information dissemination across ~~the~~ data (news) sources. Next, we detail our methodology to collect and assess the dissemination of outbreak-related information via PADI-web and HealthMap. We present and discuss our results in S~~s~~ection 3, before summarising the main conclusions of our work.

Materials and methods

Data collection

To conduct this study, we chose to analyse news reports of Avian Influenza (AI) detected by two EBS tools, PADI-web and HealthMap. ~~The~~ AI viruses can spread over long distances via trade in poultry and wild-caught birds, ~~but~~ as well~~also~~ as via the movement~~s~~ of wild birds (13). ~~The~~ AI outbreaks are responsible for~~of~~ significant economic losses resulting from trade restrictions, loss ~~of the free~~ of disease-free status for ~~the~~ affected countries, or culling measures in infected flocks. Moreover, AI has ~~a~~ great zoonotic potential, as some subtypes can infect different avian and mammalian animal hosts, including humans (14). Thus, ~~the~~ early detection of AI outbreaks is essential for implementing protection and control measures and helping contain their spread.

For our study, we extracted all English news reports from PADI-web and HealthMap EBS tools, which described one or several AI outbreaks and were published between 1 July 2018 and 30 June 2019 (i.e.~~,~~ 337 news reports from PADI-web and 115 news reports from HealthMap). We chose a one-year study period (July 2018 to~~-~~ June 2019) to capture the spatiotemporal~~space-time~~ epidemiological characteristics of ~~the~~ AI outbreaks ~~around the~~ worldwide. The detection of the virus at a specific date and time is hereafter referred to as an event (most ~~of~~ events are outbreaks, but some ~~of them~~ describe the detection of the virus in the environment). Two epidemiologists (BB, SV, authors of this work) manually assessed the relevance of each news item (a report was~~being~~ considered ~~as~~ relevant if it containeds~~s~~ at least one event) and discarded ~~the~~ irrelevant news. Importantly, the events can be either reported as confirmed or suspected, as one of the keystones of EI~~pidemic Intelligence~~ is the detection of potential outbreaks before ~~their~~ official confirmation.

Event detection

Two epidemiologists (BB and~~,~~ SV, authors of this work) read the ~~retained~~ relevant news and identified all ~~the~~ reported events. E~~For e~~ach event described in thea~~a~~ detected news was~~, we~~ classified ~~it~~ as official or non-official.

Official events corresponded to outbreaks officially notified~~ outbreaks~~ by AH authorities. For this purpose, we used the~~ ~~ Emergency Prevention System for Priority Animal and Plant Pests and Diseases (EMPRES-i), a global animal health information system (15,16) developed by the Food and Agriculture Organization (FAO) of the United Nations. EMPRES-i allows free access to and sharing~~e~~ of disease outbreak data to support data analysis and notification to national AH authorities by monitoring and summaris~~z~~ing the global status of priority animal diseases and zoonoses, including AI. One of the main sources of information for the EMPRES-~~I ~~i is the verified disease outbreak data~~,~~ provided by national AH authorities, mainly through traditional disease surveillance by~~to~~ the World Organisation for Animal Health (WOAH). The EMPRES-i has ~~been ~~tracked~~ing~~ AI outbreaks since 2003.

When an event could not be linked to an official event from the EMPRES-i, we labelled it as non-official and recorded the epidemiological information provided in the report (i.e.~~,~~ ~~serotype~~subtype, reported date of the event, the country and location of the event, the host affected, and the number of cases). This enabled us to identify when the~~a~~ same non-official event was reported in~~by~~ different news articles.

For both official and non-official events, we calculated the number of non-overlapping events between the two EBS tools, that is~~i.e.~~, the events that were detected by one tool out of two.

For the official events, we evaluated the Se~~sensitivity~~ and ~~the ~~timeliness of each tool. Timeliness is the lag in days between the date of official notification to the WOAH (day 0), as recorded in the EMPRES-i database, and the date when the same event was first detected by the PADI-web and HealthMap. A negative lag means that the EBS tool ~~timely ~~detects~~ed~~ an event in a timely manner, that is~~i.e.~~, before the date of notification. A positive lag indicated that the EBS tool was untimely for detecting an outbreak, that is~~i.e.~~, the same day or after the official notification date. ~~Sensitivity (~~Se~~)~~ is defined as the ability of the EBS tool to report an event present in the EMPRES-i database, corresponding to the proportion of true positive events (TP) among the sum of true positive and false~~-~~negative (FN) events (Se=TP/(TP+FN)). A ~~true positive (~~TP~~)~~ event was defined as all AI outbreaks in~~from~~ the EMPRES-i database during the study period. An ~~false negative (~~FN~~)~~ event was defined as an event present in the EMPRES-i database that was~~but~~ not detected by an EBS tool. The specificity of event-based surveillance tools cannot be calculated, as it is impossible to assess the status of non-official events~~ they~~ detected (11); there may be false positive events~~,~~ as well as TP ~~true positive ~~events not reported to the gold standard databases (WOAH and EMPRES-i). -

Commented [EA3]: The gold-standard dataset is better described as required by reviewers

Network construction

To~~In order to~~ trace back ~~from the end to~~ the primary sources, we manually traced the information pathways of all events mentioned in the PADI-web and HealthMap news. We assumed that an information pathway could be deduc~~t~~ed from the sources cited in the~~a~~ news content. In the~~an~~ information pathway, the first node is called the primary source (i.e.~~,~~ the earliest emitter source), the last node is called the final source (i.e.~~,~~ the final aggregator, PADI-web, or HealthMap), and the remaining nodes, if any, are called secondary sources. The combination of all ~~the~~ information pathways from ~~the~~ news events gives a network structure ~~in the end~~, referred to as a network of information pathways.

Let $G = (V, E, A)$ be a directed unweighted attributed graph ~~to~~ representing a network of information pathways, where V, E, and A ~~is~~ are the set of network nodes,~~, E is the set of~~ network edges, and~~, and A is the set of~~ attributes associated with the nodes, respectively (17). The network nodes represent the sources and ~~the~~ final aggregators (PADI-w~~W~~~~w~~eb and HealthMap). Each node has three attributes, as defined in S1 Table: type (e.g.~~,~~ online news source, national veterinary authority, etc.), geographical focus (local, national, or international), and specialis~~z~~ation in ~~the~~ animal health news coverage (general or specialis~~z~~ed). The edges represent the dissemination of event information between two nodes (an emitter source, S_E~~,~~ that~~which~~ sends the event, and a receptor source, S_R~~,~~ that~~which~~ receives the event). The graph is directed~~,~~ as the information is transmitted from the~~an~~ ~~emitter source~~ S_E to the ~~receptor sources,~~ S_R. A directed graph is formally defined as a graph G for which each edge in E has an ordering to its vertices (i.e.~~,~~ such~~o~~ that $e_1 = (u,v)$ is distinct from $e_2 = (v,u)$, for $e_1,e_2 \in E$). In our approach, the edges are not weighed~~,~~ because we create an edge between an~~ emitter~~ S_E and ~~receptor sources~~ S_R if S_R cites~~d~~ S_E at least once~~one time~~.

It is worth noti~~ci~~ng that an event can be transmitted through several paths~~,~~ and that a path can transmit several events. The first case occurs~~happens~~ when the same event is reported by different sources (e.g.~~, reported into~~ two online news articles). The second ~~case~~ occurs when a single news article reports several events. Based on this fact, we ~~could~~ separated the global graph into three subgraphs depending on the type of events detected and their timeliness: a~~the~~ graph containing the paths associated with the early detection of official events (timeliness < 0), a~~the~~ graph containing the paths associated with the late detection of official events (timeliness ~~>~~≥~~=~~ 0), and a~~the~~ graph containing the paths associated with the detection of non-official events.

Network analysis

Network description

We first describedd the networkss of information pathways extracted from the PADI-web and HealthMap news, PADI-web, and HealthMap networks hereafter, in terms of the number of edges, nodes, and paths. We visualiszed the networks usingwith a chord diagram and, classifiedclassifying the nodes according to their source types.

Path analysis

To evaluate the network performancess regarding the dissemination of health events, we calculated the pathss length and the paths reactivity of the networks. PThe pThe path length is the number of edges in the path. The path length corresponds to the number of secondary sources between the primary and the final aggregators (PADI-web or HealthMap); for example, e.g.e.g., a path composed ofby three edges containscontain two secondary sources. We hypothesised that the fewer the number of sources in a path, the faster is the transmission of information was.

PThe path reactivity iwas the sum of the time lags between all the nodes composing the path. PThe path reactivity measuresd the number of days between the primary source's communication and the detection by the final aggregator. Path reactivity is highlytremendously relevant for EI, becauseas it reflects the ability of the system to quickly disseminate events to the aggregator.

Node analysis

We assessed the importance of the nodes, i.e., the sources, in the PADI-web and HealthMap networks using qualitative and quantitative attributes.

We first evaluated the global ability of the sources to receive and transmit event information by merging PADI-web and HealthMap networks. We calculated the in-degree, out-degree, and all-degree centrality measures of the nodes (18) and analysed their distribution according toby the types of sourcess. In-degree is the number of incoming edges to a node;, thus, sources with a high in-degree collect information from a large range of other sources. Out-degree is the number of outcoming edges from a node. Sources with a high out-degree are sources that are often cited;, thus, they arewhich able tocan communicate outbreak-related information with high visibility. The aAll-degree is the sum of the in-degree and out-degree. Sources with a high all-degree, also referred to as "hubs", combine bothe capacityies to receiveof receiving and shareing outbreak-related information (19).

We further analysed the role of the sources in the different subgraphs (early, late, and non-official), separating the PADI ~~-~~web and HealthMap networks. We classified the sources ~~them~~ according to their location~~place~~ in the network (primary versus secondary) and calculated the frequency of each type of source~~s~~ (e.g.~~,~~ online news~~, etc.~~). We further calculated the proportion of primary and secondary sources according to their geographical focus and ~~their~~ specialisation.

Software

The database was constructed using MS Office Access (version 2019). The a~~A~~nalysis was performed~~done~~ using the *igraph* package available in R version 3.6 (20).

Results

Event detection

Between 1~~st~~ July 2018 and 30~~st~~ June 2019~~,~~ national animal health authorities reported 351 AI outbreaks int~~o~~ the WOAH. Among these, 81% (284/351) were from~~outbreaks were in~~ domestic birds, 10% (34/351) were from~~in~~ wild birds, 6% (24/351) were from environmental samples, and 3% (12/351) were unspecified~~not specified~~.

The PADI-web detected 408 unique AI outbreak-related news reports,~~;~~ 337 (83%) of which~~them~~ were considered ~~as~~ relevant after manual curation (see details in S2 Table). HealthMap detected 163 unique AI outbreak-related news reports,~~;~~ 115 (71%) of which~~them~~ were~~being~~ relevant after manual curation. Among the relevant reports, 37 were detected using~~by~~ both the EBS systems.

Both the PADI-web and HealthMap had a median of one event per news report (min=1, max=14). In the PADI-web relevant news reports, ~~a total of~~ 230 events were described, including 193 events that were not detected by HealthMap (Table 1). Among the detected events, 87% (199/230) were official events; that is, they ~~, i.e.,~~ matched a notified AI outbreak to the WOAH. The remaining 31 events (13%) were unofficial, that is~~i.e.~~, they could not be verified. The majority~~, i.e.~~ (82%) of PADI-web events~~)~~~~, 82% of PADI-web events,~~ described AI outbreaks in domestic birds (185/226), while AI outbreaks in wild birds represented 13% (29/226) of the events.

HealthMap relevant reports described 68 events, among which 31 did not overlap with PADI-web detected events (Table 1). Among these events, 88% (60/68) were official ~~events~~ and 12% (8/68) were non-official~~ events~~. Similar to the PADI-web, 78% (53/68) of the HealthMap events were in domestic birds, whereas~~while~~ 16% (11/68) were in wild birds.

The non-overlapping events represented 45% (222/489) of all ~~the~~ events detected ~~events~~ by PADI-web and HealthMap.

Table 1. Number of official and non-official events of AI detected by PADI-web and HealthMap between July 2018 and June 2019. The number of non-overlapping events is shown between parentheses.

	PADI-web		HealthMap	
Type of host	Official	Non-official	Official	Non-official
Domestic birds	174 (147)	15 (13)	48 (23)	5 (3)
Wild birds	16 (10)	13 (12)	9 (3)	2 (1)
Mammals	-	2 (1)	-	1 (0)
Environmental	8 (8)	-	2 (0)	-
Unspecified	1 (1)	1 (1)	1 (1)	-
Total	199 (166)	31 (27)	60 (27)	8 (4)

The ~~sensitivitiesy~~Se of HealthMap and PADI-web were~~was~~ 17% (60/351) and 57% (199/351), respectively. The number~~s~~ of events reported to the WOAH and the events detected by the two EBS tools per week and ~~per~~ region ~~are~~are provided in the S3 Table.

The timeliness of PADI-web varied from 112 days before~~, up~~ to 39 days after ~~a~~ notification of an outbreak to the WOAH; 24% (47/199) of the events detected by PADI-web were detected before their official notification, representing 13% of the official events (Fig 1). The PADI-web was timelier in detecting AI events in wild birds than in~~in comparison to~~ domestic birds. More precisely, 21% (36/174) of the AI outbreaks in domestic birds in the PADI-web were detected before their official notification, while 56% of the events (9/16) were detected early in wild birds, with a maximum of 112 days before official notification in wild birds.

The timeliness of HealthMap varied from 46 days before~~, up~~ to 66 days after an official reporting of an event to the WOAH; 43% (26/60) of the events detected by the tool were reported before the official notification, representing 7% of the official events (Fig 1). In the HealthMap network, 42% (20/48) and 56% (5/9) of AI outbreaks in domestic ~~birds~~ and ~~in~~ wild birds, respectively, were detected before their official notification, with a maximum of 43 days before official notification in wild birds.

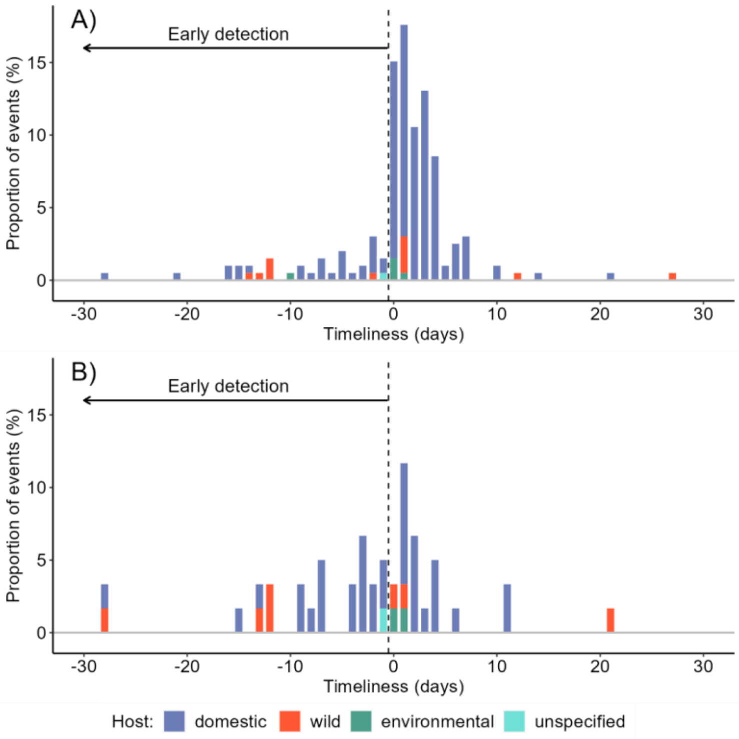

Fig 1. Timeliness in the detection of AI outbreaks according to the type of host for A) PADI-wWeb and B) HealthMap. ~~Y axis represents the proportion of events compared to the total number of detected events by each EBS tool.~~ For visibility, extreme values i.e., less than 30 days and higher than 30 days are not shown.

Network analysis

Network description

*1*During the study period, the PADI-web network disseminated AI outbreak-related information from 250 different nodes (~~i.e.,~~ sources), 446 unique edges (~~i.e.~~ links), and 455 paths. The 2HealthMap network comprised~~was made up of~~ 108 nodes, 150 unique edges, and 107 paths. A graphical representation of both networks, as well as detailsed of the edges and nodes, are provided in S4-7 Tables and S1 Fig.

Table 2. Types of sources (i.e., nodes) in PADI-web and HealthMap networks disseminating outbreak-related news on Avian influenza between 1st July 2018 and 30th June 2019

Type of source	PADI-web	HealthMap
online news source	47.6% (n=119)	36.1% (n=39)
national vet authority	14% (n=35)	20.4 % (n=22)
local veterinary authority	13.2% (n=33)	8.3 % (n=9)

Formatted: No underline, Font color: Auto

Formatted: Font: Bold

local official authority	6% (n=15)	3.7% (n=4)
press agency	4.8% (n=12)	10.2% (n=11)
radio, TV	4.4% (n=11)	3.7% (n=4)
laboratory	2.4% (n=6)	2.8% (n=3)
national official authority	2% (n=5)	5.6% (n=6)
research organisation	1.6% (n=4)	1.9% (n=2)
local person	1.2% (n=3)	0
social platform	1.2% (n=3)	4.6% (n=5)
private company	0.8% (n=2)	0
EBS tool	0.4% (n=1)	1.9% (n=2)
international veterinary authority	0.4% (n=1)	0.9% (n=1)
Total	250	108

Online news was~~were~~ the most represented ~~type of~~ sources~~,~~ (47.6% of the sources in the PADI-web network and~~,~~ 36% in the HealthMap network (Table 2). ~~-~~Local veterinary authorities were more frequent in the PADI ~~-~~web network than in the HealthMap network. Conversely, press agencies represented 10.2% of the HealthMap network sources, compared to~~against~~ 4.8% in the PADI-web network.

Path analysis

Most of the PADI-web paths are composed of two (232/455; 51%) and three (182/455; 40%) edges, 4% (18/455) of the paths are composed of a~~one~~ single edge~~s~~ (they do not cite any source), and 5% (21/455) of the paths are made up of four edges and more. Similarly, most ~~of the~~ HealthMap paths are composed of two (53/107; 50%) and three (32/107; 30%) edges, 14% (15/107) of the paths are composed of one edge~~link,~~ and 5% (7/107) are~~is~~ composed of five~~5~~ edges.

In the PADI-web,~~ the reactivity of~~ 83% (376/455) of the paths propagated events in ~~werewas~~ one day (n=41) or less than one~~a~~ day (n=335). Similar results were observed in HealthMap, with 94% (87/107) of the paths~~s~~ propagating events ~~s~~ in one day (n=3) or less than one~~a~~ day (n=84).

Quantitative node analysis

Only 24% (69/287) of the sources in the global network of the PADI-web and HealthMap were characteris~~z~~ed by an in-degree greater than 1, indicating that most of the sources received information from a single source. The EBS tools, PADI-web and HealthMap, international veterinary authority, social platforms, press agencies, and research organisations had the highest median in-degrees (Fig 2).

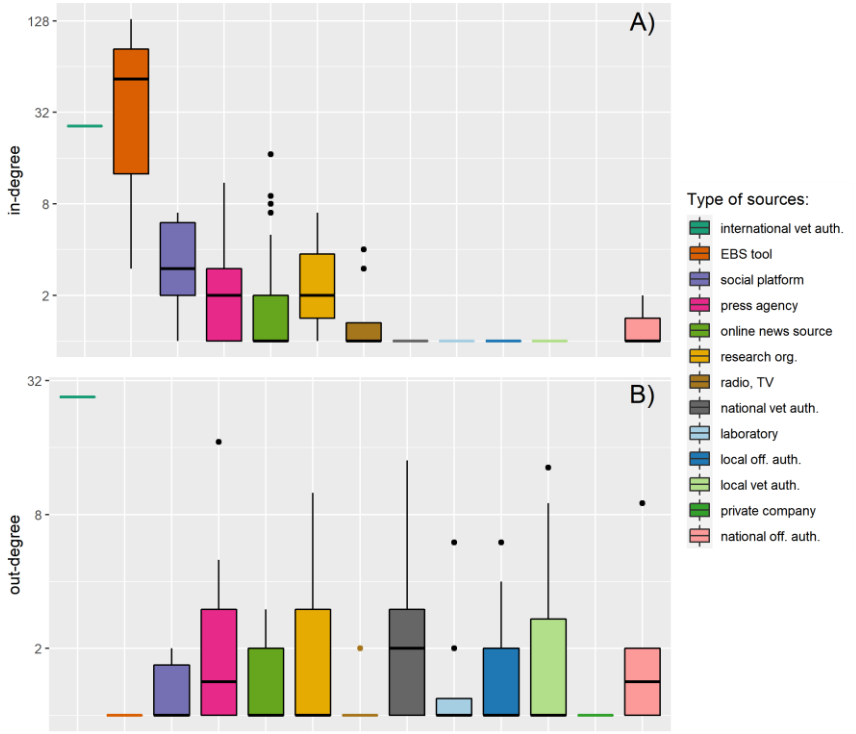

Fig 2. Performance of sources in terms of A) in-degree and B) out-degree, aggregated by type. The y-axis has been log-scaled. Distributions of in-degree and out-degree are represented with box plots based on a 95% confidence interval (outliers are represented with dots).

Formatted: Left

Formatted: Font: Bold, Font color: Text 2

These groups contain sources which have access to a large amount of information, that is~~i.e.~~, different sources.~~-~~ The EBS tools had the highest median in-degree because they included PADI-web and HealthMap, the two aggregators in our study. Except for these two EBS tools, the WOAH stood out with a~~the~~ maximal in-degree~~,~~ equal to 26.~~-~~ Online news sources were characteris~~z~~ed by a median in-degree of one, but twelve outliers had an in-degree higher than 5, among which "Times of India", and two sources specialis~~z~~ed in poultry production, "PoultrySite" and "WATTAgNet" (Table 2). Similarly, the social platforms, press agencies, and research organisations were characteris~~z~~ed by a high ~~variance~~ intra-group variance, containing highly connected sources (e.g.~~,~~ Reuters, Xinhua).

The median out-degree of nine~~9~~ out of the 13 types of sources was one, explained by the fact that 64% (183/297) of the sources in the networks were cited only once. Local and national

veterinary authorities had highe~~st~~rst out-degree values than in-degree values, highlighting their role as ~~emitter~~ sources of information.~~-~~ Individually, the WOAH stands out with the maximal out-degree (27), followed by Reuters, one national authority, and one local veterinary authority (Table 2). As for in-degree, the out-degree variance was high in most groups, owing~~due~~ to the presence of outliers being significantly better transmitters than the other sources of their group.

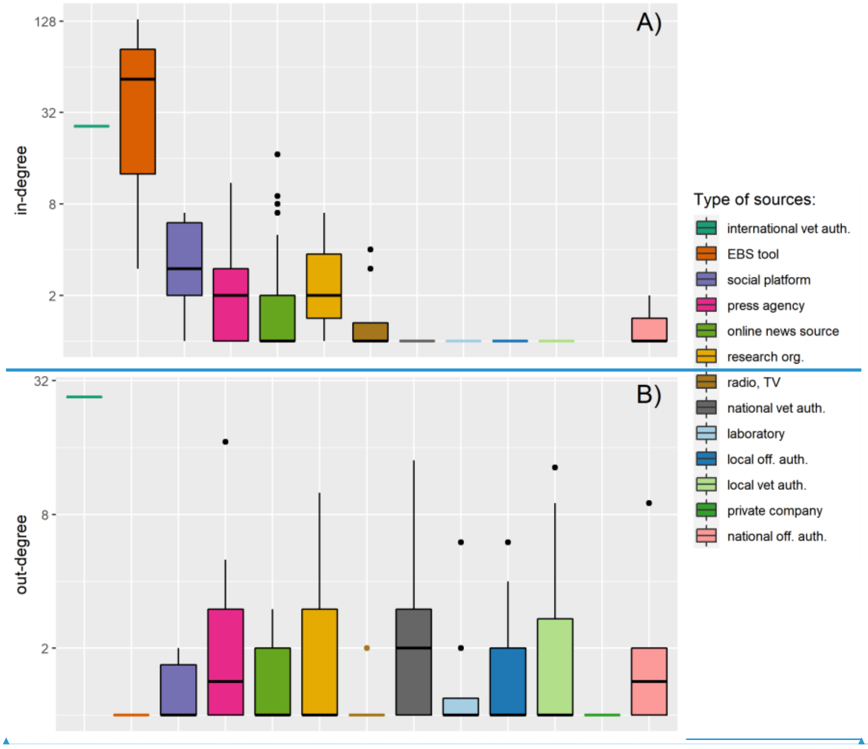

~~**Fig 2. Performance of sources in terms of A) in-degree and B) out-degree, aggregated by type.** The y-axis has been log-scaled. Distributions of in-degree and out-degree are represented with box_plots based on a 95% confidence interval (outliers are represented with dots).~~

~~The~~ WOAH was the ~~best~~ best-performing source in terms of all ~~-~~degrees, confirming its central position. It was followed by two press agencies, Reuters and Xinhua, the veterinary authority of Bulgaria, and ~~an~~ Indian online news, Time of India (Table 2).

Table 2. Top-5 sources in terms of in-degree, out-degree and all-degree. The EBS tools PADI-web and HealthMap were excluded as they were chosen as the aggregators in our study.

Formatted: Font: 11 pt

Formatted: Font: 11 pt

Formatted: Font: 11 pt

	Source	Value	Type
**In-degree**	WOAH	25	International vet auth.
	Times of India	17	Online news
	Xinhua	11	Press agency
	The Poultry Site	9	Online news
	WATTAgNet	8	Online news
**Out-degree**	WOAH	26	International vet auth.
	Reuters	17	Press agency
	Bulgaria Vet Auth	14	National vet auth.
	Minnesota Vet Authorities	13	Local vet auth.
	USA National Oceanic and Atmospheric Administration	10	Research org.
**All-degree**	WOAH	51	International vet auth.
	Reuters	24	Press agency
	Times of India	20	Online news
	Bulgaria Vet Auth	15	National vet auth.
	Xinhua	14	Press agency

Qualitative nodes analysis

National veterinary authorities were the most frequent primary source of events in the late detection of events in both HealthMap and PADI-web (69% and 63% of the primary sources, respectively)~~,~~ and ~~in~~ the early detection of HealthMap events (42% of the secondary sources) (Figs 3 and 4; detailed numbers in S8-9 Tables). Local veterinary authorities were the most frequent primary source involved in the early detection of events by the PADI-web (44% of the primary sources)~~,~~ and the second most frequent in HealthMap. The transmission of events in the late detection context was mainly driven by WOAH, press agencies, and online news for both the EBS tools. The transmission of events in the early detection context was mainly driven by online news sources (69% and 58% of the secondary sources in PADI-web and HealthMap, respectively), and press agencies were~~being~~ less frequent than in the early detection networks.

Social platforms represented 13% of the secondary sources involved in the early detection by HealthMap, whereas~~while~~ this type of source was barely used by the PADI-web.

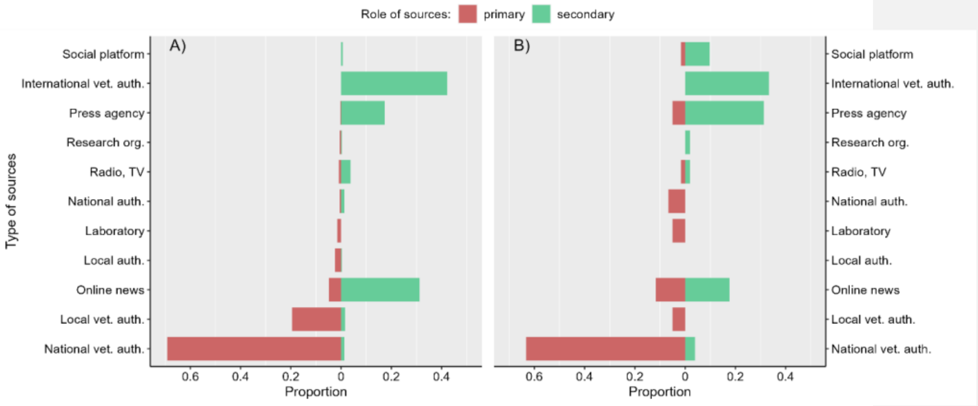

Fig 3. Proportion of the types of primary and secondary sources according to their role in the (a) PADI-web and (b) HealthMap late detection networks. Primary sources are sources that are the first to emit an event, secondary sources are sources which receive and emit an event to another source.

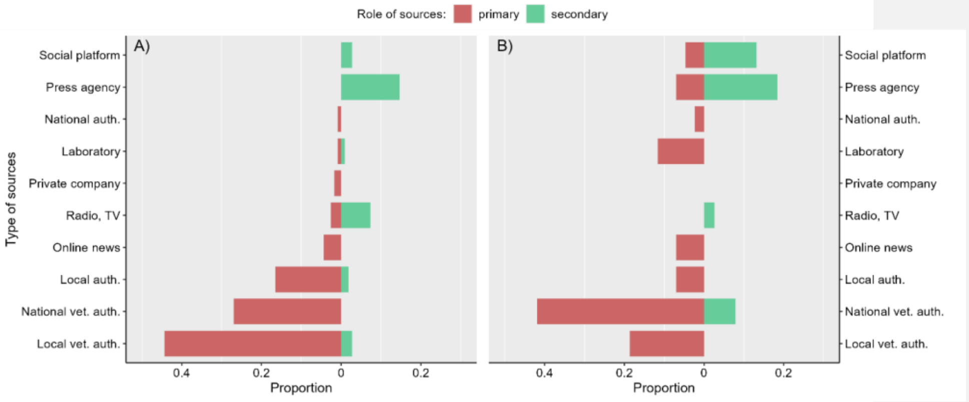

Fig 4. Proportion of the types of primary and secondary sources according to their role in the (a) PADI-web and (b) HealthMap early detection network. Primary sources are sources that are the first to emit an event, secondary sources are sources which receive and emit an event to another source

Nearly 75% of the primary sources in the early detection network of the PADI-web had a local geographical scope, in contrast~~opposite~~ to 26% in HealthMap (Fig 5). This result was consistent with our previous results, highlighting the role of local sources in the early warning of disease outbreaks. The late detection networks mainly relied on sources with a national scope for both EBS tools, corresponding to the role of the national veterinary authorities.

Early detection networks relied on both national and international sources as intermediates, while late detection was mostly driven by international sources, as explained by the role of the WOAH in the official communication of events in the news.

~~The~~ S~~s~~pecialis~~z~~ation showed the same pattern between late and early detection and between the EBS tools, with at least 75% of the primary sources being specialis~~z~~ed (S1 Fig).

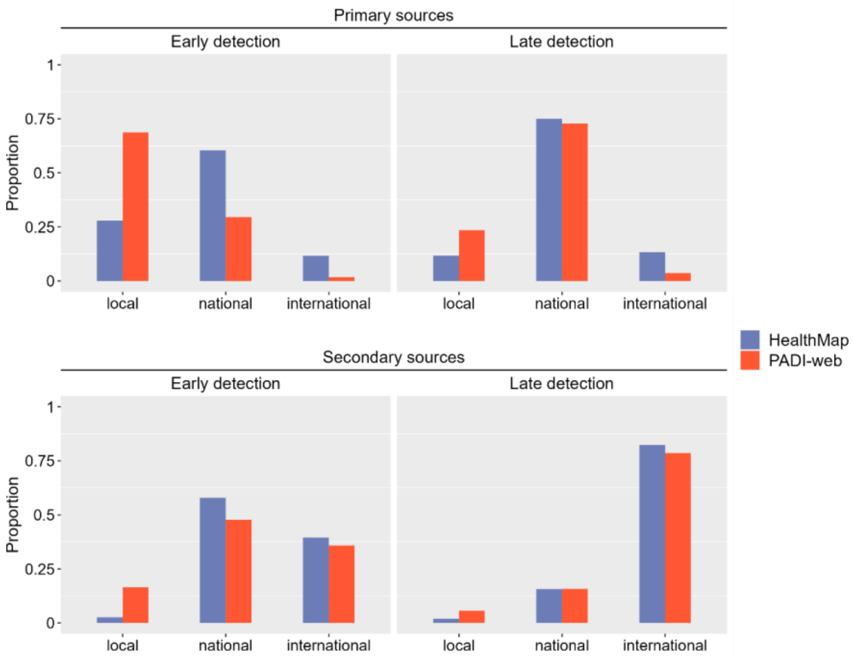

Fig 5. Proportion of the geographic scope of primary and secondary sources in the PADI-web and HealthMap early and late detection networks.

Discussion

In this work, we ~~have~~ described ~~for the first time~~ how outbreak-related information circulates in ~~the~~ news sources captured by two EBS tools, PADI-web and HealthMap. We assessed the EBS tools network, including primary and secondary sources, and their characteristics in terms of type, geographical scope, specialisation, and importance in the dissemination of information using network centrality metrics. In addition, we ~~have~~ assessed ~~these~~ the timeliness of ~~officially~~ sharing officialy ~~to share officially~~ notified AI outbreak information.

Global performances of PADI-web and HealthMap networks

PADI-web and HealthMap, to a varying extents, capture false positive news reports (with respective report precisions of 83% and 71%, respectively). Even if considered as irrelevant for the purpose of this study, most of the discarded news reports were related to AI events and contained contextual epidemiological information useful for risk assessment purposes, such as protective and control measures or global overviews of AI in a specific region. Both tools awere prone to classifying human-related reports as animal-related events. When correctly identified, the the detection of zoonotic events in humans is highly relevant from a One hHealth perspective. The automatic fine-grained topic classification of news reports still needs improvements to enable discriminationdiscriminating of outbreak declarations from other topics, thus avoiding false alerts and facilitatinge the triage of sanitary information (21).

The PADI-web was more sensitive thancompared to HealthMap. However, the proportion of early detected events compared to the total number of detected events was higher for HealthMap (43% vs.versus 23%). These differences in captured events may reflect the different web scraping and filtering methods for online news monitoring of the PADI-web and HealthMap. PADI-web is an entirely automatiszed tool;, thus, it captures and filters outbreak-related information without any human intervention. HealthMap is a semi-automatised tool withsemi-automatized tool, it has human moderators that filter which news reports that will be shared with users. ThisIt may suggest that HealthMap moderators filter and keep only emerging, exceptional AI events (such as primary cases), rather than all possible AI events (primary and secondary cases).

Our study highlightsed the complementarity of these two EBS tools. This complementarity reflects the different sources accessed through the EBS pipelines. Our results showed, for instance, that PADI-web captured more local sources than HealthMap, while the latter relied more heavily relied on social platforms such as Twitter. Barboza et al. (10) showed that the EBS tools characteristics such as the type of moderation, the sources accessed, diseases, languages, and regions covered significantly influence disease detection performance, and that the system's outbreak detection isare synergic (complementary). While the proportion of early detected events in our study may seem modest, it is yet a -significant added -value of to the EBS regarding the reporting ofn outbreaks of pathogens with a zoonotic and pandemic potential. In addition, both networks were highly reactive, mostly propagating the information from primary sources to the aggregator in less than onea day. Early detection of public health hazards constitutes a first and fundamental component of efficient outbreak

management (22). It may be the main determinant in selecting the appropriate response,~~, and~~ thus minimis~~z~~ing ~~the~~ morbidity and mortality caused by an infectious disease (23). Event-based surveillance should not be considered a replacement for traditional indicator-based surveillance, but rather, complementary to routinely collected public health surveillance data.

While the reporting of AI events by the EBS tools was highly effective, timely, and reactive, a bottleneck may arise at the step of manual analysis of the detected events. The strength of EBS relies heavily ~~relies~~ on adequate human resources to feed decision-~~-~~making chains based on detected events. Therefore, in our future work, we will~~aim to~~ explore how the detected events can be useful for risk assessment and risk mapping.

Role of the sources

Our results highlight~~ed~~ three groups of sources regarding their role in the dissemination of outbreak-related information. ~~The~~ EBS tools a~~we~~re ~~the~~ aggregators. It is important to note that our results did not reflect ProMED-mail ~~Pro-MED~~ intrinsic performance~~s~~ as an EBS tool, that is~~i.e.~~, expert network sharing ~~outbreak~~ outbreak-related information, but as an intermediate source of HealthMap. Local and national authorities and~~,~~ veterinarians~~veterinary or not,~~ were emitters and~~,~~ were~~being~~ the most important primary sources of events. They produce ~~an~~ information that is acknowledged at the local/national level, mostly verified by laboratory tests, and is susceptible to being reported in the media. ~~The~~ WOAH, online news, press agencies, social media, and several research organisations combined both abilities by collecting information from a wide range of sources and being highly visible by collector sources in the network (online news, EBS tools).~~–~~ N~~The n~~etwork performance~~s~~ was~~were~~ driven by the presence of a small number of sources with high individual all-degrees, such as WOAH, Reuters,~~, and~~ Xinhua, and several social network platforms. These sources played the role of hubs, not only filtering and disseminating information but also ensuring a connection between different groups in the network (19). The presence of hubs was not the only feature of~~r~~ ~~the~~ network performance, as ~~the~~ early detection mostly relied on online news sources with individual low all-degrees. Thus, the early components of ~~the~~ EBS networks also relied on their ability to monitor a large number of individually low-performant sources.

National online news plays~~ed~~ a major role in early detection by disseminating announcements from local and national veterinary authorities, thus making them ~~detectible~~ detectable by EBS tools. Zhang et al. found out that national newspapers (referred to as "local" newspapers in their methods) provided more specific information ~~of~~ about the local Zika virus emergence in

Brazil than did international newspapers~~.~~; similar findings were made for outbreak detection in Nepal (12). In a~~the~~ recent~~latest~~ study, local sources were more likely to identify a unique event than international sources, indicating that international sources were more likely to be redundant by publishing multiple reports about the same event (18). This emphasis~~z~~es the need to target~~of targeting~~ local and national sources available on the web, going beyond sources published in English. The monitoring of multi-lingual sources, integrated into the two EBS tools in~~of~~ our work, is a prerequisite for maximis~~z~~ing ~~the~~ access to national and local media. The retrieval and analysis of non-English texts have~~s~~ been enhanced and facilitated by the improvement of methods for ~~in~~ multi-lingual text~~s~~ processing, such as textual classification (25,26) and deep-learning-based translation (27). We believe that ~~pursuing~~ efforts to~~in~~ integrate~~ing~~ multi-lingual sources will benefit ~~to~~ both the Se~~sensitivity~~ and timeliness of EBS tools.

Social platforms, mostly used by HealthMap, include~~d~~ generic platforms~~,~~ such as Twitter, but also specialis~~z~~ed blogs such as FluTrackers and AvianFluDiary. Specialis~~z~~ed blogs are relevant sources for integration~~to integrate~~ into EBS, as they rely on the collection of information from numerous sources, as highlighted by their high median in-degree, previously filtered by domain-specialis~~z~~ed moderators. Health blogs were found to cite less sources than online news in a study evaluating H1N1/Swine Flu coverage in the media (28), which is not in line with the~~ir~~ highest in-degree found in our study. However, the difference in the number and nature of sources evaluated (eight~~8 in~~ online news~~ in~~ (28)) makes the study hardly comparable. They also translated news from national languages into English, facilitating ~~the~~ access to local field information. In addition~~Besides~~, owing~~thanks~~ to their non-official status, online blogs are more prone to communicate events before official notifications. While the classical method~~way~~ of web monitoring i~~wa~~s traditionally keyword-oriented (~~e.g.~~e.g., systematic monitoring of combinations of ~~keyworks~~keywords), ~~the~~ source-based monitoring (~~i.e.~~i.e., systematic monitoring of a specific source) is~~would be~~ a costless and easy way to improve existing EBS tools. For instance, retrieving news directly from official government health websites would enhance the geographic representativeness of news aggregators such as Google News (29,30).

Commented [A4]: Dear author, the sentence was unclear to m. Do you mean, “the news retrieved directly from official government health websites would be released in the absence of the geographic representativeness of news aggregators such as Google News”. Kindly clarify what you mean here so that I can revise.

It is important to note that our results were specific to the model disease and ~~the~~ study period. For example, the Bulgarian veterinary authority appeared to be~~as~~ an important source because 22 outbreaks were observed in Bulgaria during the study period, including a new incursion of the Highly Pathogenic Avian Influenza (HPAI) H5N8 subtype (31) widely reported by Bulgarian media~~s~~.

Re-thinking the role of event-based surveillance in epidemic intelligence

EBS is sometimes opposed to indicator-based surveillance, as it is based on the use of so-called non-official sources. In our study, official veterinary authorities (national or local) represented 80% of the primary sources, including thosethe ones involved in early detection. Thus, the monitoring of the PADI-web and HealthMap was mainly characteriszed by the detection of nationallly or locallly official events. This detection includes both the dissemination of WOAH-notified outbreaks (late detection) and the dissemination of official events that have not yet been notified (early detection). In the latter case, EBS tools by-pass the procedure of the international notification procedure and its inherent delays. These findings are consistent with the latest and broader definitions of the EBS, stating that media sources collected in the context of EBS can be either official (e.g. a MMinistry of HHealth website) or non-official (e.g. newspaper) (32).

AlthoughWhile the extraction of epidemiological information from collected reports has been widely studied, the automatic extraction of the cited sources of events from online sources has not yet received attention. However, based on the findings of our study, we believe that this feature would enhance informal surveillance by enabling theo characterisation ofze an event as official at the international, national, or local level, depending on whetherif the cited source is the WOAH, or a national/local veterinary authority, or non-official, if the type of source does not belong to any of the latest categories. Recent advances in named entityies extraction, involving deep learning, combined with a step of normalisation (dictionary or ontology-based), would enable to easily identification ofy the mentioned of the cited sources. Alerts could be triggered when WOAH is not mention to the WOAH is detected. By providing our corpus and databases withas openan open-access, we offer the possibility of evaluatingto evaluate and comparinge approaches with a high-quality validation dataset.

Both the EBS tools detected several events that could not be found in the EMPRES-i database (S10 Table). These events may have been local AI events that were not communicated at the international level;, thus, they dido not appear in the EMPRES-i database. They may also correspond to a suspected event that was negated after a negative laboratory test results for the AI virus, or to a false alert, as mentioned in a previous studywork (33). Thus, our study shows that EBS tools can be a source of relevant outbreak information, but should be consideredlooked as complementary to the official sources and interpreted with caution. The identification and characteriszation of the sources linked in an EBS is are important for

prioritising~~to prioritise~~ the ones regarding truthfulness and reliability. It may be a way~~manner~~ of dealing with fake news, for example, by targeting specialised sources~~, by targeting sources that are specialised, for example~~. Our study sets the~~a~~ first list of these sources. By extending our approach to emerging zoonotic infectious diseases, the ~~these~~ corpora of reliable news sources may be enriched.

Conclusion

Current EBS tools use a diverse, but not identical, network of sources;~~,~~ thus, they can~~to~~ be used in parallel by EI practitioners. In addition, both EBS tools should prioritise specialised media sources and access, when existing, to local and national veterinary authorities' webpages, as they released part of the official event before the international notification to the WOAH. Outbreak-related news travels from a primary source to a final aggregator ~~for~~ in one day or less, which is ~~of~~ importan~~tce~~ for~~to~~ early warnings and EI~~epidemic intelligence~~. Both~~,~~ PADI-web and HealthMap shared timely outbreak information on AI in domestic and wild birds, thus contributing to~~wards~~ the early detection ~~aspect~~ of EI~~epidemic intelligence~~ and as complementary sources to traditional surveillance.

A potential future work could be the integration of the results highlighted~~ing~~ in this study ~~in order~~ to improve EBS systems (for instance, by weighting type of sources in EBS platforms). As mentioned in this paper, we can cite multi-lingual aspects to consider for improving the proposed analysis as well as~~but also the~~ EBS systems. We could evoke the same type of analysis to conduct with other platforms as well, such as~~for instance~~ ProMED-mail.

Acknowledgements

We thank the HealthMap project (https://healthmap.org/), which~~o~~ kindly provided us with their data. We acknowledge the reviewer~~s~~ for their constructive comments.

Data reporting

The data used for this study is available at:
https://doi.org/10.5281/zenodo.7324144

Statistical reporting

The code used for the analysis and figures is available at:
https://github.com/SarahVal/EBS-network.

Author Contributions

Sarah Valentin: cCConceptualiszation, mMMethodology, dDData cCCuration, fFFormal aAAnalysis, vVValidation, wWWriting – Original Draft Preparation, Writing – Review & Editing

Bahdja Boudoua: Data Curation, Formal Analysis, Writing – Original Draft Preparation, Writing – Review & Editing

Kara Sewalk: Data Curation, Writing – Review & Editing

Nejat Arinik: Visualization, Writing – Review & Editing

Mathieu Roche: Conceptualization, Supervision, Resources, Writing – Review & Editing

Renaud Lancelot: Conceptualization, Supervision, Resources, Writing – Review & Editing

Elena Arsevska: Conceptualization, Methodology, Data Curation, Writing – Original Draft Preparation, Writing – Review & Editing

Supporting information

S1 Table. Definitions used to characterize the types of sources, specialization and geographical focus in PADI-web and HealthMap networks.

S2 Table. Summary of the manual curation of the relevance of PADI-web and HealthMap reports.

S3 Table. The number of events reported to the WOAH and detected by the two EBS tools per week (mean, min, and max) and per region.

S1 Fig. PADI-web (A) and Healthmap (B) networks. Sources were grouped by type. The edge colour corresponds to the colour of the incoming source type, thus enabling the visualisation of the direction of information dissemination, that is, orange edges represent incoming edges to an EBS tool.

S4 Table. Legend of the node's names in the PADI-web network.

S5 Table. Legend of the node's names in the HealthMap network.

S6 Table. PADI-web network composition.

S7 Table. HealthMap network composition.

S8 Table. Proportion of the types of sources according to their role in the (a) PADI-web and (b) HealthMap late detection networks.

S9 Table. Proportion of the types of sources according to their role in the (a) PADI-web and (b) HealthMap early detection networks.

S2 Fig. Type of specialization of primary and secondary sources for the detection of early and late events in PADI-web and HealthMap networks

S10 Table. Type of primary and secondary sources involved in the detection and transmission of non-official events in PADI-web and HealthMap networks.

References

1. Paquet C, Coulombier D, Kaiser R, Ciotti M. Epidemic intelligence: a new framework for strengthening disease surveillance in Europe. Eurosurveillance. 1 déc 2006;11(12):5‑6.

2. Wilburn J, O'Connor C, Walsh AL, Morgan D. Identifying potential emerging threats through epidemic intelligence activities—looking for the needle in the haystack? International Journal of Infectious Diseases. 1 déc 2019;89:146‑53.

3. Kaiser R, Coulombier D, Baldari M, Morgan D, Paquet C. What is epidemic intelligence, and how is it being improved in Europe? Weekly releases (1997–2007). 2 févr 2006;11(5):2892.

4. Freifeld CC, Mandl KD, Reis BY, Brownstein JS. HealthMap: Global Infectious Disease Monitoring through Automated Classification and Visualization of Internet Media Reports. Journal of the American Medical Informatics Association. 1 mars 2008;15(2):150‑7.

5. Valentin S, Arsevska E, Falala S, de Goër J, Lancelot R, Mercier A, et al. PADI-web: A multilingual event-based surveillance system for monitoring animal infectious diseases. Computers and Electronics in Agriculture. 1 févr 2020;169:105163.

6. Bhatia S, Lassmann B, Cohn E, Desai AN, Carrion M, Kraemer MUG, et al. Using digital surveillance tools for near real-time mapping of the risk of infectious disease spread. npj Digital Medicine. 16 avr 2021;4(1):1‑10.

7. Arsevska E, Valentin S, Rabatel J, Hervé J de G de, Falala S, Lancelot R, et al. Web monitoring of emerging animal infectious diseases integrated in the French Animal Health Epidemic Intelligence System. PLOS ONE. août 2018;13(8):e0199960.

8. Valentin S, Mercier A, Lancelot R, Roche M, Arsevska E. Monitoring online media reports for early detection of unknown diseases: insight from a retrospective study of COVID-19 emergence. Transboundary and Emerging Diseases [Internet]. 19 juill 2020 [cité 28 juill 2020]; Disponible sur: https://onlinelibrary.wiley.com/doi/abs/10.1111/tbed.13738

9. Plateforme ESA [Internet]. [cité 1 nov 2022]. Disponible sur: https://plateforme-esa.fr/fr

10. Barboza P, Vaillant L, Le Strat Y, Hartley DM, Nelson NP, Mawudeku A, et al. Factors influencing performance of internet-based biosurveillance systems used in epidemic intelligence for early detection of infectious diseases outbreaks. PLoS ONE. 5 mars 2014;9(3):e90536.

11. Barboza P, Vaillant L, Mawudeku A, Nelson NP, Hartley DM, Madoff LC, et al. Evaluation of epidemic intelligence systems integrated in the Early Alerting and Reporting project for the detection of A/H5N1 influenza events. Nishiura H, éditeur. PLoS ONE. 5 mars 2013;8(3):e57252.

12. Schwind JS, Norman SA, Karmacharya D, Wolking DJ, Dixit SM, Rajbhandari RM, et al. Online surveillance of media health event reporting in Nepal: digital disease detection from a One Health perspective. BMC International Health and Human Rights. 21 sept 2017;17(1):26.

13. Xu Y, Gong P, Wielstra B, Si Y. Southward autumn migration of waterfowl facilitates cross-continental transmission of the highly pathogenic avian influenza H5N1 virus. Sci Rep. 10 août 2016;6(1):30262.

14. Mostafa A, Abdelwhab EM, Mettenleiter TC, Pleschka S. Zoonotic Potential of Influenza A Viruses: A Comprehensive Overview. Viruses. 13 sept 2018;10(9):497.

15. Welte VR, Terán MV. Emergency Prevention System (EMPRES) for Transboundary Animal and Plant Pests and Diseases. The EMPRES-Livestock: An FAO Initiative. Annals of the New York Academy of Sciences. 2004;1026(1):19‑31.

16. Farnsworth ML, Hamilton-West C, Fitchett S, Newman SH, de La Rocque S, De Simone L, et al. Comparing national and global data collection systems for reporting, outbreaks of H5N1 HPAI. Preventive Veterinary Medicine. 1 juill 2010;95(3):175‑85.

17. Kolaczyk ED. Statistical Analysis of Network Data: Methods and Models. 1st éd. Springer Publishing Company, Incorporated; 2009.

18. Kolaczyk ED. Descriptive Analysis of Network Graph Characteristics. In: Kolaczyk ED, éditeur. Statistical Analysis of Network Data: Methods and Models. New York, NY: Springer; 2009. p. 1‑44. (Springer Series in Statistics).

19. Weber MS, Monge P. The Flow of Digital News in a Network of Sources, Authorities, and Hubs. Journal of Communication. déc 2011;61(6):1062‑81.

20. Csardi G, Nepusz T. The igraph software package for complex network research. InterJournal. 2006;Complex Systems:1695.

21. Valentin S, Arsevska E, Vilain A, De Waele V, Lancelot R, Roche M. Elaboration of a new framework for fine-grained epidemiological annotation. Scientific Data. 26 oct 2022;9(1):655.

22. Adini B, Singer SR, Ringel R, Dickmann P. Earlier detection of public health risks – Health policy lessons for better compliance with the International Health Regulations (IHR 2005): Insights from low-, mid- and high-income countries. Health Policy. oct 2019;123(10):941‑6.

23. Yan SJ, Chughtai AA, Macintyre CR. Utility and potential of rapid epidemic intelligence from internet-based sources. International Journal of Infectious Diseases. 1 oct 2017;63:77‑87.

24. Yoon S, Odlum M, Broadwell P, Davis N, Cho H, Deng N, et al. Application of Social Network Analysis of COVID-19 Related Tweets Mentioning Cannabis and Opioids to Gain Insights for Drug Abuse Research. Stud Health Technol Inform. 26 juin 2020;272:5‑8.

25. Mutuvi S, Boros E, Doucet A, Jatowt A, Lejeune G, Odeo M. Multilingual Epidemiological Text Classification: A Comparative Study. In: Proceedings of the 28th International Conference on Computational Linguistics [Internet]. Barcelona, Spain (Online):

International Committee on Computational Linguistics; 2020. p. 6172-83. Disponible sur: https://aclanthology.org/2020.coling-main.543

26. Névéol A, Dalianis H, Velupillai S, Savova G, Zweigenbaum P. Clinical Natural Language Processing in languages other than English: opportunities and challenges. Journal of Biomedical Semantics. 30 mars 2018;9(1):12.

27. Singh SP, Kumar A, Darbari H, Singh L, Rastogi A, Jain S. Machine translation using deep learning: An overview. In: 2017 International Conference on Computer, Communications and Electronics (Comptelix). 2017. p. 162-7.

28. Gao F, Zhang M, Sadri S. Newspapers Use More Sources Compared to Health Blogs in H1N1/Swine Flu Coverage. Newspaper Research Journal. 1 mars 2011;32(2):89-96.

29. Feldman J, Thomas-Bachli A, Forsyth J, Patel ZH, Khan K. Development of a global infectious disease activity database using natural language processing, machine learning, and human expertise. J Am Med Inform Assoc. 30 juill 2019;26(11):1355-9.

30. Zhang Y, Ibaraki M, Schwartz FW. Disease surveillance using online news: Dengue and zika in tropical countries. J Biomed Inform. févr 2020;102:103374.

31. Zecchin B, Goujgoulova G, Monne I, Salviato A, Schivo A, Slavcheva I, et al. Evolutionary Dynamics of H5 Highly Pathogenic Avian Influenza Viruses (Clade 2.3.4.4B) Circulating in Bulgaria in 2019–2021. Viruses. oct 2021;13(10):2086.

32. Tu W, Jin L, Ni D. Early Warning Practice Using Internet-Based Data. Early Warning for Infectious Disease Outbreak. 2017;231-41.

33. Barboza P. Evaluation of epidemiological intelligence systems applied to the early detection of infectious diseases worldwide. 2015;122.

November 16, 2022

Subject: Response to the review of manuscript number PONE-D-22-24102

Dear PlosOne Chief Editor and Reviewers,

We acknowledge your comments on our manuscript "Dissemination of information in event-based surveillance, a case study of Avian Influenza". We addressed your constructive reviews by modifying our manuscript (using track changes) and answering the reviewers' questions here-below.

Best regards,
The authors

General comments from the editor

If applicable, we recommend that you deposit your laboratory protocols in protocols.io to enhance the reproducibility of your results. Protocols.io assigns your protocol its own identifier (DOI) so that it can be cited independently in the future. For instructions see: `https://journals.plos.org/plosone/s/submission-guidelines#loc-laboratory-protocols`.

1. Please ensure that your manuscript meets PLOS ONE's style requirements, including those for file naming. The PLOS ONE style templates can be found at :

- `https://journals.plos.org/plosone/s/file?id=wjVg/PLOSOne_formatting_sample_main_body.pdf`, and
- `https://journals.plos.org/plosone/s/file?id=ba62/PLOSOne_formatting_sample_title_authors_affiliations.pdf`

- Author affiliations formatting. We have added the appropriate pilcrow symbol for the equal contributors of the work. We have set the appropriate format for the corresponding author. We have fixed the affiliations, by removing postcodes and removing abbreviations of Departments and listing all institutions in full. Please check page 1 of the manuscript.

- Manuscript body formatting. We have adjusted level 1 heading for all major sections. File formats for figures were corrected, now they are in .tiff format and passed via the PACE tool suggested by PlosOne.

2. We note that the grant information you provided in the 'Funding Information' and 'Financial Disclosure' sections do not match. When you resubmit, please ensure that you provide the correct grant numbers for the awards you received for your study in the 'Funding Information' section.

- Done. Funding from Acknowledgments section has been removed and moved into the 'Funding Infor-

mation' and 'Financial Disclosure' sections. Please see the new Acknowledgments section in line 546.

3. Thank you for stating the following in the Acknowledgments Section of your manuscript: ”This work has been funded by the “Monitoring outbreak events for disease surveillance in a data science context” (MOOD) project from the European Union's Horizon 2020 research and innovation program under grant agreement No. 874850 (`https://mood-h2020.eu/`) and is catalogued as MOOD 049.”

We note that you have provided funding information that is not currently declared in your Funding Statement. However, funding information should not appear in the Acknowledgments section or other areas of your manuscript. We will only publish funding information present in the Funding Statement section of the online submission form.

Please remove any funding-related text from the manuscript and let us know how you would like to update your Funding Statement. Currently, your Funding Statement reads as follows: ”The funders had no role in study design, data collection and analysis, decision to publish, or preparation of the manuscript.” Please include your amended statements within your cover letter; we will change the online submission form on your behalf.

- Done. Funding from Acknowledgments section has been removed and moved into the 'Funding Information' and 'Financial Disclosure' sections.

- Please continue to use the current Funding Statement: ”The funders had no role in study design, data collection and analysis, decision to publish, or preparation of the manuscript.”

4. In your Data Availability statement, you have not specified where the minimal data set underlying the results described in your manuscript can be found. PLOS defines a study's minimal data set as the underlying data used to reach the conclusions drawn in the manuscript and any additional data required to replicate the reported study findings in their entirety. All PLOS journals require that the minimal data set be made fully available. For more information about our data policy, please see `http://journals.plos.org/plosone/s/data-availability`. Upon re-submitting your revised manuscript, please upload your study's minimal underlying data set as either Supporting Information files or to a stable, public repository and include the relevant URLs, DOIs, or accession numbers within your revised cover letter. For a list of acceptable repositories, please see `http://journals.plos.org/plosone/s/data-availability#loc-recommended-repositories`. Any potentially identifying patient information must be fully anonymized.

- We created a Zenodo repository (`https://doi.org/10.5281/zenodo.7324144`) containing the entire dataset to reproduce the results. We provided the link in the manuscript, section Data reporting, line 549.

- We also shared the script for our results presented in the manuscript in a public GitHub repository (`https://github.com/SarahVal/EBS-network`). We provided the link in the manuscript, section Statistical reporting, line 552.

- Our dataset does not contain patient information.

Important: If there are ethical or legal restrictions to sharing your data publicly, please explain these restrictions in detail. Please see our guidelines for more information on what we consider unacceptable restrictions to publicly sharing data: `http://journals.plos.org/plosone/s/data-availability#loc-unacceptable-data-access-restrictions`. Note that it is not acceptable for the authors to be the sole named individuals responsible for ensuring data access. We will update your Data Availability statement to reflect the information you provide in your cover letter.

- There are no legal and ethical restrictions for sharing our dataset publicly. Please check the description of our dataset at: `https://doi.org/10.5281/zenodo.6908000`

5. Please upload a new copy of Figure 3 as the detail is not clear. Please follow the link for more information: `https://blogs.plos.org/plos/2019/06/looking-good-tips-for-creating-your-plos-figures-graphics/`

- All figures have passed though the PACE web-based imaging review tool. We provide you with new figure publication graphics in a .tiff format, uploaded separately. For clarity, we have moved Figure 3 into Supp material.

Comments from reviewer 1

Line 35: Please write what WOAH means.

- Done, we defined World Organisation for Animal Health (WOAH, founded as OIE), line 159. We further checked for all other acronyms and their first mention full description.

Line 165: there's a N staring the sentence (also in lines 276 and 278 that are starting with numbers). Please check

- Removed in line 165, it was a typing error. However, we did not find typos for numbers for lines 276 & 278.

Within the results section, what do authors mean by unique events in Table 1?

- A unique event, non-overlapping event, as initially defined in our manuscript, was an event detected by either of the event-based surveillance (EBS) tools, PADI-web or HealthMap. More precisely, a unique event was an event event detected by PADI-web (or by HealthMap, respectively) and not detected by HealthMap (or by PADI-web, respectively). To avoid confusion, we replace the term "unique" by "non-overlapping". Non-overlapping events enable us to analyse the overlap (and, thus, the complementary) between HealthMap and PADI-web. We provide an improved description of the term "unique event" in the manuscript in the section Material and methods, section Event detection line 166 and in the Results, section Event detection lines 266-271.

Figure 3 is impossible to read. Could the authors improve the image quality?

- All figures have passed though the PACE web-based imaging review tool. We provide you with new figure publication graphics in a .tiff format, uploaded separately. For clarity, we have moved Figure 3 into Supp material.

Comments from reviewer 2

Introduction

First paragraph: The manuscript refers to communication in health surveillance and how it can be expanded in the case of avian influenza. Which bibliographic reference of the world health organization that guides or suggests the use of the dissemination of information on health-related events?

- We added references to the Epidemic Intelligence paradigm, which promotes the use of non-official sources to follow the dissemination of information on health-related events and complement indicator-based surveillance. We have in detail reworked the introduction, please check pages 3 and 4.

What context do these Padi-web and HealthMap applications work in? The first paragraphs do not mention health surveillance and its emergencies where these programs/applications can be useful.

- PADI-web and HealthMap facilitate the collection, analysis and dissemination of event-based surveillance data on infectious diseases and associated health issues, in the context of epidemic intelligence. Several studies have assessed their use and performances in different epidemiological contexts including new and enzootic, epizootic and zoonotic infectious diseases. We provide example and new references in the manuscript. We have in detail reworked the introduction, please check pages 3 and 4.

Second paragraph: it is not clear and explanatory all the advantages of using healthy maps descriptors. It must be in simple and clear computational language, after all, the target audience is not only the scientific community, but health workers.

We specified the audience and simplified the description of both tools in the manuscript. We have in detail reworked the introduction, please check pages 3 and 4.

-Seventh paragraph, last line: What is your source of comparison in relation to the healthy map data? what is the assumption or hypothesis that it can be more useful ?

- In the seventh paragraph, we refer to a former study that evaluated the role of the sources detected by HealthMap regarding the detection of outbreaks, at a national scale (Nepal). The gold standard database with which the authors compared HealthMap was the official country outbreak notifications. We motivate our study as an extension of this work, by providing two significant enhancements: (1) we enlarge this work on a global scale and (2) we do not solely rely on the sources directly detected by the EBS tools, but we trace back the origin of the outbreak information. We have in detail reworked the introduction, please check pages 3 and 4.

Regarding the questions of this work

1. What are the sources involved in the reporting of outbreak-related information on the web?- This would not be a question but a methodology to evaluate.

- Every EBS media monitoring tool in use today has its own methodology for detection of sources on the web, collection, filtering of news and extraction of relevant information from the unstructured text from the news. The sources detected by an EBS tool result from (1) the choice of targeting a specific source (e.g. HealthMap collect Pro-MED alerts) and (2) its methodological choices (e.g. keywords to capture the news, languages for the keywords, Google news regions to monitor, etc.). In the last case, the specific online news that will be captured cannot be know *a priori*. In our work, we do not solely evaluate the sources directly detected by the EBS tools, but, we also trace back and characterise the initial sources first emitting the disease outbreak information (referred to as primary sources in our manuscript) and the intermediate ones, based on the manual evaluation of all sources cited in each news, which was a fastidious work of data collection and curation for the co-authors. We provide a clarification on this objective in the introduction.

3. How complementary are the different EBS tools in terms of monitored sources and reported outbreak-related information?—Is it compared to which data?

We address this question in two steps. First, we calculate the proportion of overlapping events (events that were detected by both PADI-web and HealthMap), We show that almost half of the detected events were non-overlapping events. Second, we show that the two tools do not monitor the same sources (i.e. PADI-web retrieved a largest number of online news sources, while HealthMap retrieved content from more social platforms than PADI-web). Please check, the Event detection section in Methods, lines 151-167 and in Results, lines 251-271.

Methodology

Event detection

First paragraph: We chose a one-year 131 study period (July 2018 - June 2019) to capture the space-time epidemiological characteristics of the AI outbreaks around the world.–¿ From which agencies?What sources?

The official data source is described further in our manuscript (Empres-i). Here, we meant that we wanted to embrace a time period enabling us to capture different epizootic events worldwide, to be able to compare the EBS tools and evaluate the network of sources based on a large number of AI outbreaks. Please check lines 151-165.

- We provide a new sentence in the Methods section: "We chose a one-year study period (July 2018 - June 2019) to capture larger scale AI outbreak patterns around the world." Please check lines 128-135.

Define about Empres-i - How it collects health data from official sources?

- We provide a more clear description of the EMPRES-i database, its purpose and its sources. Please

check the Event detection of the Materials and methods section, lines 151-165..

Second paragraph line 145, define what this acronym WOAH means. From this description you can mention only the acronym but not have defined yourself previously

- Done, we provide the full name of the World Organisation for Animal Health (WOAH, ex-OIE). Please check line 159.

Network construction

First paragraph "We assumed that an information pathway could be deducted from the sources cited in a news content. In an information pathway, the first node is called the primary source (i.e. the earliest emitter source), the last node is called the final source (i.e. the final aggregator, PADI-web or HealthMap) and the remaining nodes, if any, are called secondary sources." Comment: It is necessary to modify this definition because primary data in public health and epidemiology are those obtained directly in the territory to be sampled regarding a certain disease data. A secondary data are obtained through the country's information systems.

Epidemic intelligence (EI) encompasses all activities related to early identification of potential health hazards, their verification, assessment and investigation in order to recommend public health control measures. EI integrates both an indicator-based and an event-based component. 'Indicator-based component' refers to structured data collected through routine surveillance systems, corresponding to the definitions provided by the reviewer. 'Event-based component', the context of our study, refers to unstructured data gathered from sources of intelligence of any nature (e.g. media, laboratory, channels of communications, etc.,see `https://www.eurosurveillance.org/content/10.2807/esm.11.12.00665-en`). As noted by the reviewer, the primary sources in terms of diagnosis is usually a laboratory, even in EBS, especially when studying a well-known disease subject to notification as avian influenza. However, this is not true when the detected disease is not yet diagnosed and when solely information about unusual symptoms are communicated. This component of EBS, which is closed to the syndromic surveillance, is an essential component of early detection. In this study, we defined primary sources in EBS paradigm as the earliest cited source of each path, which is not necessarily the primary source in terms of diagnosis, but rather in terms of communication. Thus, it can include official sources typically involved in IBS (laboratory, country's official authorities), as well as informal sources (a person, an company, etc.). We have reworked the introduction, please check pages 3 and 4.

No reference to the global surveillance system by a specific WHO program was cited or used (`https://www.who.int/initiatives/global-influenza-surveillance-and-response-system` and `https://www.who.int/health-topics/influenza-avian-and-other-zoonotic`) Why?

Our study lies in the context of event-based surveillance in the animal health domain. We did not described World Health Organization surveillance programs as they mainly focus on zoonotic events from a public health perspective, in the indicator-based paradigm. Besides, our objective was to describe the EBS systems.

Official sources on animal and human surveillance should not be test sources for the network as they are the gold standard for comparing sources of risk communication. In this study, official sources on animal and human surveillance are not tested by themselves. They appeared in the network because they were cited by non-official sources monitored bu the EBS tools. For instance, if an online news sources stated "According to the WHOA, an outbreak of avian influenza was detected yesterday in country X", WHOA was the emitter (primary) source of our network.

Qualitative nodes analysis: Reformulate or change the terms referring to primary and secondary data that cannot refer to the EBS tools technique because they are intrinsically used terms. The terms used must be from epidemiology.

To our knowledge, this work is the first attempt to describe the dissemination of information between sources cited in online news in the context of health surveillance, and no specific terms where proposed to refer to such sources in the epidemiological context. Thus, we proposed the terms primary and secondary as they are explicit for the reader and reflect the temporal diffusion of the events.

How sensitive/specific is the PADI web and Health Map data compared to the gold standard of data? Where are the statistical analyzes showing this fact?

-We calculated the sensitivity of HealthMap and PADI-web, following the definition provided in section Methods. The specificity of event-based surveillance tools cannot be calculated, as it is impossible to assess the status of non-official events they detect; there may be false positive events, as well as true positive events not reported to the gold standard databases (WOAH and EMPRES-i). We did not provide any further statistical tests as the purpose of our study is not to evaluate the influence of factors in the sensitivity of the tools. Please check the apprach and the results in lines 168-181 and 276-278.

As for the geographic scope, it was not clear in the text to the national scope that the data refer. The data should cover the following variables: total number and frequencies of avian influenza events; mean, maximum and minimum value of the number of events monitored per epidemiological week; source and means of event notification; frequency of events monitored by region of occurrence and spatial distribution of events according to reference municipality; opportunity to notification; Closing opportunity (time interval between the date from the notification to the National Surveillance until the end of its monitoring) classification of the group of events according to means of transmission and risk classification after evaluation of the events

For the data from EBS tools, we did not chose any national scope a priori: our data selection was solely based on the studied disease (avian influenza) and host (animals) worldwide. To clarify, we added a table summarizing the total number and frequencies of avian influenza events; mean, maximum and minimum value of the number of events monitored per week; and the source of the event notification as Supplementary material.
