# Supplementary material for: Dissemination of information in event-based surveillance, a case study of Avian Influenza
Source: PLoS One. 2023 Sep 5;18(9):e0285341. doi: 10.1371/journal.pone.0285341 (PMC10479896; doi:10.1371/journal.pone.0285341)
Supplement: Attachment — Submitted filename: Response to reviewers round2.pdf [file pone.0285341.s016.pdf]

April 12, 2023

Subject: Response to the review of manuscript number PONE-D-22-24102

Dear PlosOne Chief Editor and Reviewers,

We acknowledge your comments on our manuscript "Dissemination of information in event-based surveillance, a case study of Avian Influenza". We addressed the remaining issues in our manuscript (using track changes) and provide a response to the reviewers' questions here-below. Please note that we detected an artefact in Figure 4 which was re-exported.

Best regards,
The authors

Comments from reviewer 1

Whats the standardizing of the "relevant news and irrelevant news"?

We did not standardize relevant and irrelevant news. We categorized them manually by relevance. As described in the manuscript, relevant news was describing a disease outbreak and irrelevant news was talking about another topic, irrelevant to a disease outbreak. Thus we did not need to standardise the news, we just categorized them.

We evaluated the Se? What is this? There is no acronym that explains it?

We referred to the sensitivity (Se) before defining the acronym. We fixed the issue. In the event-based surveillance context, sensitivity corresponds to the number of official events detected by the EBS system.

How can the padi web software be considered more sensitive than Health map when there is no kappa value, or fisher test or other robust statistics used in the analyses?

We added a statistical test (McNemar's test on paired nominal data for, i.e., to compare the proportions for two correlated dichotomous variables) to evaluate the significance of the difference between the sensitivity (Se), at the standard significance level of 5% (the analysis is provided as supplementary material, S1 File.pdf). We provided precision in the discussion, in the phrase on the sensitivity between the two systems.
